# Molecular Recognition and Chirality Sensing with Metallocene Dichloride Complexes

**DOI:** 10.1021/acs.joc.6c01097

**Published:** 2026-08-05

**Authors:** Yujin Kim, Jeffrey S. S. K. Formen, Christian Wolf

**Affiliations:** Chemistry Department, 8368 Georgetown University, Washington, District of Columbia 20057, United States

## Abstract

The utility of well-defined transition-metal coordination complexes for separation-free enantioselective optical analysis has received significant traction due to its promise to speed up chiral compound screening workflows. Herein, chiroptical sensing of a broad selection of carboxylic acids with metallocene dihalides is introduced. Cp_2_TiCl_2_ and Cp_2_MoCl_2_ undergo smooth chloride exchange under mild conditions toward carboxylate complexes, displaying quantifiable circular dichroism signals at long wavelengths. Spectroscopic titration experiments and crystallographic analysis show that both chlorides in Cp_2_TiCl_2_ are readily displaced, while Cp_2_MoCl_2_ forms a monocarboxylate complex. In either case, characteristic CD inductions are observed that allow accurate *er* analysis across a wide range of enantiomeric compositions.

## Graphical abstract

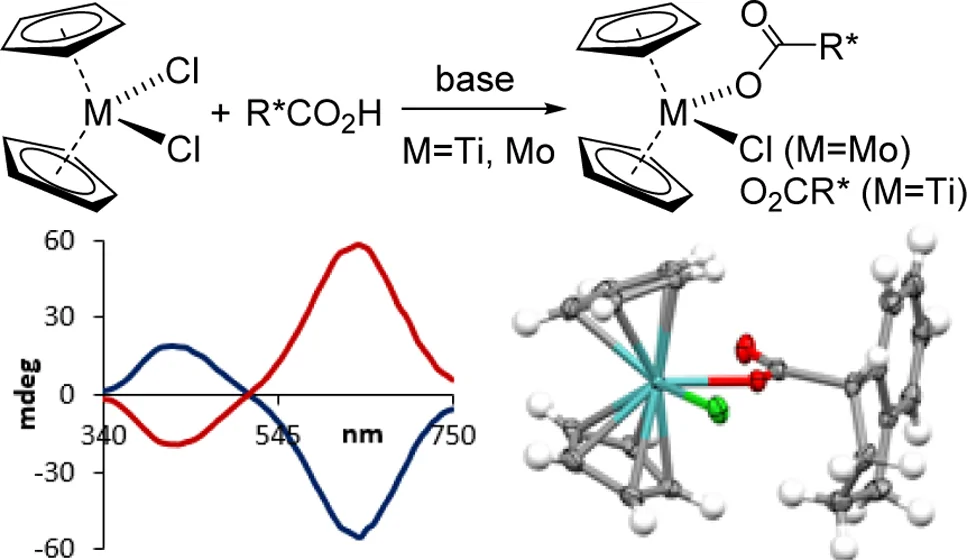

## Introduction

Metallocene dihalides, Cp_2_MX_2_, have been known for a long time and intensively investigated because of their intriguing structures, use as organometallic catalysts or reagents, and medicinal properties, among which anticancer activities have received substantial attention.^1–4^ Numerous studies have been conducted with bis­(η^5^-cyclopentadienyl)titanium dichloride^5–9^ and its molybdocene analog,^10–15^ showing their ease of handling, unique chemical reactivity at the cyclopentadienyl rings, and facile access to chemically diverse, air-stable structures produced by smooth halide replacement. In particular, the practical access to a large variety of lipophilic metallocenes that are inert in aqueous solutions via halide exchange reactions has unleashed the pursuit of a wide range of biomedical applications. While the readily available metallocene dichlorides Cp_2_TiCl_2_ and Cp_2_MoCl_2_, which are bright-red and brownish-green solids, have been popular starting materials and sparked widespread interest across the organometallic, bioinorganic, and medicinal chemistry realms for many decades, their striking spectroscopic properties have been scarcely exploited and remain underdeveloped to date.

The formation of metal coordination complexes of chiral compounds has been successfully exploited in many ways for optical enantiomeric ratio (*er*) sensing.^16^ Recently, we showed that piano stool complexes like [Cp*Ru­(CH_3_CN)_3_]­PF_6_ undergo fast and irreversible acetonitrile displacement with chiral aromatic molecules toward sandwich complexes displaying circular dichroism (CD) signals that can be correlated to the absolute configuration and *er* of the captured analyte, Figure 1.^17^ The strong CD inductions observed with η^6^-arene coordination complexes suggested to us that metallocenes may have a broader application scope and could be applicable to other compound classes as well. To the best of our knowledge, optical chirality sensing with metallocene dihalides has not been reported. Based on the well-known high oxophilicity of Ti­(IV) and Mo­(IV) compounds, we envisioned that substitution of the chlorides in the corresponding Cp_2_MCl_2_ metallocenes with carboxylate groups should be thermodynamically favored and occur under mild conditions. A well-defined stoichiometric ligand exchange was thus expected to create an excellent opportunity for molecular recognition and enantioselective analysis of chiral carboxylic acids in the presence of a weak base to neutralize the HCl byproduct.

**Figure 1:**
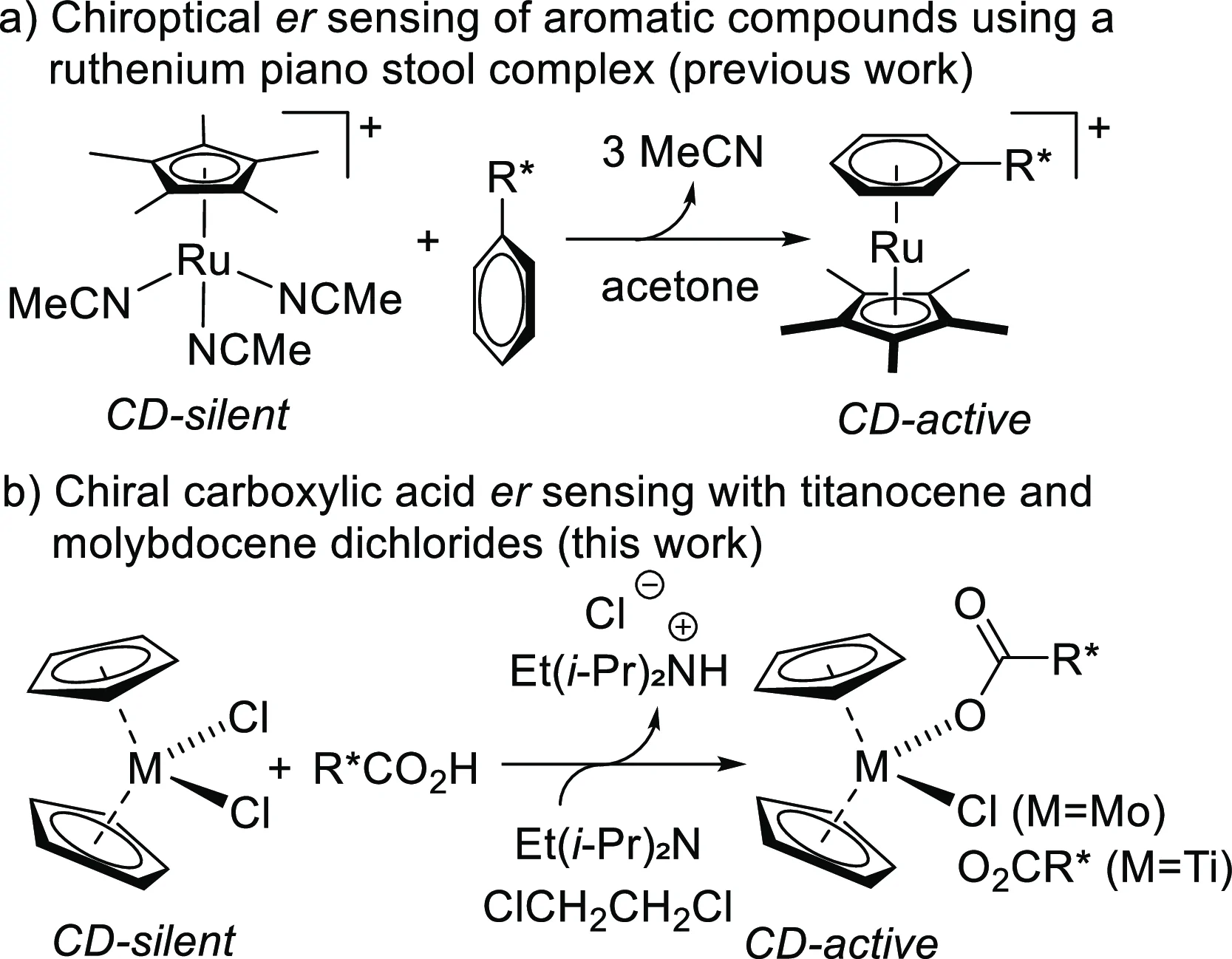
Chirality sensing with piano stool complexes (a) and metallocene dihalides (b).

The search for small-molecule chemosensors suitable for quantitative *er* analysis is receiving increasing attention. In order to determine the enantiomeric ratio of chiral compounds, chemists tend to resort to chromatographic and nuclear magnetic resonance (NMR) spectroscopic protocols. However, these typically do not match high-throughput screening expectations, i.e., they do not allow fast and preferably parallel analysis of hundreds of samples at once. As a result, a variety of mass spectrometry,^18^ UV,^19^ fluorescence,^20–22^ gas-phase rotational resonance,^23^ infrared,^24^ electronic CD,^25–31^ fluorescence-detected CD spectroscopy,^32^ and biochemical assays^33^ have been developed as powerful alternatives.^34^ Many CD methods have been tailored toward chiral recognition of α-amino acids^35–51^ and α-hydroxy acids,^52–60^ which exhibit two functional groups that can be used in concert for well-defined binding and signal induction and are therefore privileged structures. By contrast, monofunctional carboxylic acids have remained more difficult targets although various assays based on metal coordination,^61–67^ hydrogen bonding,^68–70^ and salt bridge formation^71^ are known.

## Results and Discussion

To assess the suitability of metallocene dihalides for chiral carboxylic acid sensing, we decided to mix either Cp_2_TiCl_2_, **1**, or Cp_2_MoCl_2_, **2**, with enantiopure test analytes using a variety of solvents and bases while also exploring the stoichiometry of the proposed halide exchange reaction. These mixtures were then subjected to CD analysis for the initial analysis of successful carboxylate binding and to determine if coordination would indeed result in the induction of strong and potentially quantifiable CD signals. We were pleased to observe strong CD signals, for example, with ibuprofen, **3**, that arise in the presence of (*i*-Pr)_2_NEt, Et_3_N, pyridine, K_2_CO_3_, or NaO*t*-Bu in several organic solvents, Figure 2 and Supporting Information. Screening of the reaction conditions revealed that the use of amine bases in chlorinated solvents gives excellent results with the titanocene dihalide **1**, and the carboxylate coordination was confirmed by ^1^H NMR titration experiments, see Supporting Information. Due to the low solubility of the molybdenum compound **2**, we resorted to potassium carbonate and *N*,*N*-dimethylformamide (DMF) as the solvent. Again, we found that the addition of the enantiomers of ibuprofen generates strong and remarkably red-shifted CD signals, which are generally preferred in the CD sensing field because they avoid complications with potentially present chiral impurities that typically give weak but sometimes not negligible CD curves at shorter wavelengths. We are not aware of previous reports on chiroptical sensing studies with the metallocene dichlorides **1** and **2**, but we note that dimolybdenum complexes have previously been demonstrated to allow absolute configuration determination of chiral diols.^72^ Interestingly, the CD inductions obtained upon coordination of ibuprofen to **1** and **2** are strikingly different. While chiroptical sensing of (*S*)-**3** with Cp_2_TiCl_2_ results in a single positive CD maximum, it yields two negative maxima with Cp_2_MoCl_2_. The reverse situation is, as expected, observed when (*R*)-**3** is used, see the spectra shown in blue and red, respectively, in Figure 2. Although the CD sensing experiments were conducted under different conditions, we suspected that these unexpected outcomes could not simply be attributed to solvent or base effects but may be a result of different binding motifs. Indeed, CD titration experiments showed that the intensity of the produced CD signal nearly doubles when two equiv of base and the chiral analyte were added to **1**, suggesting that both chlorides are replaced. By contrast, essentially the same CD spectra were obtained even after several hours when **2** was treated with either one or two equivalents of base and chiral analyte, which indicates that only one chloride is substituted and a monocarboxylate complex is formed, see Supporting Information and below.

**Figure 2:**
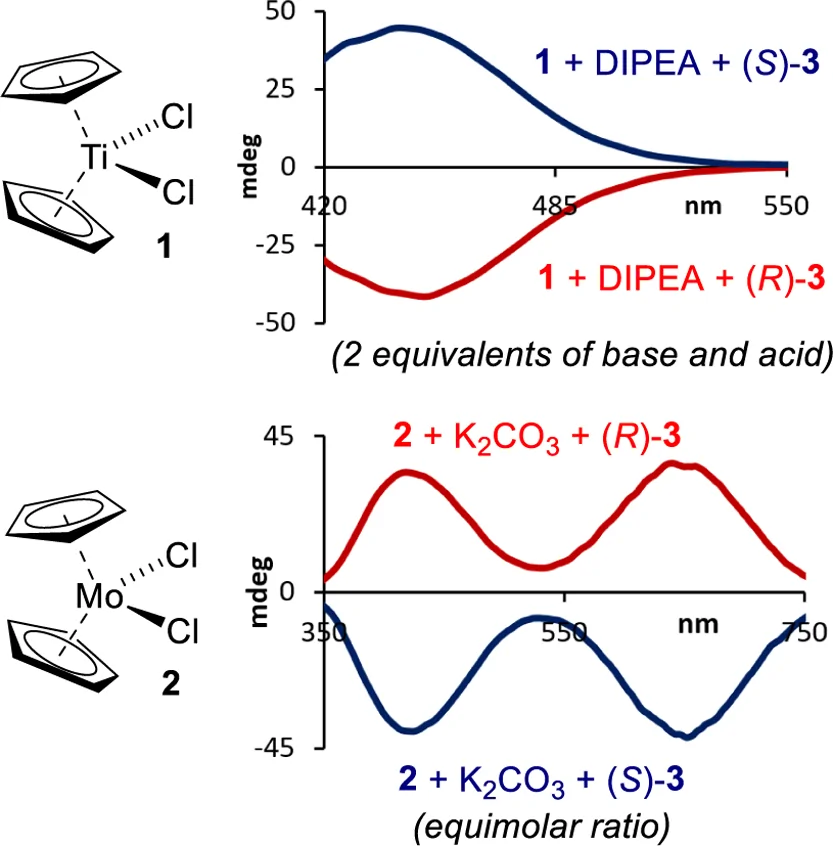
Structures of the sensors and CD inductions obtained by coordination of the enantiomers of ibuprofen, **3**, to titanocene dichloride and molybdocene dichloride in the presence of diisopropylethylamine in dichloroethane and K_2_CO_3_ in DMF, respectively.

With proof-of-concept results in hand, we continued to evaluate the sensing scope of titanocene dihalide **1** under optimized conditions. For this purpose, we obtained a wide range of chiral substrates including purely aliphatic carboxylic acids but also increasingly complicated, multifunctional compounds and biologically relevant ones like dihydroartemisinic acid, **13**, dehydroabietic acid, **14**, and isosteviol, **15**, see Figure 3 and Supporting Information. In most cases, strong CD signal inductions above 400 nm were measured as exemplified with **5**, **11**, **15**, and **16**, which demonstrates the general usefulness of chiral carboxylic acid recognition with this probe. It is noteworthy that substrates carrying an aromatic ring or a rigid scaffold like **3**, **5**, **6**, **11**, **16**, and **19** generally give quite strong CD effects, whereas structures like **9**, **10**, **17**, and **18** exhibited weak signal inductions.

**Figure 3:**
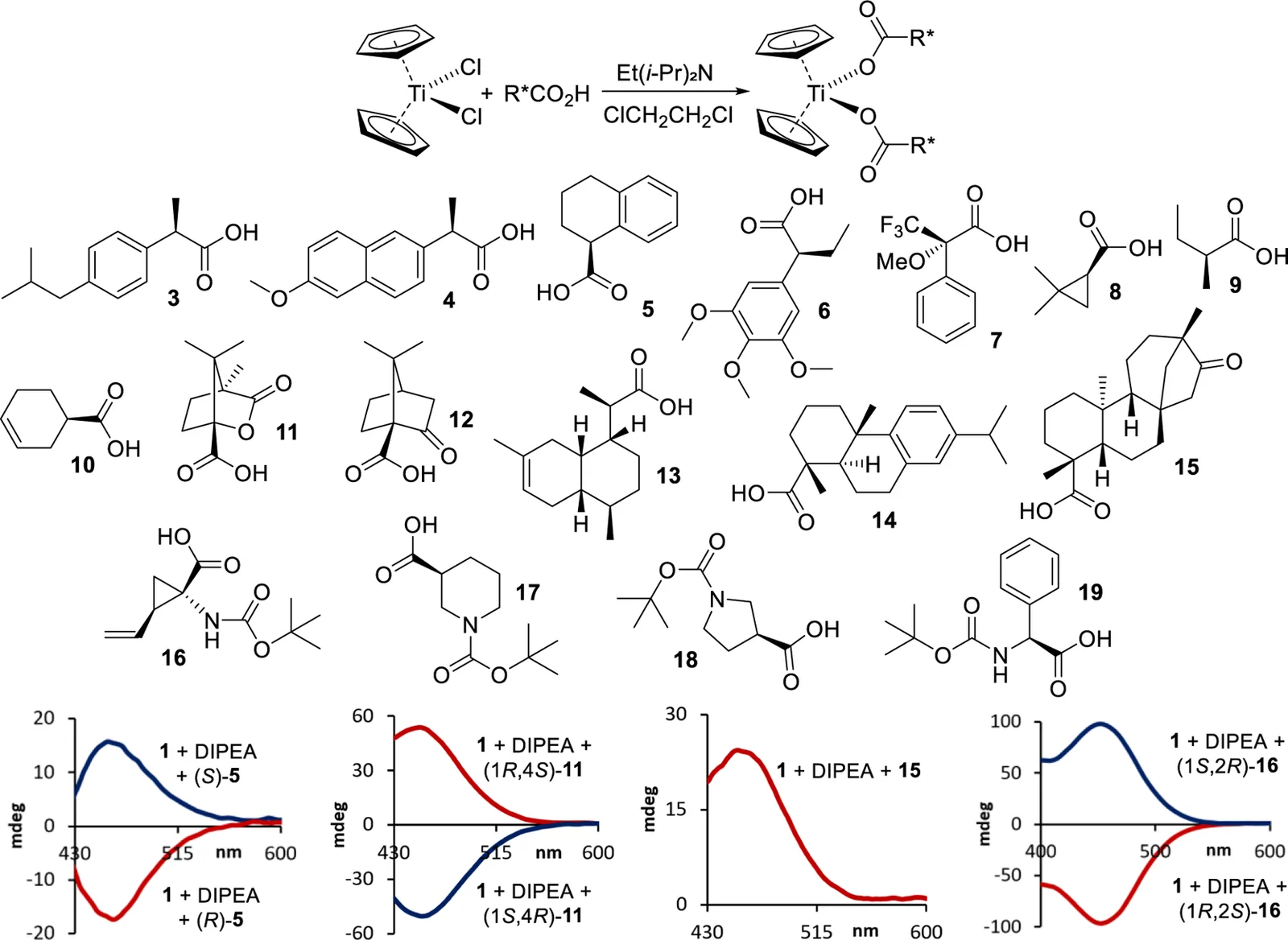
Structures of carboxylic acids tested and selected ICD spectra obtained with sensor **1**. All CD measurements were taken at 5.0 mM in DCE. See Supporting Information for details.

The broad interest in chiroptical CD sensing undoubtedly originates from its unique promise in separation-free enantiomeric-composition-determination applications. We therefore selected *t*-Boc-protected phenylglycine **19** to investigate the possibility of enantiomeric ratio (*er*) analysis. Having established that the induced CD signal intensity increases linearly with the enantiomeric excess of **19**, we applied nine arbitrarily composed scalemic samples of this acid in our sensing assay and used the intensity of the maximum at 465 nm to determine the corresponding *er* values via linear regression analysis, Table 1. Comparison of the actual and experimentally obtained data shows excellent agreement in cases where one enantiomer is in large excess but also at low enantiomeric ratios. For example, the sensing of a 93.7:6.3 (*S*)/(*R*) mixture gave a 93.6:6.4 ratio, and the analysis of a sample containing 38.8% of (*S*)-**19** and 61.2% of the (*R*)-enantiomer gave 39.7% and 60.3%, respectively.

**Table 1:**
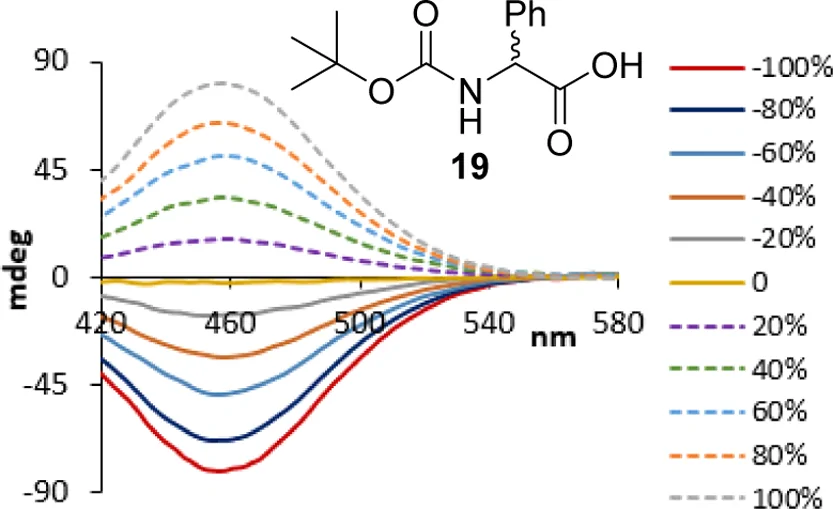
Chirality Sensing of Scalemic Mixtures of **19** with **1** ^a^

sample composition		sensing results	
AC	*er*	AC	*er*
*S*	93.7:6.3	*S*	93.6:6.4
*S*	80.0:20.0	*S*	82.2:17.8
*S*	63.7:36.3	*S*	65.0:35.0
*S*	57.5:42.5	*S*	57.8:42.2
*R*	38.8:61.2	*R*	39.7:60.3
*R*	32.5:67.5	*R*	32.0:68.0
*R*	25.0:75.0	*R*	24.6:75.4
*R*	16.2:83.8	*R*	15.5:84.5
*R*	8.7:91.3	*R*	7.4:92.6

a AC is the absolute configuration of the major enantiomer. See Supporting Information for details.

We then turned our attention to molybdocene **2**. Following the methodical approach described above with the titanium probe, we first applied a variety of chiral carboxylic acids in the optimized sensing assay and found that Cp_2_MoCl_2_ is similarly useful. Using equimolar amounts of **2**, an acid, and potassium carbonate in DMF gave strong, red-shifted CD effects as initially observed with ibuprofen. Representative examples of the observed CD inductions obtained with **5**–**7** and **16** are shown in Figure 4. We found that carboxylic acids that gave the strongest CD effects with the titanocene sensor also performed the best in this assay.

**Figure 4:**
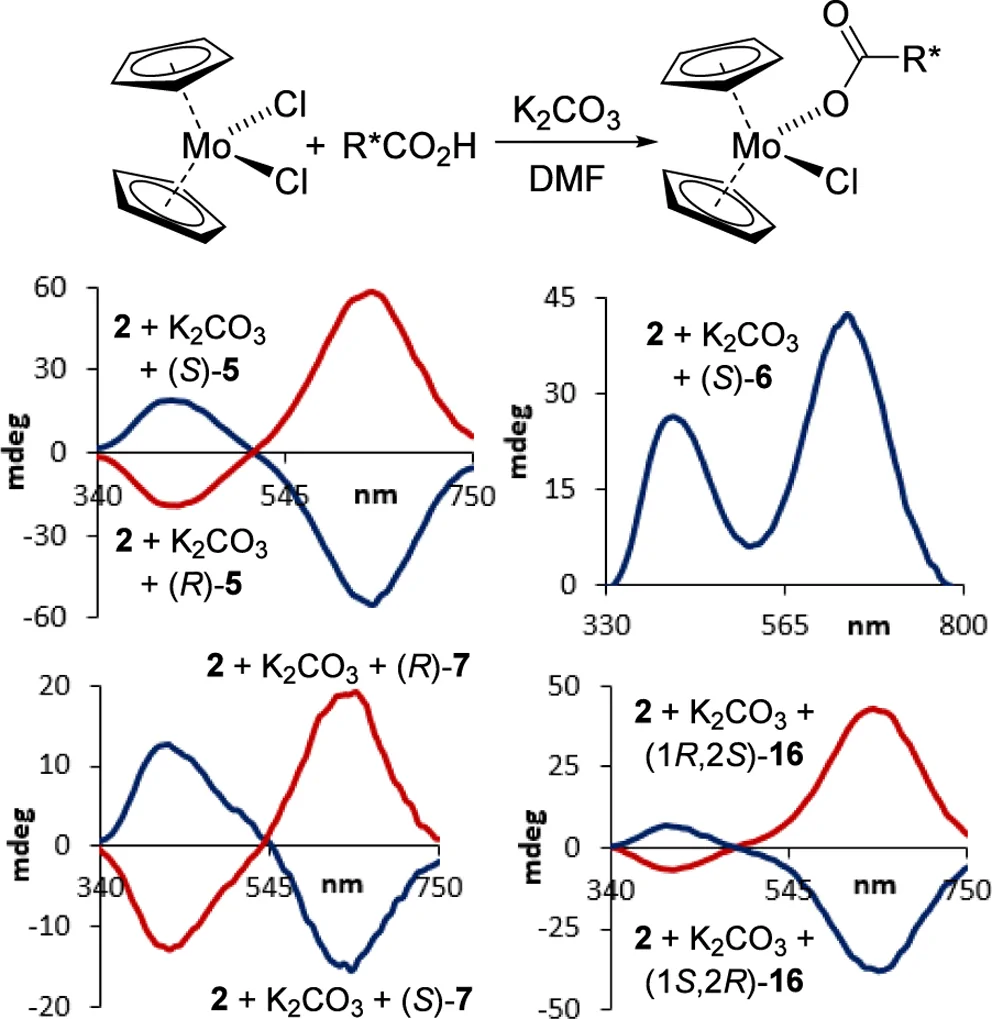
Selected ICD spectra obtained with sensor **2**. All CD measurements were taken at 5.0 mM in DMF. See Supporting Information for details.

The suitability of **2** for quantitative *er* determination was examined with 1,2,3,4-tetrahydronaphthalene-1-carboxylic acid, **5**, as shown in Figure 5. As expected, we found that the CD amplitudes at 420 and 645 nm increase linearly with the enantiomeric excess of **5**. With knowledge of the correlation between the sample composition and the induced CD signal intensities, we then tested our sensing assay using the stronger signals at 645 nm to determine *er* values of nine nonracemic samples, Table 2. Again, the actual and experimentally obtained values show very good agreement. For example, the sensing of a 5.0:95.0 (*S*)/(*R*) mixture gave a 6.0:94.0 ratio, and the analysis of a sample containing 45.0% of (*S*)-**19** and 55.0% of the (*R*)-enantiomer gave 43.8% and 56.2%, respectively.

**Figure 5:**
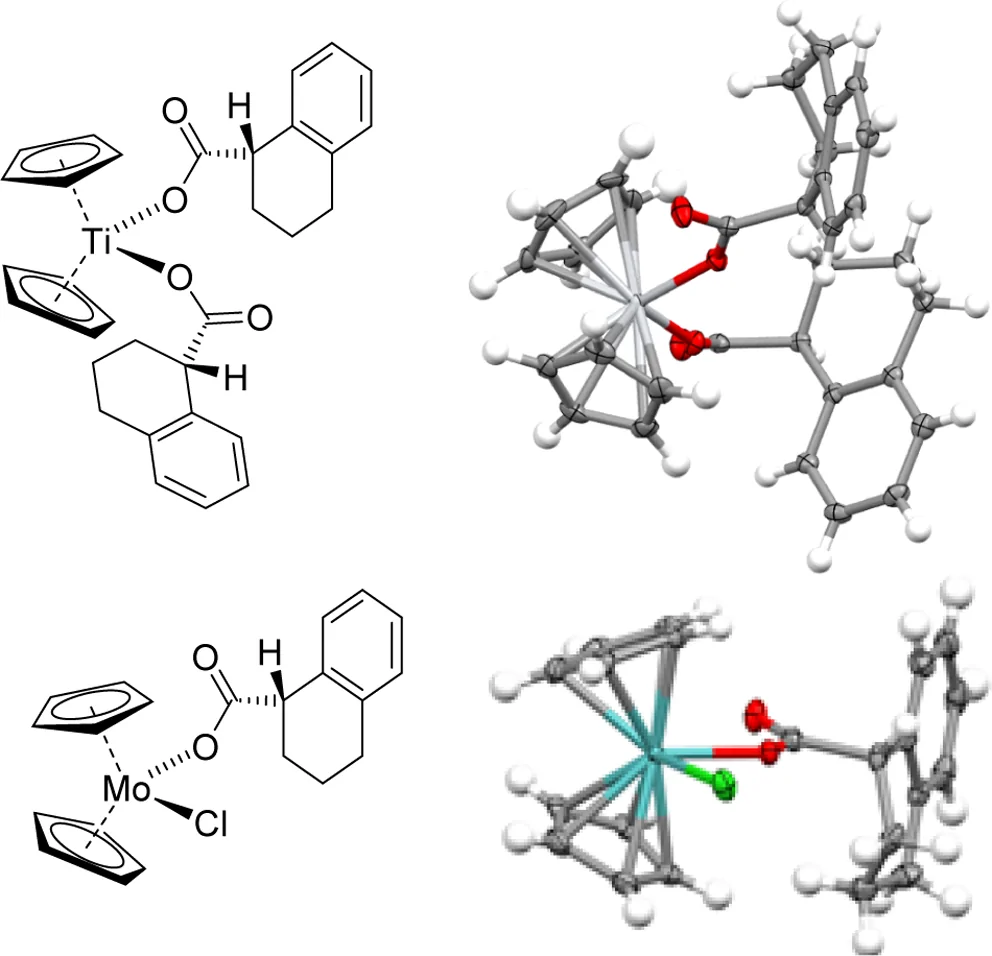
X-ray structures of Cp_2_Ti­[(*S*)-**5**]_2_ and Cp_2_MoCl­(*S*)-**5**.

**Table 2:**
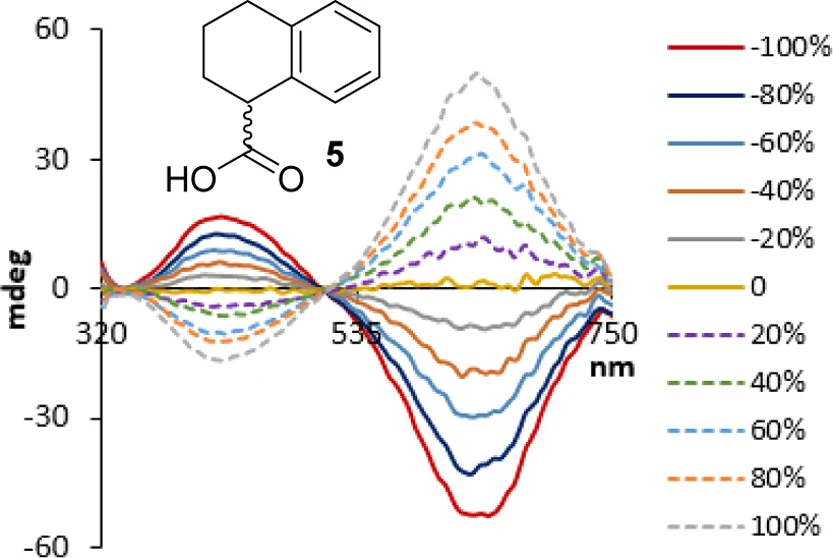
Chirality Sensing of Carboxylic Acid **5** Samples with **2** ^a^

sample composition		sensing results	
AC	*er*	AC	*er*
*R*	5.0:95.0	*R*	6.0:94.0
*R*	12.5:87:5	*R*	12.8:87.2
*R*	18.8:81.2	*R*	21.7:78.3
*R*	25.0:75.0	*R*	22.2:77.8
*R*	37.5:62.5	*R*	34.7:65.3
*R*	45.0:55.0	*R*	43.8:56.2
*S*	56.2:43.8	*S*	53.9:46.1
*S*	62.5:37.5	*S*	60.4:39.7
*S*	83.7:16.3	*S*	82.8:17.2

a AC = absolute configuration. See Supporting Information for details.

Finally, we decided to further corroborate the different binding stoichiometries with the two metallocenes, **1** and **2**. A literature survey revealed that Cp_2_TiCl_2_ indeed produces dicarboxylates under conditions similar to our assay, thus confirming that both chlorides are replaced, which was demonstrated by single-crystal analysis of a dibenzoate complex.^73^ This encouraged us to attempt single-crystal growth with chiral carboxylate complexes of the two metallocene probes. For this purpose, we chose **5**, because we expected that the relatively small size and rigid structure of this carboxylic acid would facilitate effective solid-state packing and therefore be more promising than the others. We were successful with producing a single crystal of Cp_2_Ti­[(*S*)-**5**]_2_ suitable for crystallographic analysis, and we were pleased to find that this was also possible with the molybdenum compound. The molybdocene dichloride and two equivalents of (*S*)-**5** and K_2_CO_3_ were stirred at room temperature in DMF to closely mimic our sensing protocol and to allow formation of both Cp_2_MoCl­(*S*)-**5** and Cp_2_Mo­[(*S*)-**5**]_2_. After 2 h, the mixture was filtered and concentrated *in vacuo*. The residue was dissolved in CH_2_Cl_2_, and pentane was carefully layered onto the resulting solution to afford single crystals. Despite the excess of base and carboxylate present in solution, the monocarboxylate complex showing one remaining chloride was obtained, as shown in Figure 5. The crystallographic analyses of the isolated single crystals are thus in agreement with CD titration experiments.

Attempts to generate a molybdenum dicarboxylate complex under significantly harsher conditions were unsuccessful. When Cp_2_MoCl_2_ was treated with excess of **5** and base at 70 °C for 2 h, the resulting CD signal was identical to that of the monocarboxylate complex, confirming that the second substitution does not occur under these conditions. The addition of silver triflate to abstract the remaining chloride from the molybdenum center did coincide with the expected AgCl precipitation but we were not able to identify a molybdenum dicarboxylate complex and the resulting solution gave only a weak CD signal.

To determine if the carboxylate binding is reversible and if formation of heterochiral titanium complexes is possible, we conducted several CD titration experiments. We prepared the homochiral complex Cp_2_Ti­[(*S*)-**20**]_2_ following our general assay protocol. This gave the expected CD induction with a strong positive signal, as shown in Figure 6. One equivalent of the (*R*)-acid was then added, and after stirring for several hours, the CD amplitude decreased to the value predicted for a fully equilibrated 33% *ee* mixture. The CD essentially disappeared upon addition of a second (*R*)-**20** equivalent, as expected for a racemic mixture at equilibrium. This was reproduced with the opposite homochiral Cp_2_Ti­[(*R*)-**20**]_2_ complex. These titration experiments clearly demonstrate that the system is dynamic and undergoes a reversible ligand exchange. The CD changes are in agreement with a mechanism involving a heterochiral dicarboxylate complex that is formed by stepwise replacement of one carboxylate at a time, as simultaneous displacement of two (*S*)-ligands from the titanium­(IV) center by two (*R*)-carboxylates is unlikely.

**Figure 6:**
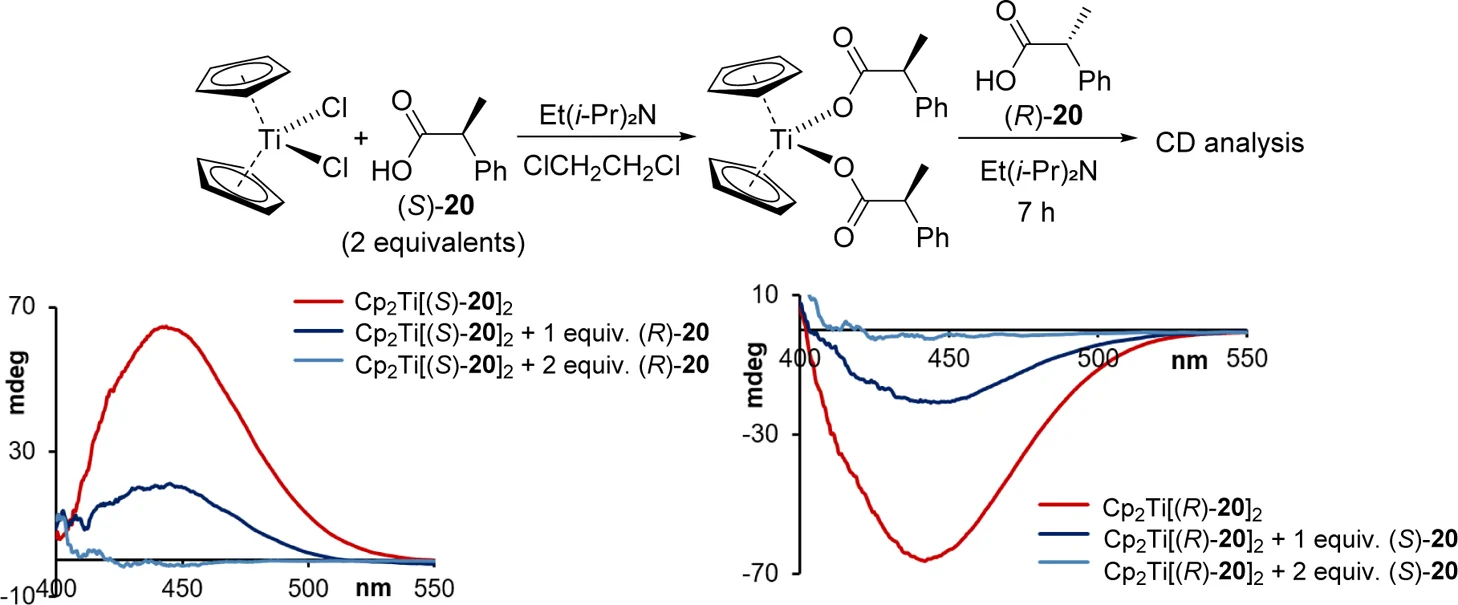
CD titration experiments with Cp_2_Ti­[(*S*)-**20**]_2_ (left) and Cp_2_Ti­[(*R*)-**20**]_2_ (right).

In summary, the possibility of enantioselective chiral compound CD sensing with metallocene dihalides has been demonstrated for the first time. Cp_2_TiCl_2_ and Cp_2_MoCl_2_ undergo chloride exchange in the presence of base and chiral carboxylic acids under mild conditions, and the corresponding carboxylate complexes display distinct CD signals at long wavelengths. Spectroscopic titration experiments and crystallographic analysis showed that both chlorides in Cp_2_TiCl_2_ are readily displaced, while Cp_2_MoCl_2_ forms a monocarboxylate complex. The screening of a broad selection of analytes, including profens and other biologically active compounds, showed that these sensors are generally useful. The strong CD inductions obtained simply by mixing the metallocene dihalides with base and acid can be used for accurate *er* analysis, which was demonstrated with a total of 18 samples covering a wide range of enantiomeric compositions. All assay components are commercially available, which further highlights the practicality of this optical method. It is noteworthy that this type of assay is amenable to commercial multiwell plate readers, which would allow high-throughput analysis of large numbers of samples and avoid the use of time-consuming, one-at-a-time chromatographic separation methods unless molecular interferents that compete with the complex formation are present.

## Experimental Section

Bis­(cyclopentadienyl)­titanium dichloride is a moisture-sensitive compound, while bis­(cyclopentadienyl)molybdenum dichloride is stable to water. However, both chemicals were stored under an inert atmosphere in a glovebox. All sensing reactions were carried out under an inert atmosphere with anhydrous solvents. The solutions of the corresponding carboxylate complexes are stable under air for several hours and were subjected to CD analysis without additional precautions.

### General Remarks

All commercially available reagents were used without any further purification. All of the sensing reactions were carried out under nitrogen. The CD spectra were collected with a standard sensitivity of 100 mdeg, a data pitch of 0.5 nm, and a bandwidth of 1.0 nm in a continuous scanning mode with a scanning speed of 500 nm/min and a response of 1 s (1 cm path length). The data were baseline-corrected and smoothed using a binomial equation.

### CD Sensing

#### Carboxylic Acid Sensing with Sensor 1

To 0.20 mL of a 0.10 M stock solution of the acid in 1,2-dichloroethane (DCE) was added 44.0 μL of a 0.50 M (*i*-Pr)_2_NEt stock solution in DCE. Then, 1.6 mL of a 6.25 mM sensor **1** stock solution in DCE and 0.156 mL of the same solvent were added to each vial. The solutions were stirred for 3 h at 25 °C, and CD analysis was performed directly with the reaction mixtures (5.0 mM) without further dilution.

#### Carboxylic Acid Sensing with Sensor 2

Into a glass vial, 0.40 mL of a 25.0 mM solution of the acid in DMF was added, followed by 1.1 equiv of K_2_CO_3_. Then, 1.6 mL of a 6.25 mM sensor **2** stock solution in DMF was added. The mixtures (5.0 mM) were stirred for 1 h at 25 °C, and CD analysis was performed without further dilution.

The carboxylate complexes are stable for several hours in solution under air, which gives sufficient time to conduct the sensing experiments. However, racemization of the carboxylic acid can occur when the basic solutions are concentrated.

## Supplementary Material



## Data Availability

The data underlying this study are available in the published article and its Supporting Information.

## Notes

Assay development, optimization, and crystallographic and analytical details (PDF)
