# Supplementary material for: Molecular Recognition and Chirality Sensing with Metallocene Dichloride Complexes
Source: J Org Chem. 2026 Aug 5;91(32):10933–40. doi: 10.1021/acs.joc.6c01097 (PMC13488449; doi:10.1021/acs.joc.6c01097)

Molecular Recognition and Chirality Sensing with Metallocene Dichloride Complexes

Yujin Kim, Jeffrey S. S. K. Formen and Christian Wolf*

Chemistry Department, Georgetown University, Washington DC 20057, USA.

Contents

1. General information S2
2. Optimization of sensing conditions and reaction analysis for sensor **1** S2
3. Carboxylic acid sensing scope of sensor **1** S7
4. Carboxylic acid *er* sensing with sensor **1** S14
5. Optimization of sensing conditions for sensor **2** S16
6. Carboxylic acid sensing scope of sensor **2** S19
7. Carboxylic acid *er* sensing with sensor **2** S25
8. X-ray crystallography S27
9. CD titration and stability experiments S29
10. HR-MS results S31

1. General information

All commercially available reagents were used without further purification. All sensing reactions were carried out under nitrogen. NMR spectra were obtained at 400 MHz (^{1}H NMR). Chemical shifts are reported in ppm relative to the solvent peak. The CD spectra were collected with a standard sensitivity of 100 mdeg, a data pitch of 0.5 nm, and a bandwidth of 1.0 nm in a continuous scanning mode with a scanning speed of 500 nm/min and a response of 1 s (1 cm path length). The data were baseline corrected and smoothed using a binomial equation.

Figure S1. Structures of the sensors and analytes. Only one stereoisomer is shown.

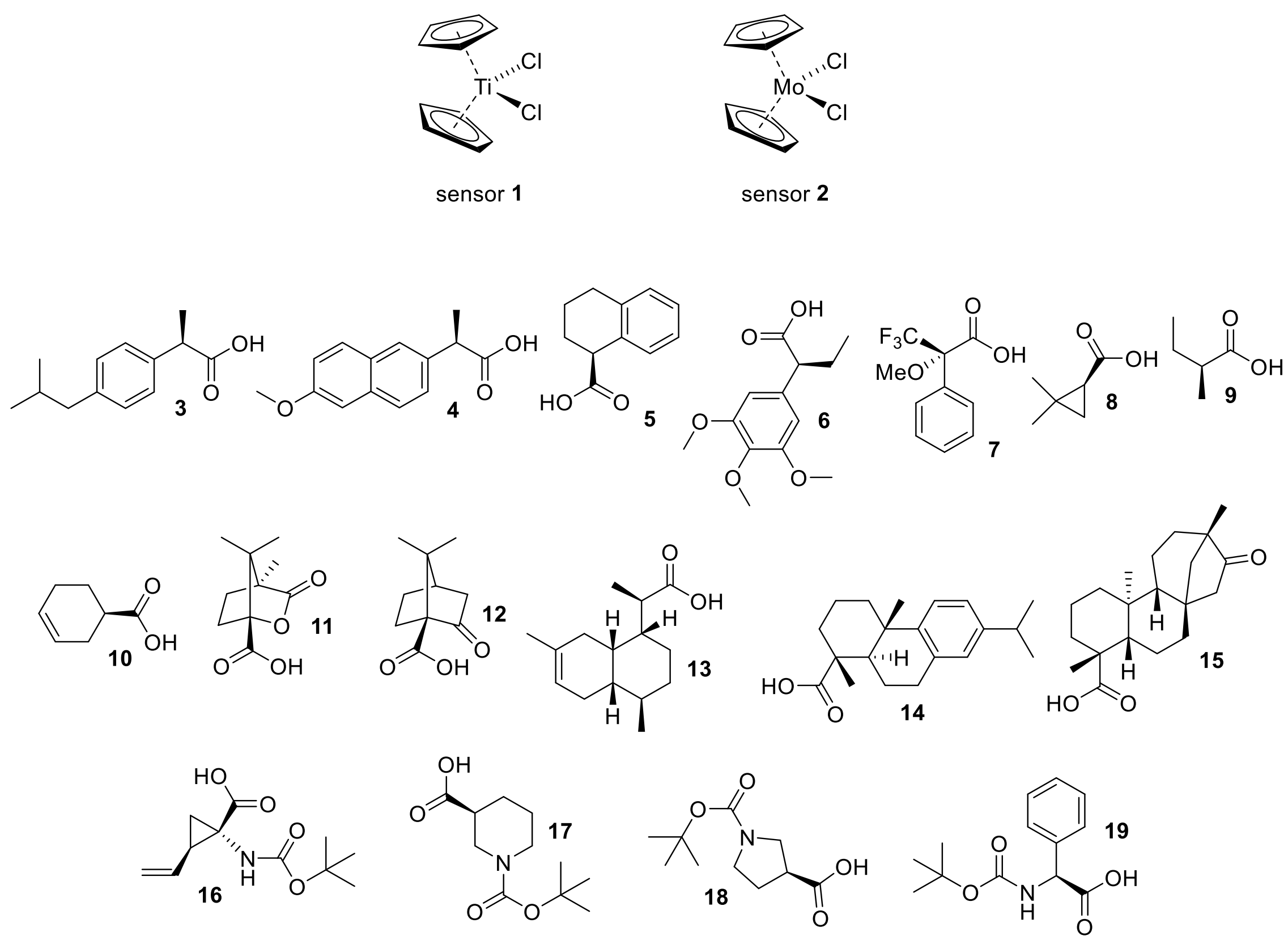

2. Optimization of sensing conditions and reaction analysis for sensor 1

In a glass vial, 1.6 mL of a 6.25 mM sensor **1** stock solution in 1,2-dichloroethane (DCE) were mixed with either 0.20 mL or 0.40 mL of a 50.0 mM (*R*)-**19** stock solution in DCE, and 44.0 µL of a 0.50 M (*i*-Pr)$_2$NEt stock solution in DCE. For the 0.20 mL (*R*)-**19** sample, an additional 0.20 mL of DCE was added. The mixtures were stirred for 3 hours under nitrogen.

Scheme S1. Effect of acid equivalence on the ICD with sensor **1**.

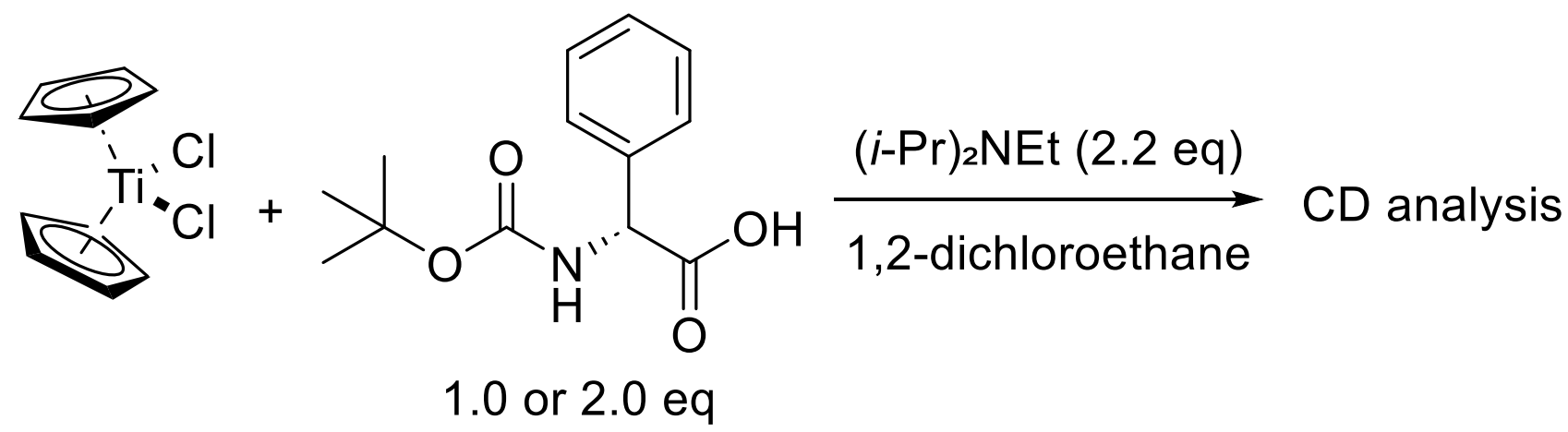

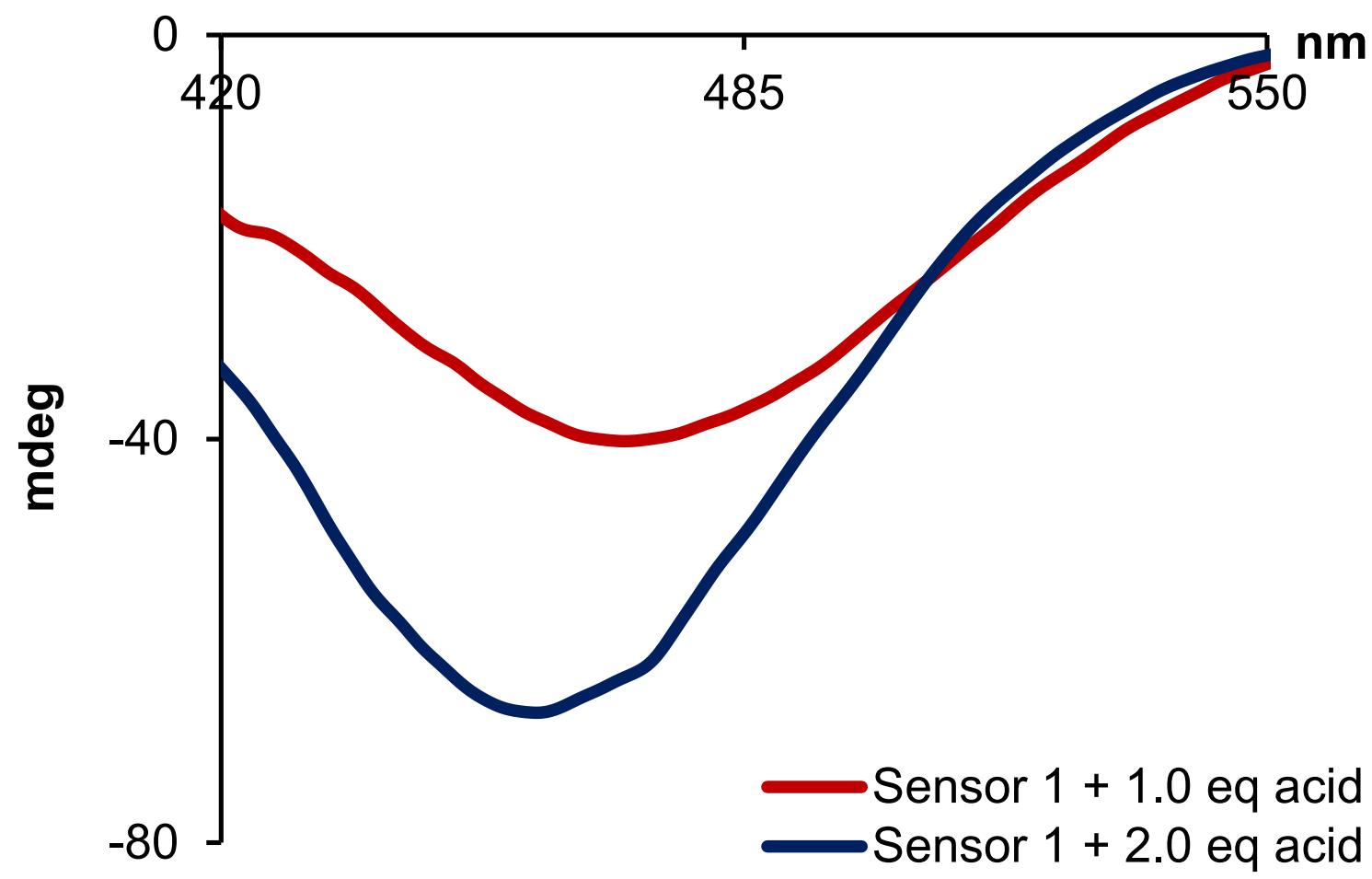

CD measurements were taken at 5.0 mM in 1,2-dichloroethane.

In a glass vial, 1.0 mL of a 10.0 mM sensor **1** stock solution in CH_2Cl_2 was mixed with 1.0 mL of a 20.0 mM (*S*)-ibuprofen stock solution in CH_2Cl_2. (*i*-Pr)$_2$NEt and Et_3N (20.0 µmol each) were added as 40.0 µL of 0.50 M stock solutions in CH_2Cl_2. Pyridine, K_2CO_3, and NaO*t*-Bu were each added in amounts of 20.0 µmol. The mixtures were stirred for 18 hours under nitrogen. The strongest ICD signal was observed when either (*i*-Pr)$_2$NEt, Et_3N, or K_2CO_3 was used as the base.

Scheme S2. Effect of base on the ICD with sensor **1**.

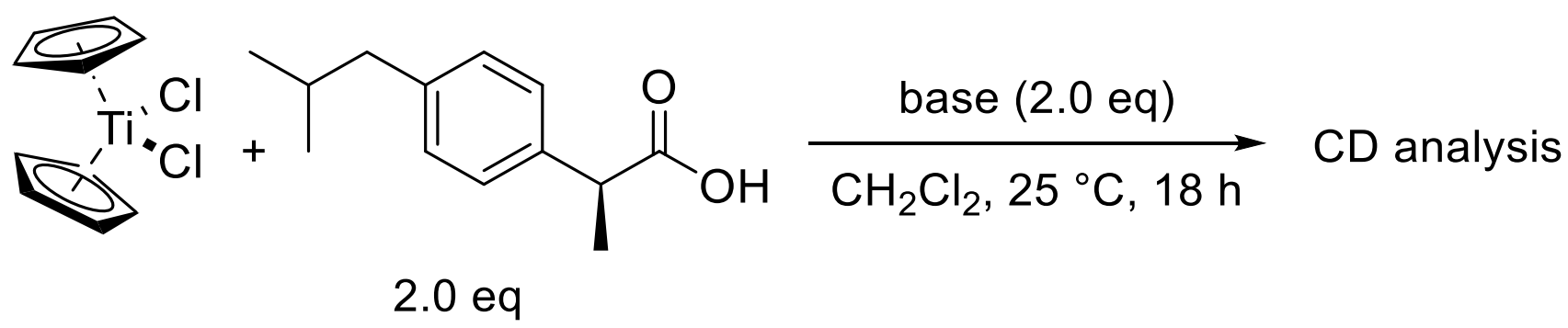

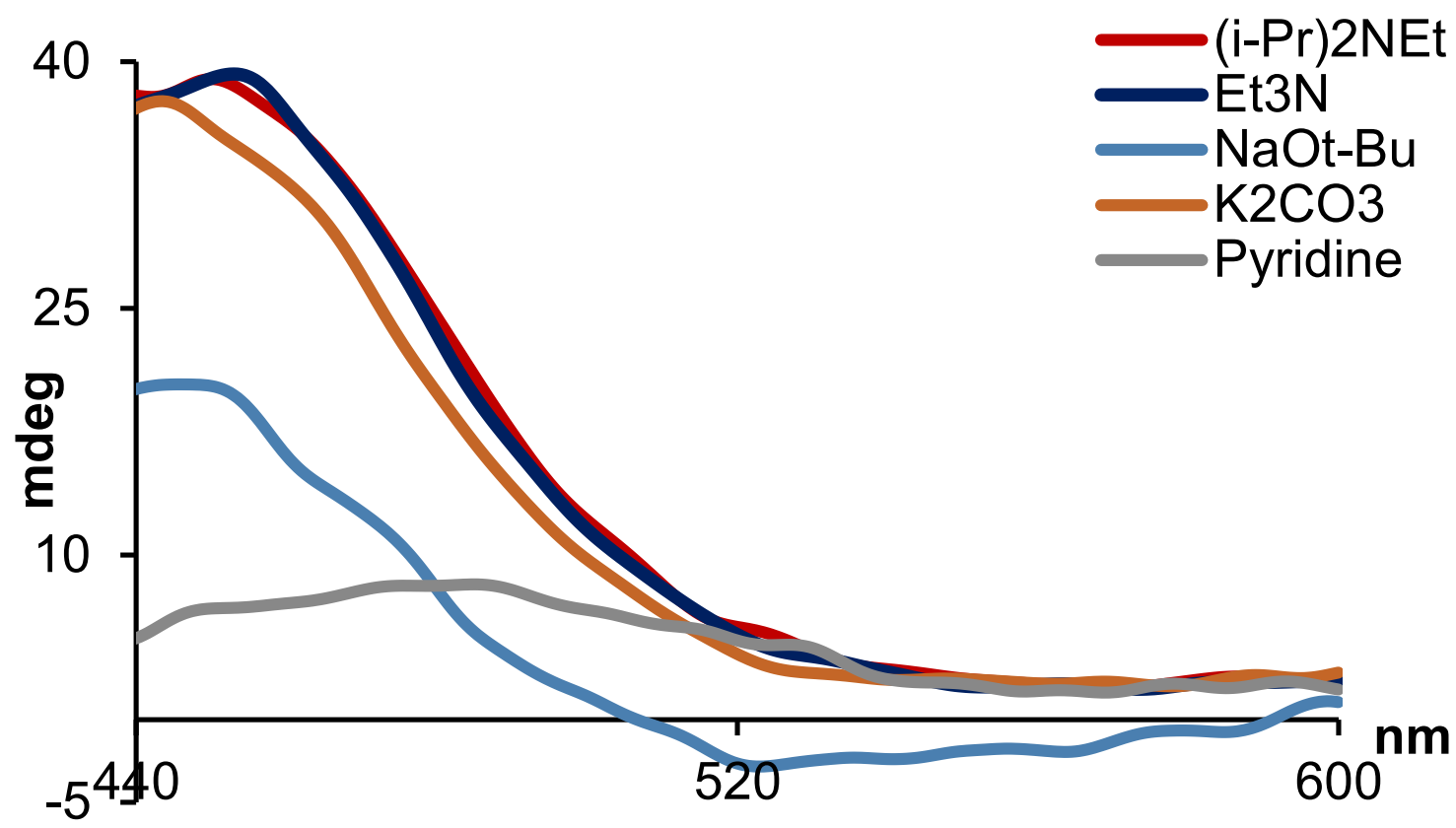

CD measurements were taken at 5.0 mM in CH_2Cl_2.

In a glass vial, 2.0 mL of a 5.0 mM sensor **1** stock solution in either acetone, acetonitrile, chloroform, 1,2-dichloroethane, or dichloromethane were mixed with (*S*)-ibuprofen (4.13 mg, 20.0 µmol) and 40.0 µL of a 0.50 M (*i*-Pr)$_2$NEt stock solution in dichloromethane. The mixtures were stirred for 18 hours under nitrogen.

Scheme S3. Solvent effect on the CD response of sensor **1** to acid (*S*)-**3.**

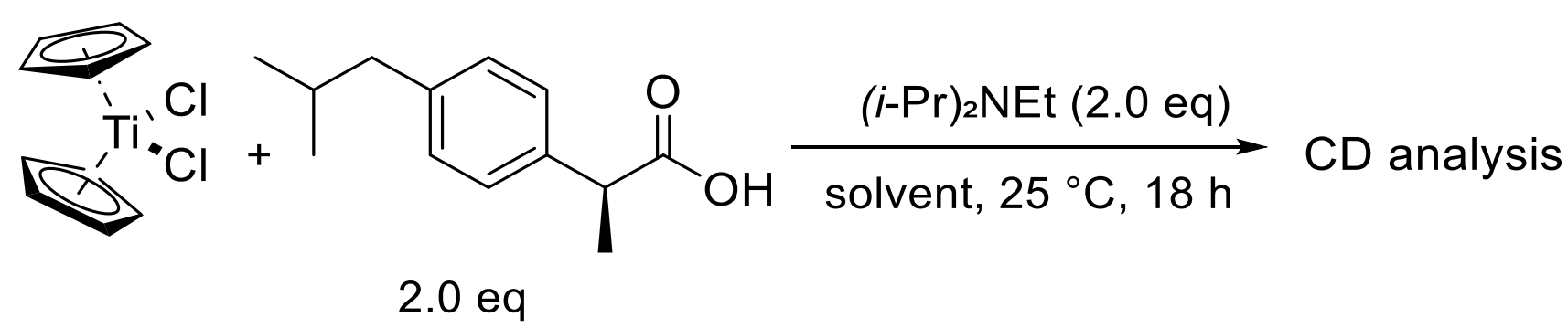

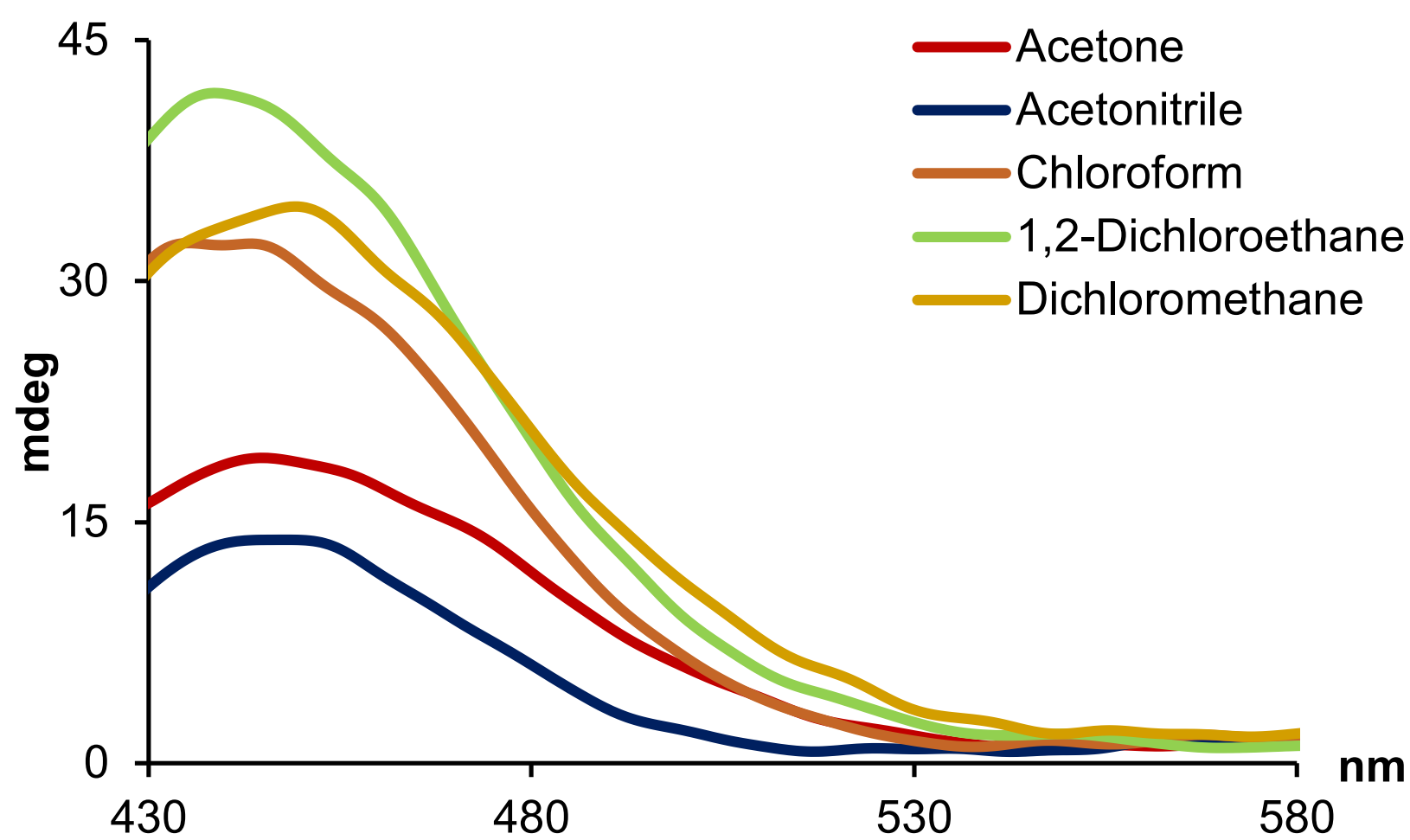

CD Measurements were taken at 5.0 mM.

In a glass vial, 0.40 mL of a 50.0 mM (*S*)-ibuprofen solution in $CDCl_3$, 44.0 µL of a 0.50 M (*i*-Pr)$_2$NEt solution in $CDCl_3$, and 1.6 mL of a 6.25 mM sensor **1** stock solution in $CDCl_3$ were sequentially combined under nitrogen. The resulting mixture was analyzed by ^{1}H NMR after 1, 2 and 3 hours.

Scheme S4. ^{1}H NMR analysis of the reaction between (*S*)-ibuprofen and sensor **1**.

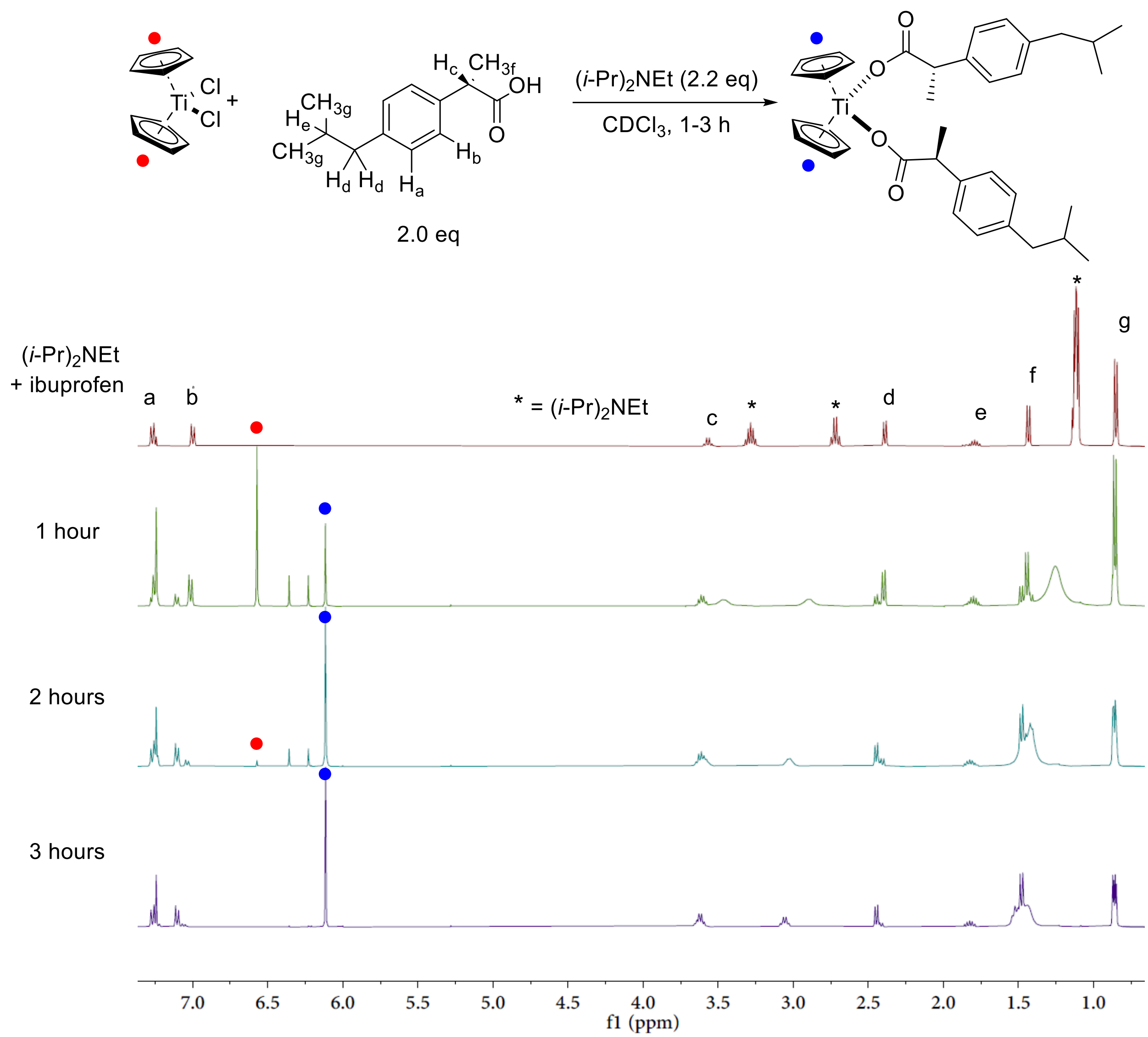

3. Carboxylic acid sensing scope of sensor 1

To 0.20 mL of a 0.10 M stock solution of the acid in 1,2-dichloroethane (DCE) was added 44.0 µL of a 0.50 M (i-Pr)$_2$NEt stock solution in DCE. Then, 1.6 mL of a 6.25 mM sensor **1** stock solution in DCE and 0.156 mL of DCE were added to each vial. The mixtures were stirred for 3 hours at 25 °C and CD analysis was performed directly with the reaction mixtures without further dilution.

Scheme S5. General procedure for carboxylic acid screening with sensor **1**.

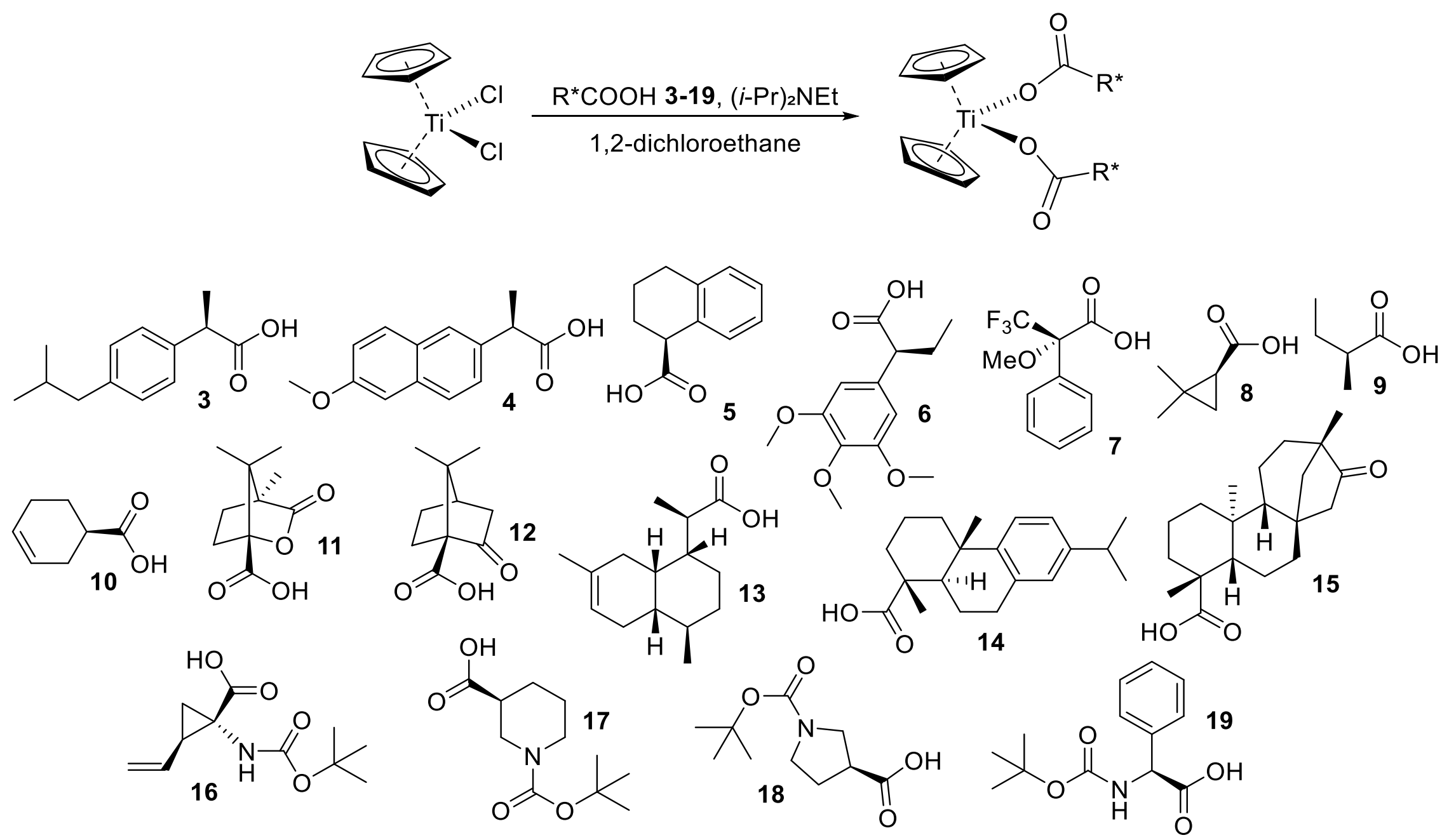

Figure S2. CD spectra obtained by applying sensor **1** to (*R*)-**3** (red) vs (*S*)-**3** (blue).

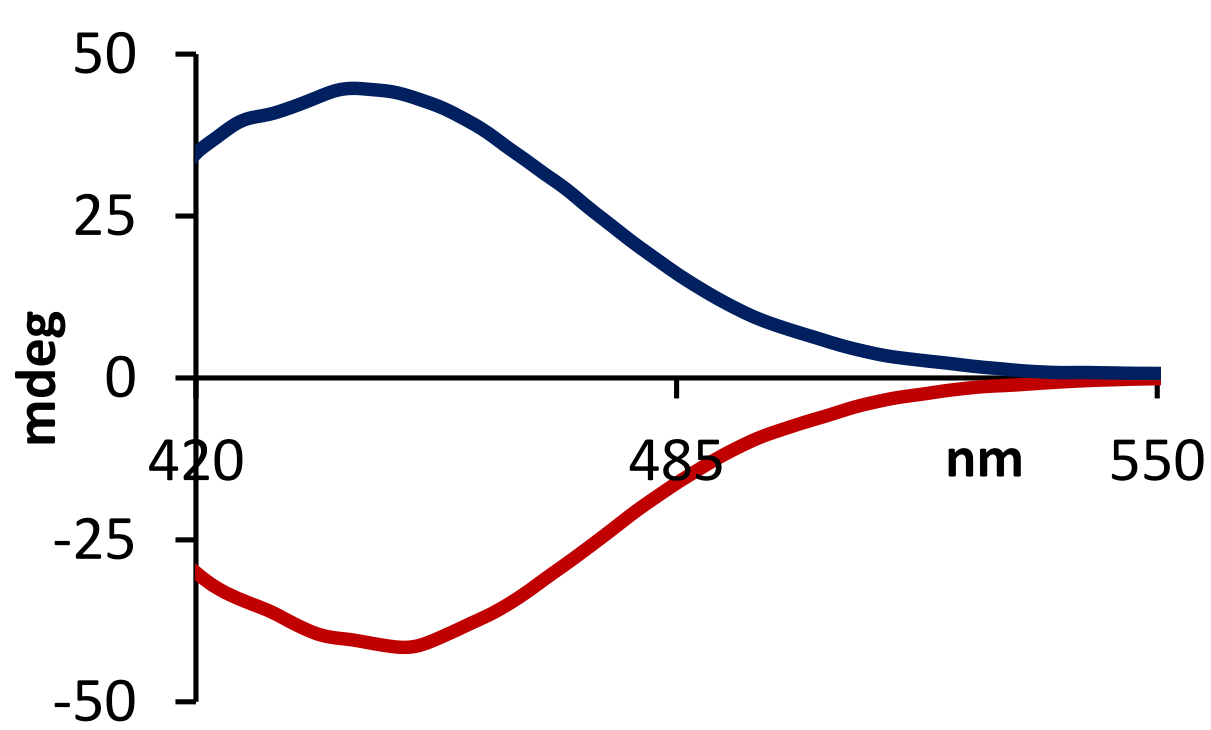

CD Measurements were taken at 5.0 mM.

Figure S3. CD spectra obtained by applying sensor **1** to (*S*)-**4**.

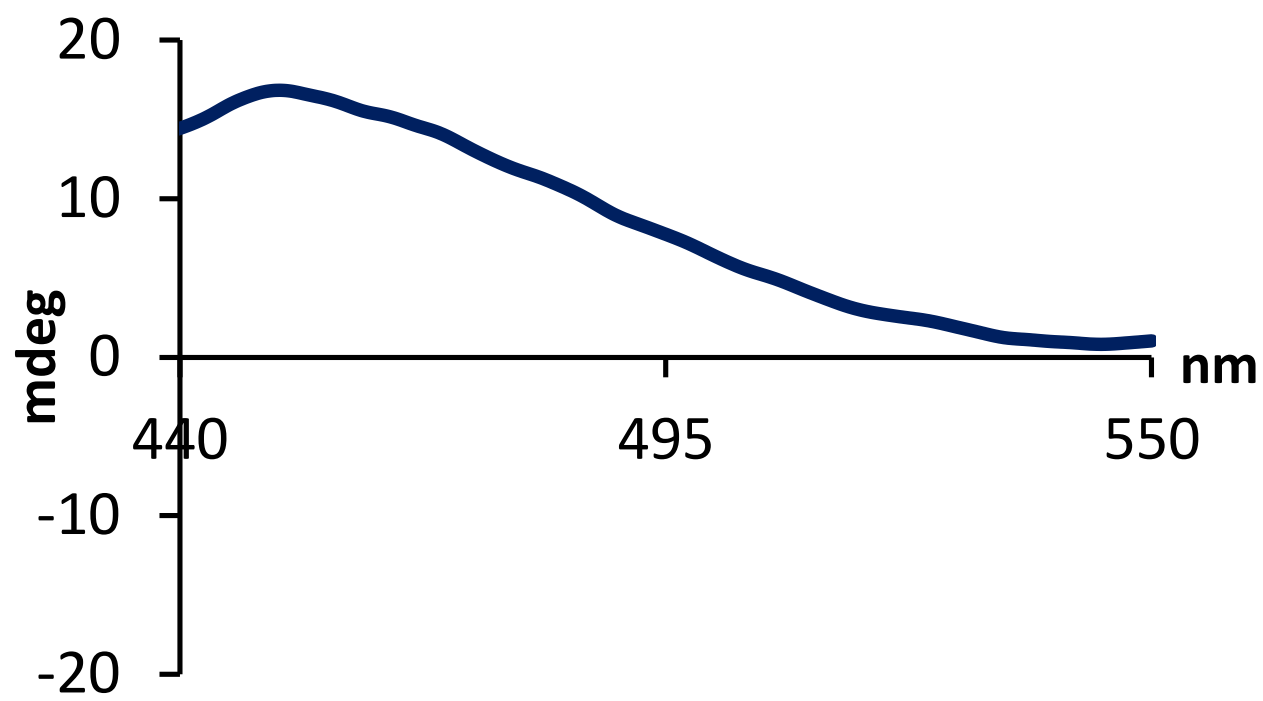

CD Measurement was taken at 5.0 mM.

Figure S4. CD spectra obtained by applying sensor **1** to (*R*)-**5** (red) vs (*S*)-**5** (blue).

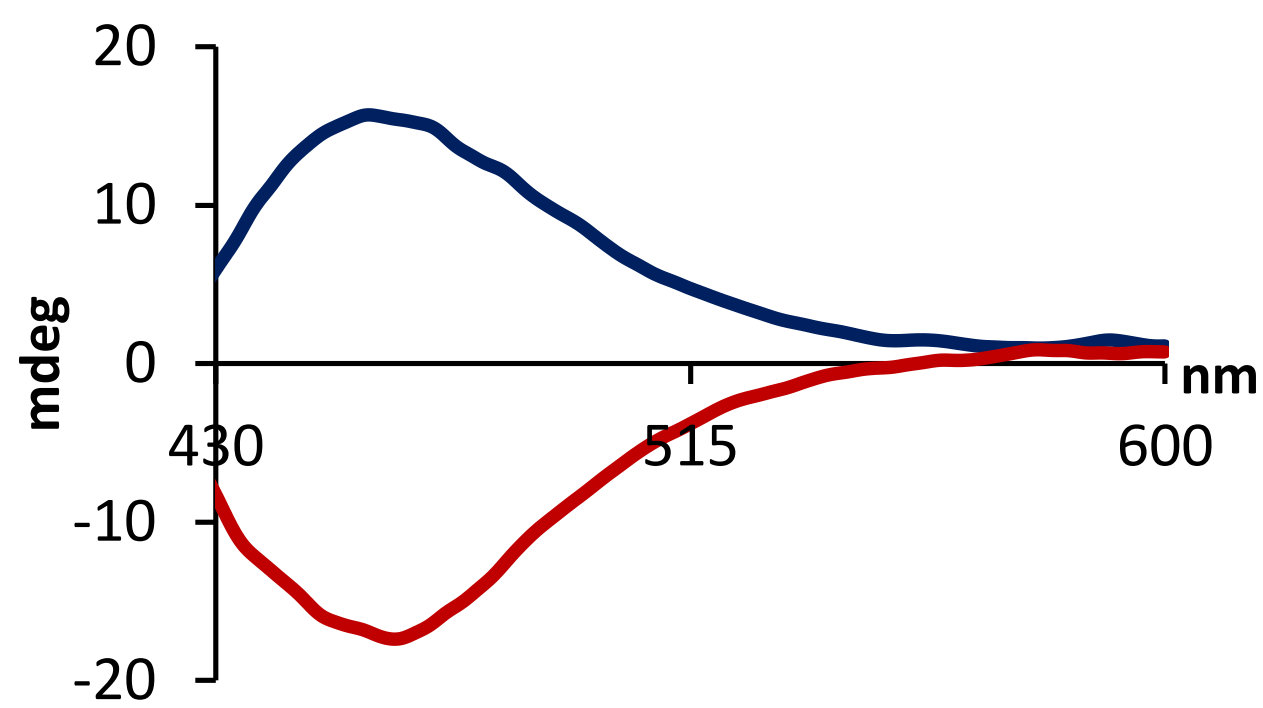

CD Measurements were taken at 5.0 mM.

Figure S5. CD spectra obtained by applying sensor **1** to (*S*)-**6**.

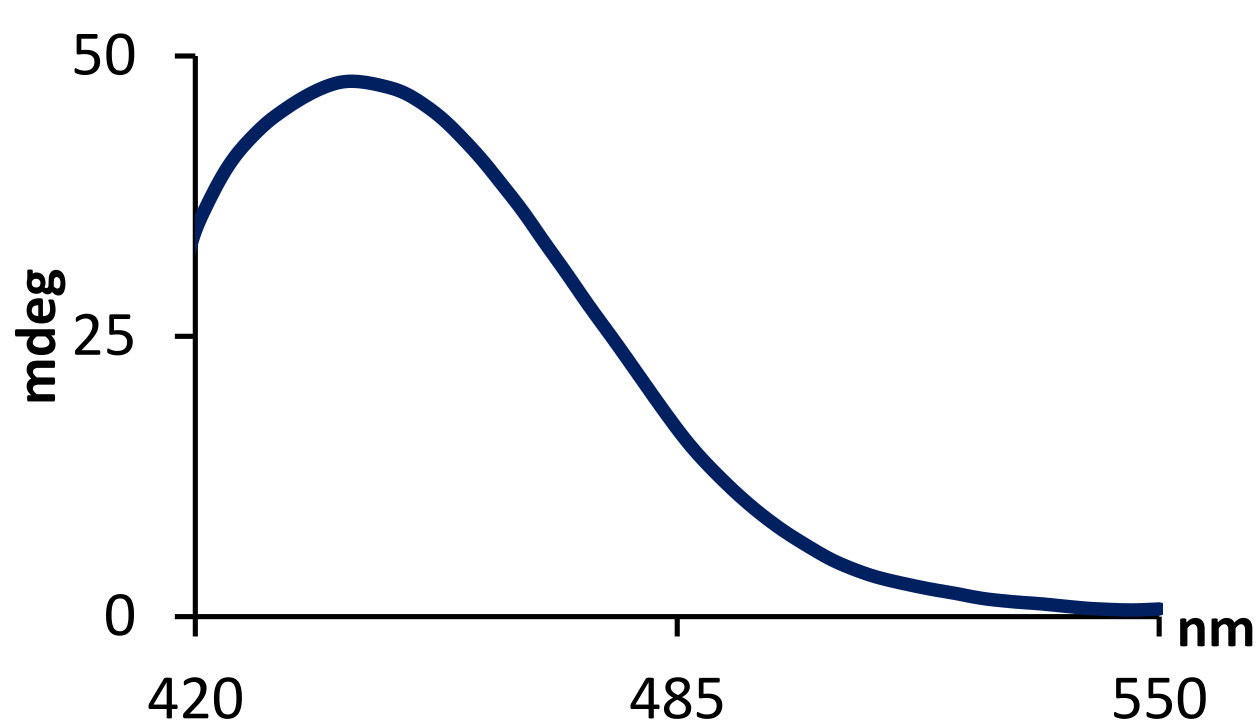

CD Measurement was taken at 5.0 mM.

Figure S6. CD spectra obtained by applying sensor **1** to (*R*)-**7** (red) vs (*S*)-**7** (blue).

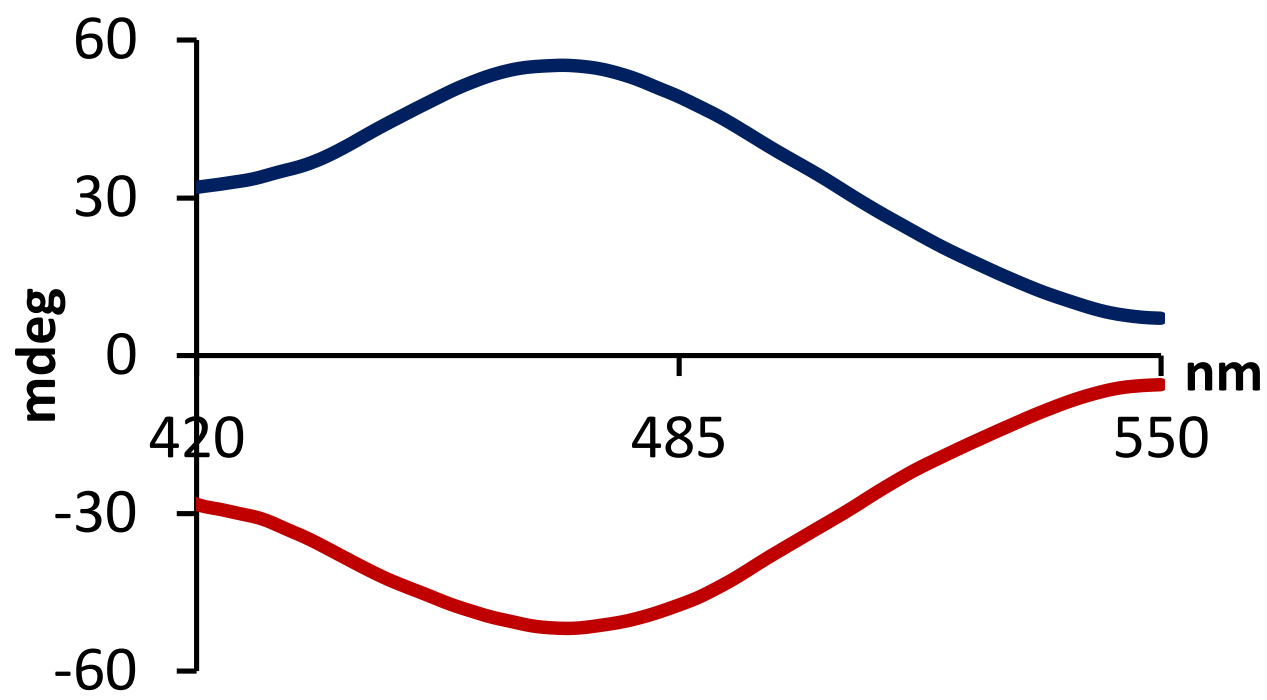

CD Measurements were taken at 5.0 mM.

Figure S7. CD spectra obtained by applying sensor **1** to (*S*)-**8**.

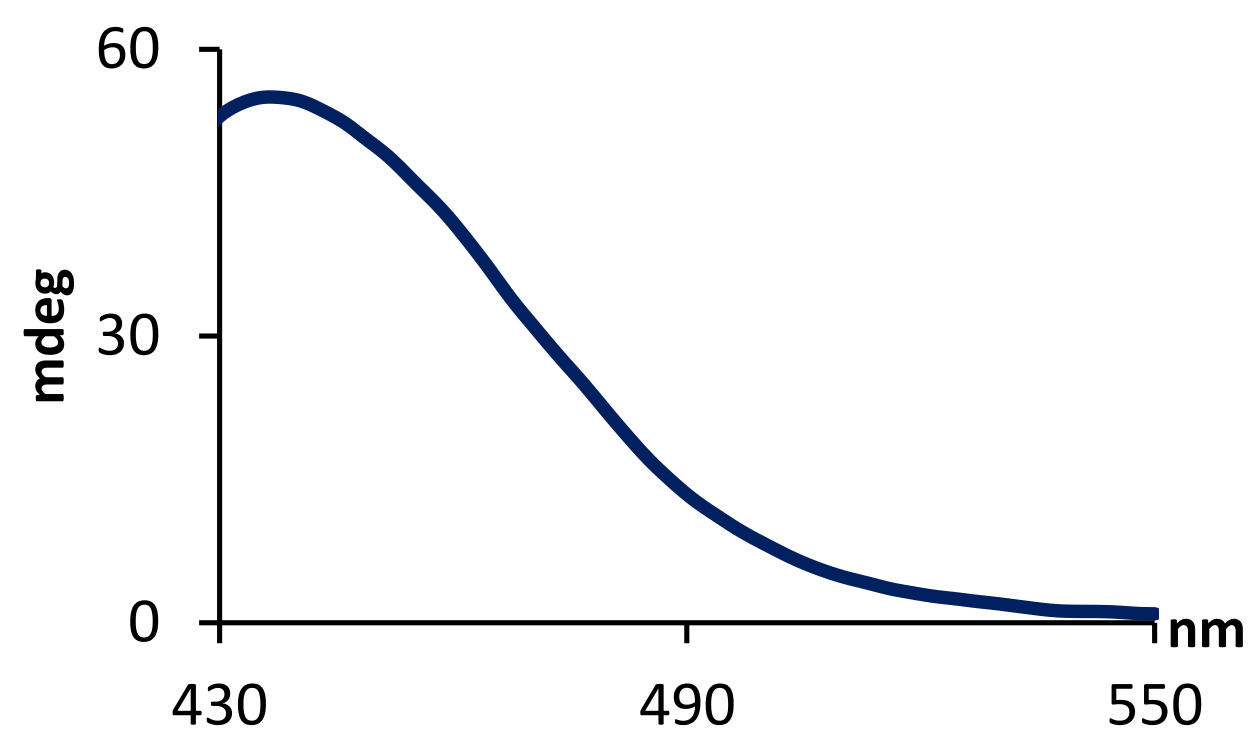

CD Measurement was taken at 5.0 mM.

Figure S8. CD spectra obtained by applying sensor **1** to (*S*)-**9**.

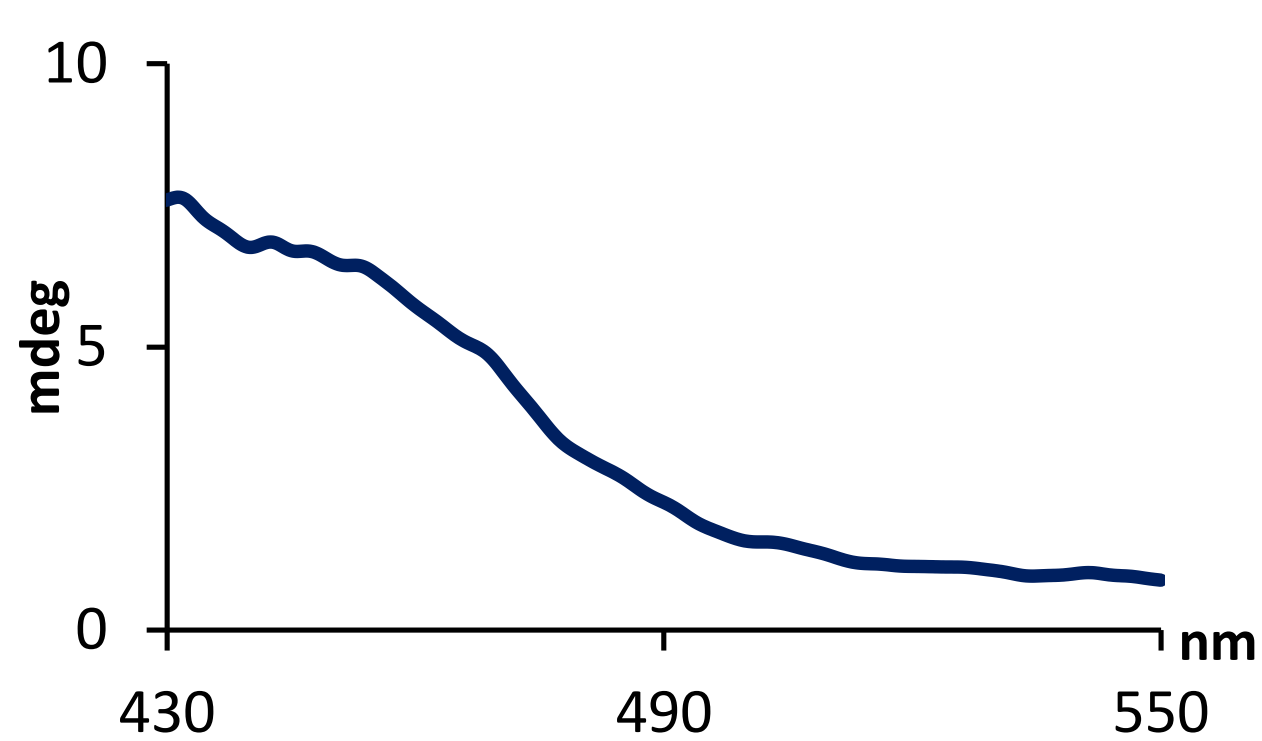

CD Measurement was taken at 5.0 mM.

Figure S9. CD spectra obtained by applying sensor **1** to (*S*)-**10**.

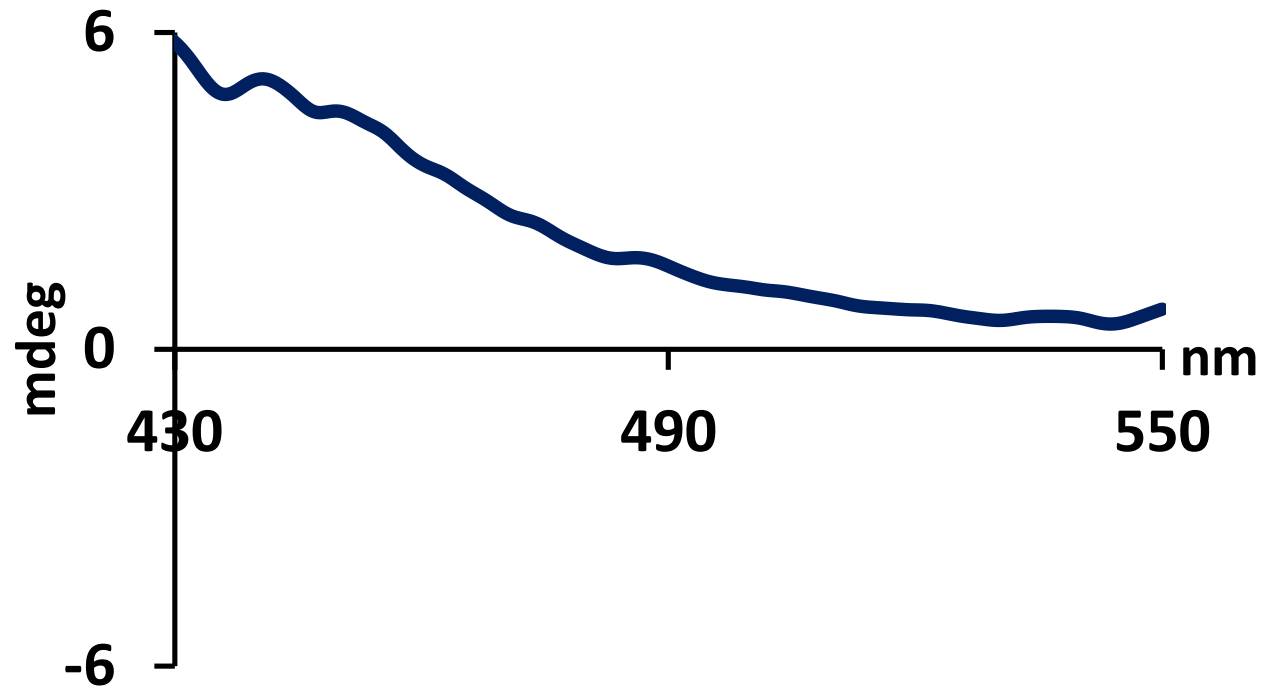

CD Measurement was taken at 5.0 mM.

Figure S10. CD spectra obtained by applying sensor **1** to (*1R,4S*)-**11** (red) vs (*1S,4R*)-**11** (blue).

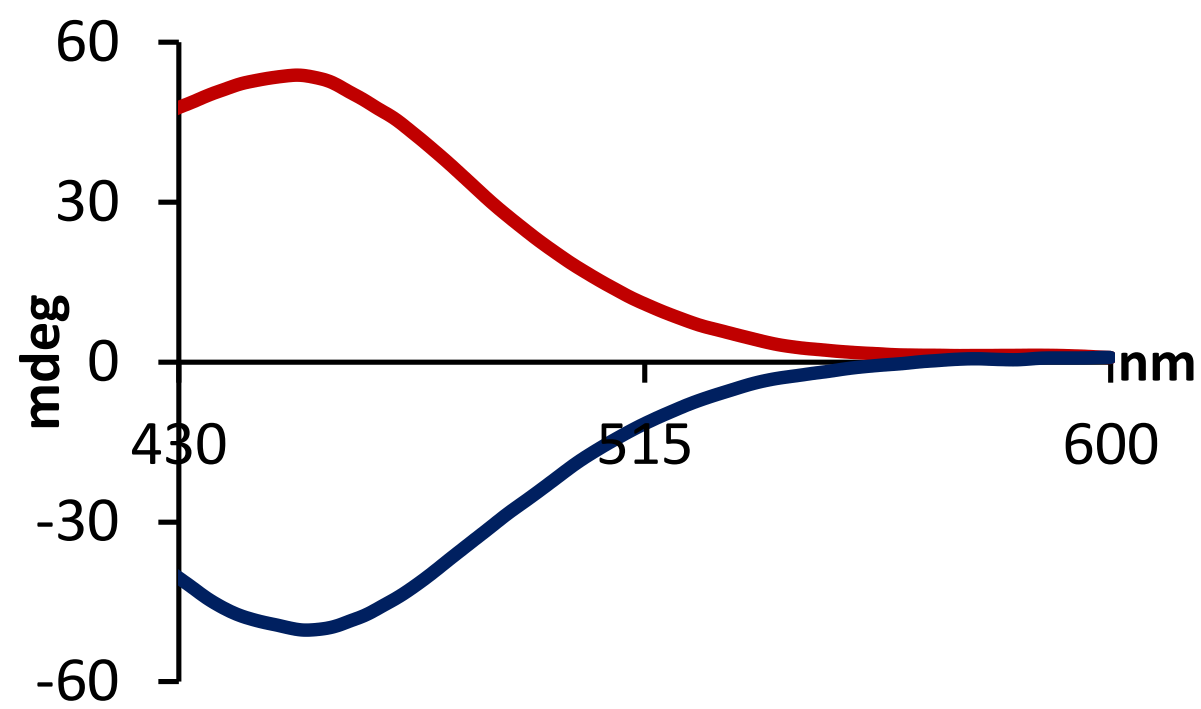

CD Measurements were taken at 5.0 mM.

Figure S11. CD spectra obtained by applying sensor **1** to (*S*)-**12**.

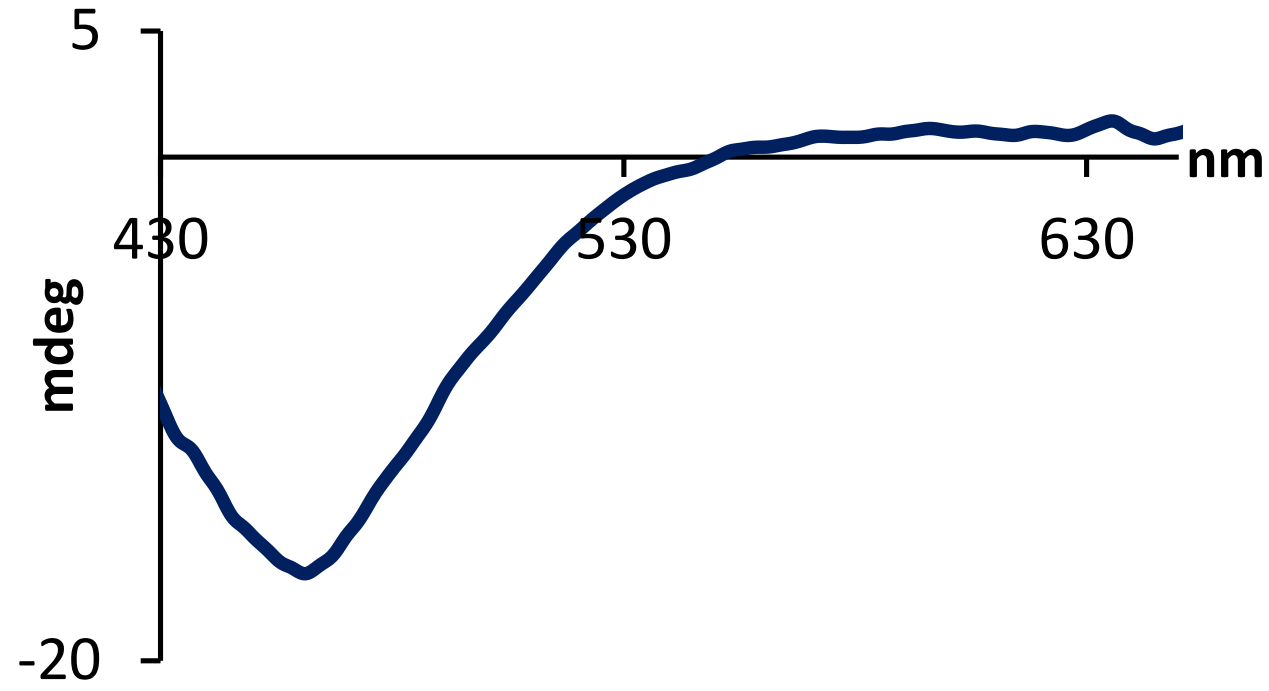

CD Measurement was taken at 5.0 mM.

Figure S12. CD spectra obtained by applying sensor **1** to acid **13** (dihydroartemisinic acid).

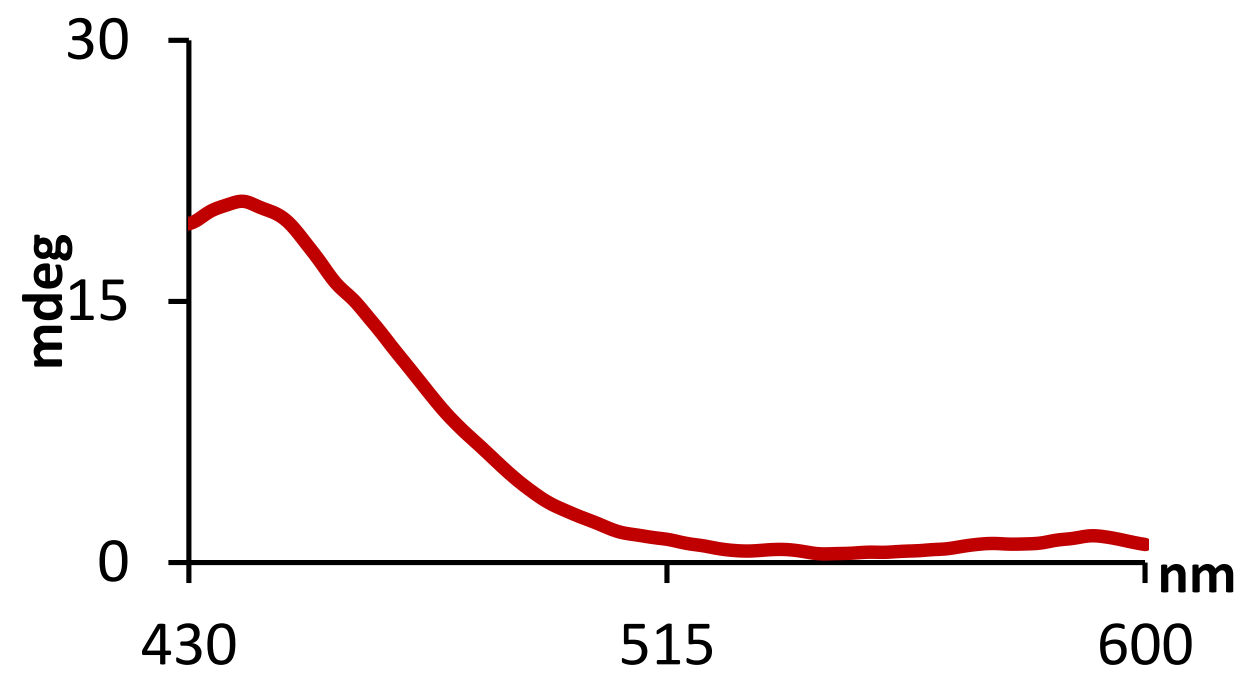

CD Measurement was taken at 5.0 mM.

Figure S13. CD spectra obtained by applying sensor **1** to acid **14** (dehydroabietic acid).

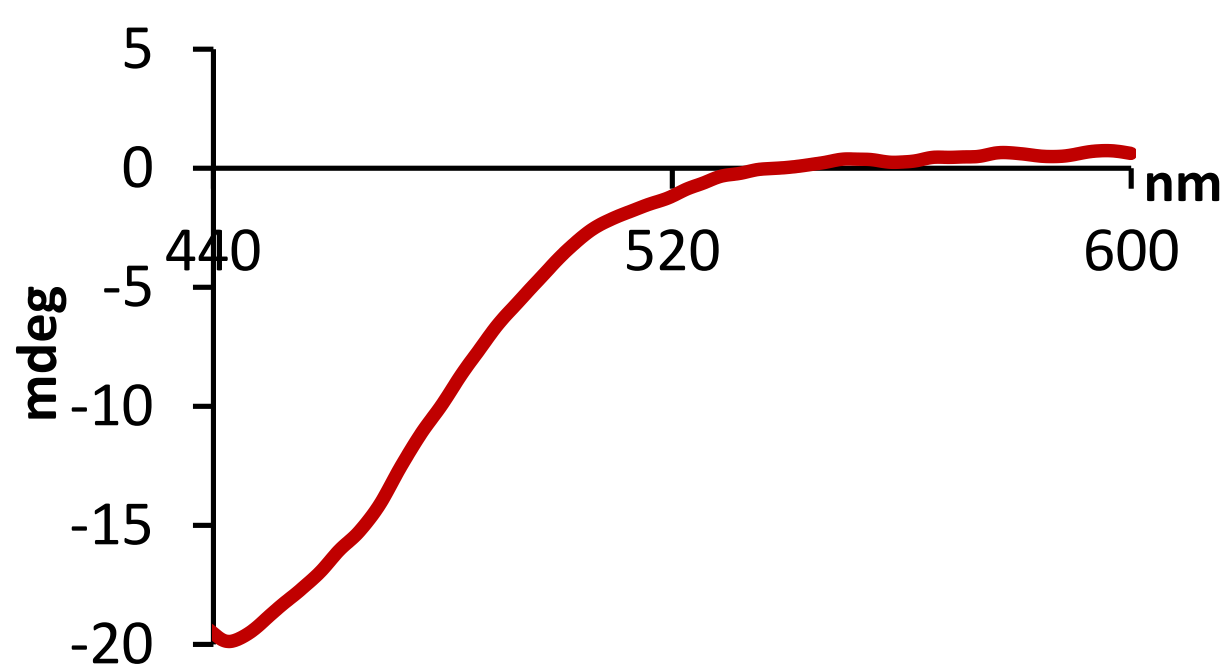

CD Measurement was taken at 5.0 mM.

Figure S14. CD spectra obtained by applying sensor **1** to acid **15** (isosteviol).

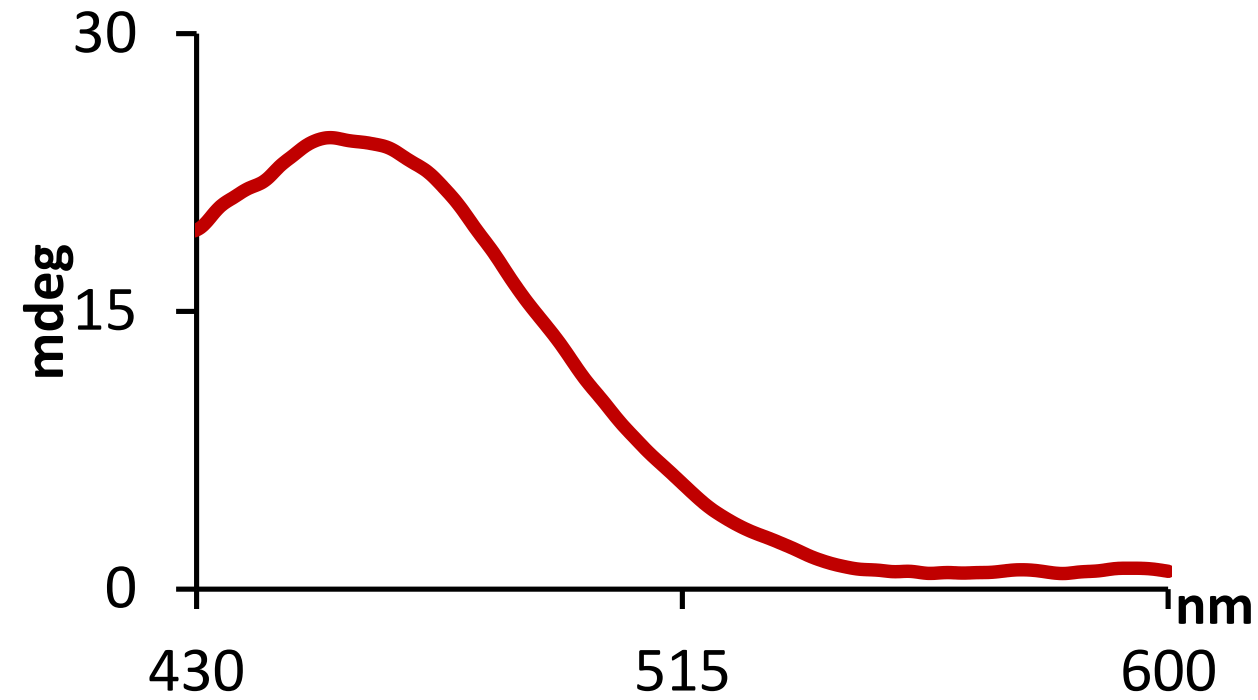

CD Measurement was taken at 5.0 mM.

Figure S15. CD spectra obtained by applying sensor **1** to (1*R*,2*S*)-**16** (red) vs (1*S*,2*R*)-**16** (blue).

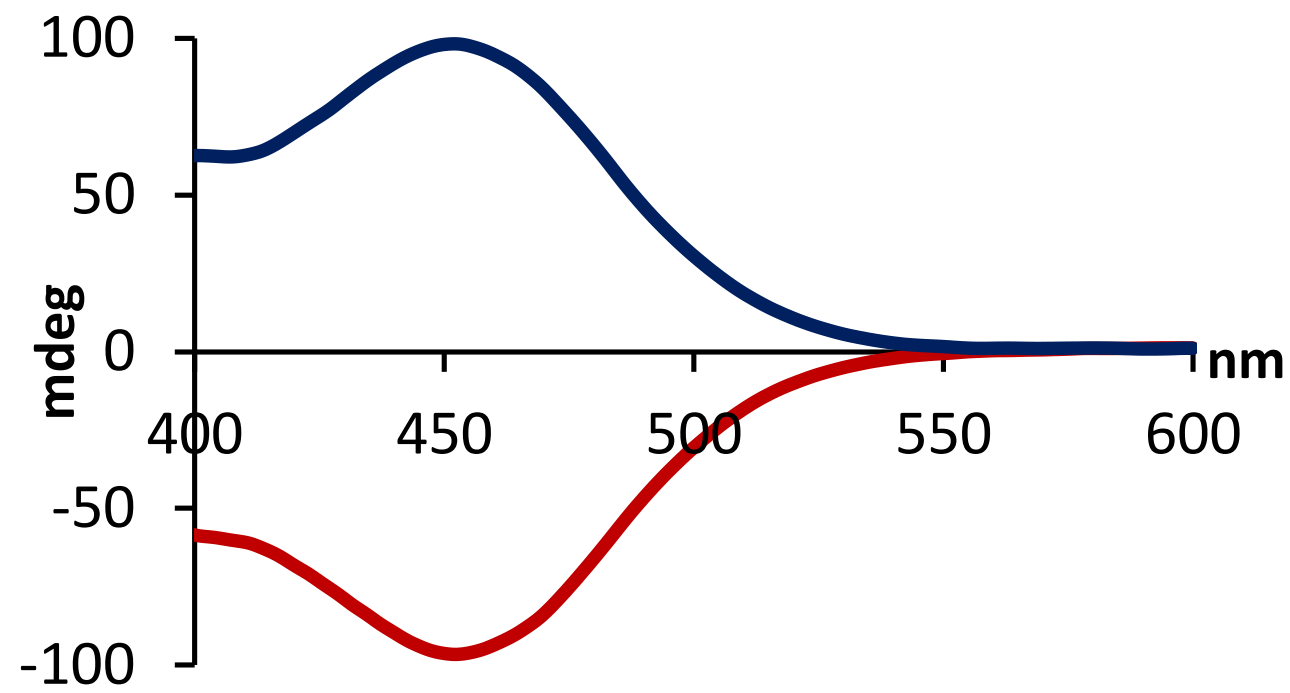

CD Measurements were taken at 5.0 mM.

Figure S16. CD spectra obtained by applying sensor **1** to (*R*)-**17** (red) vs (*S*)-**17** (blue).

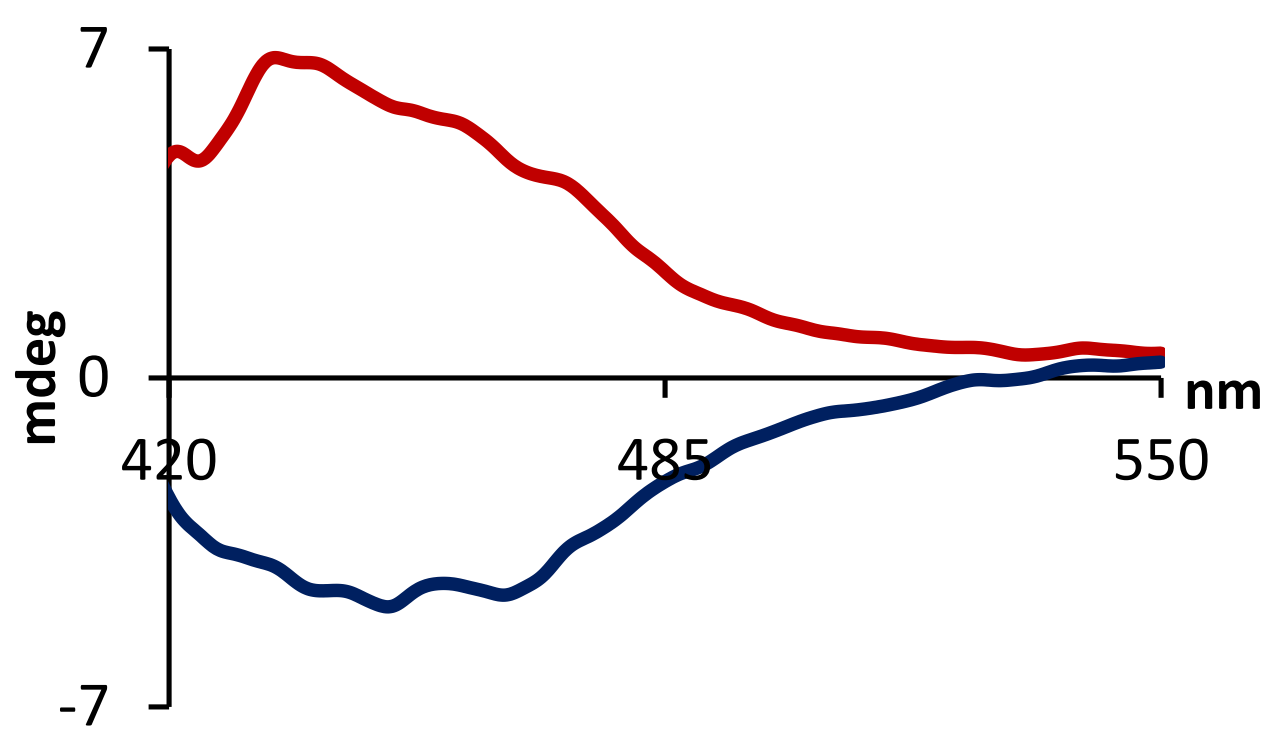

CD Measurements were taken at 5.0 mM.

Figure S17. CD spectra obtained by applying sensor **1** to (*S*)-**18**.

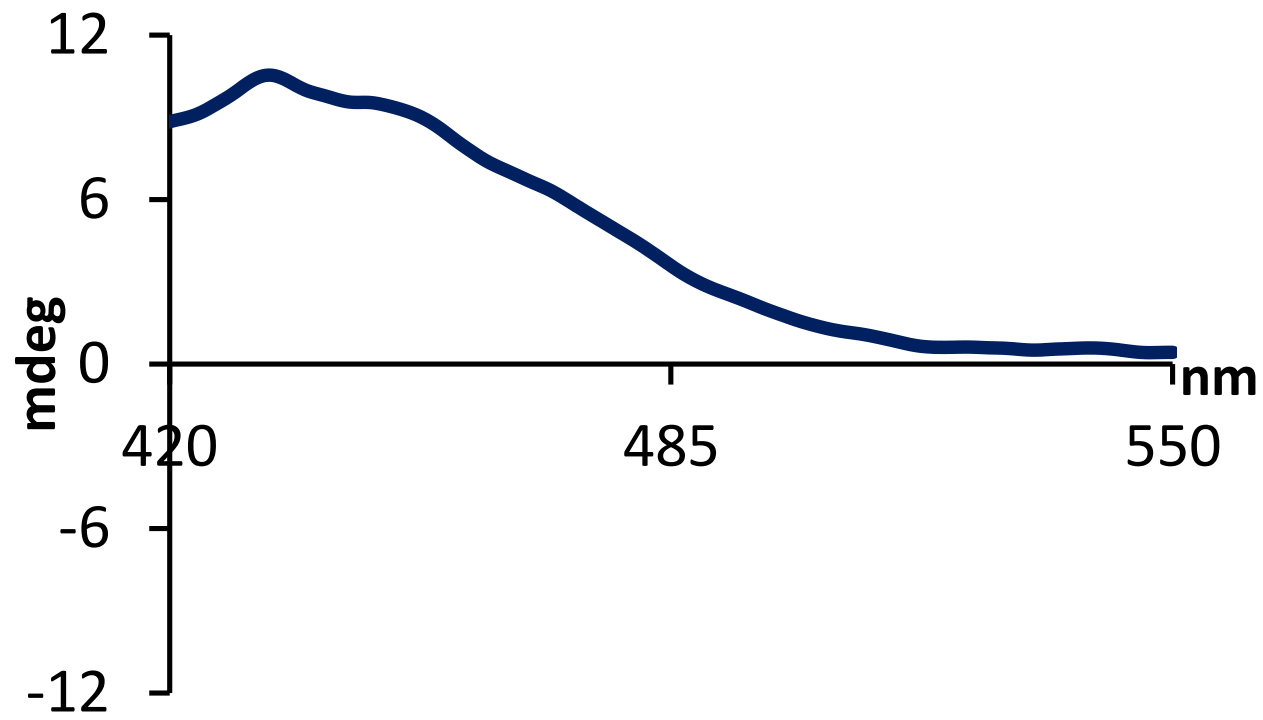

CD Measurement was taken at 5.0 mM.

4. Carboxylic acid *er* sensing with sensor 1

Samples containing enantioenriched acid **19** were analyzed. To 1.6 mL of a 6.25 mM sensor **1** stock solution in 1,2-dichloroethane (DCE) were added 0.22 mL of a 0.10 M $(i\text{-}Pr)_2NEt$ stock solution and 0.22 mL of a 0.10 M stock solution of acid **19** in DCE with varying *ee*'s (-100, -80, -60, -40, -20, 0, +20, +40, +60, +80, and +100%). The mixtures were stirred for 3 hours under nitrogen. CD analysis was conducted directly with the reaction mixtures without further dilution. The CD amplitude at 456 nm was plotted against the enantiomeric excess of acid **19**, yielding a linear correlation ($R^2 = 0.9994$) described by the equation: $y = 0.8194x – 0.4405$.

Scheme S6. CD response of sensor **1** to scalemic samples of acid **19**.

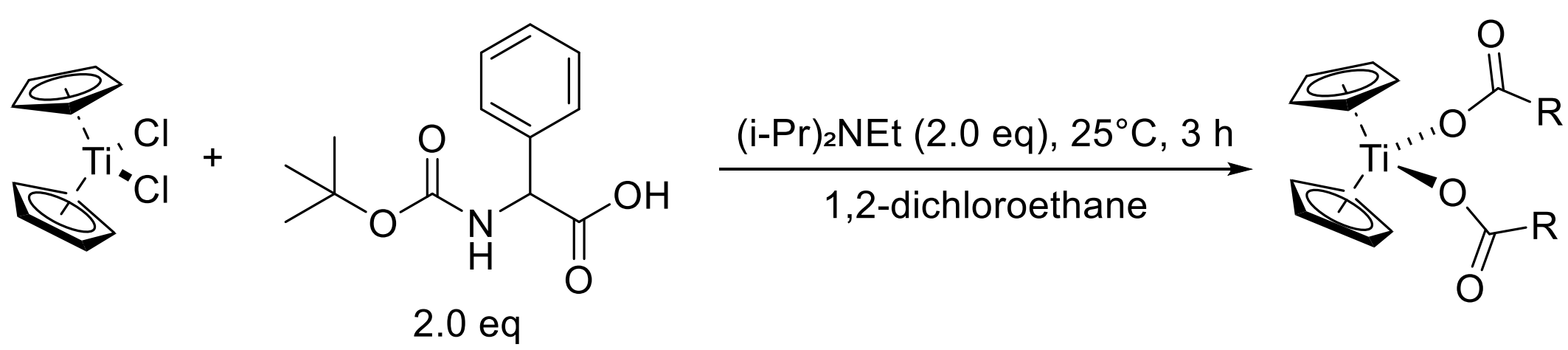

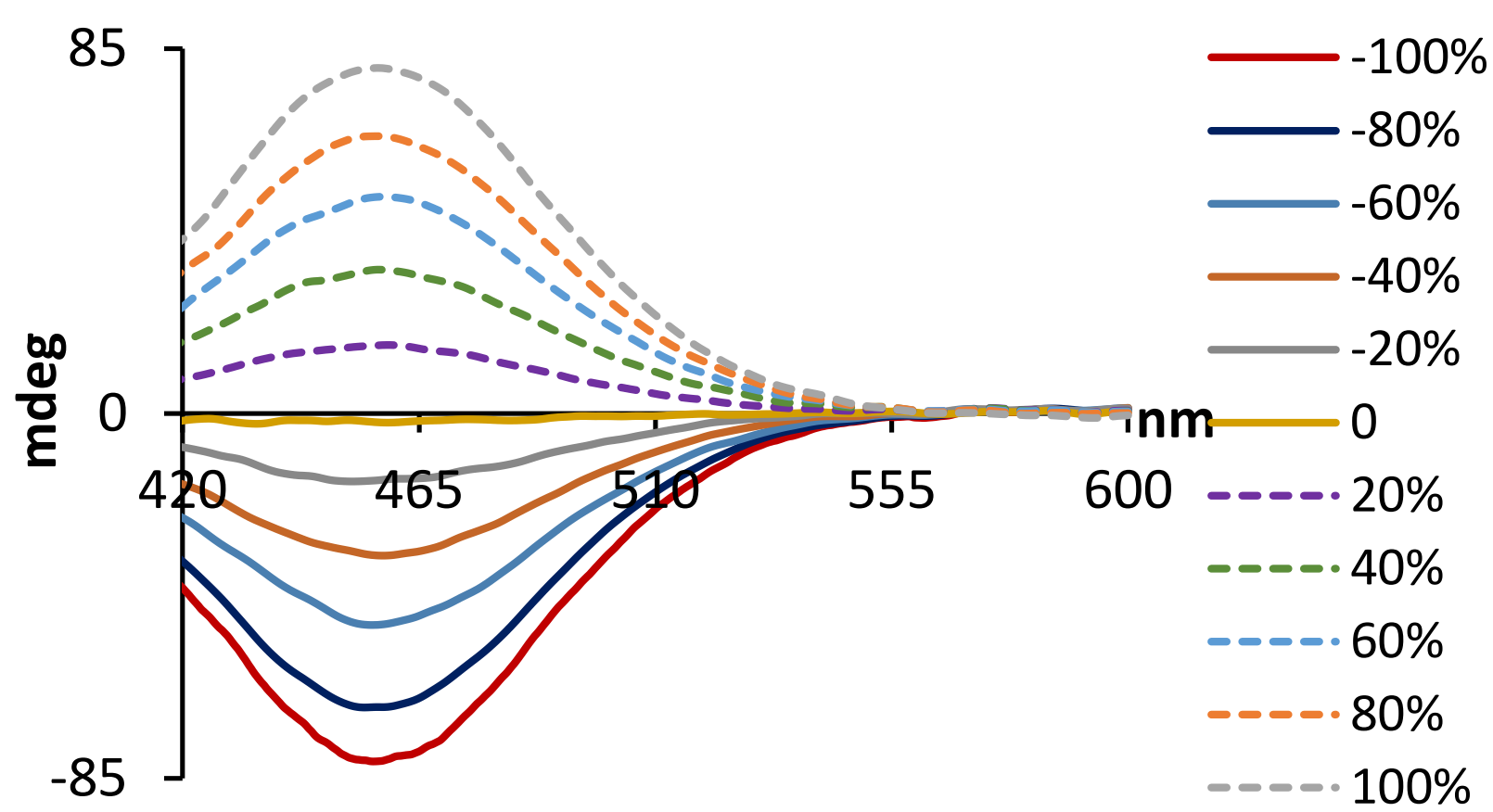

Figure S18. Induced CD amplitude at 456 nm vs. sample *ee*. All CD measurements were taken at 5.0 mM.

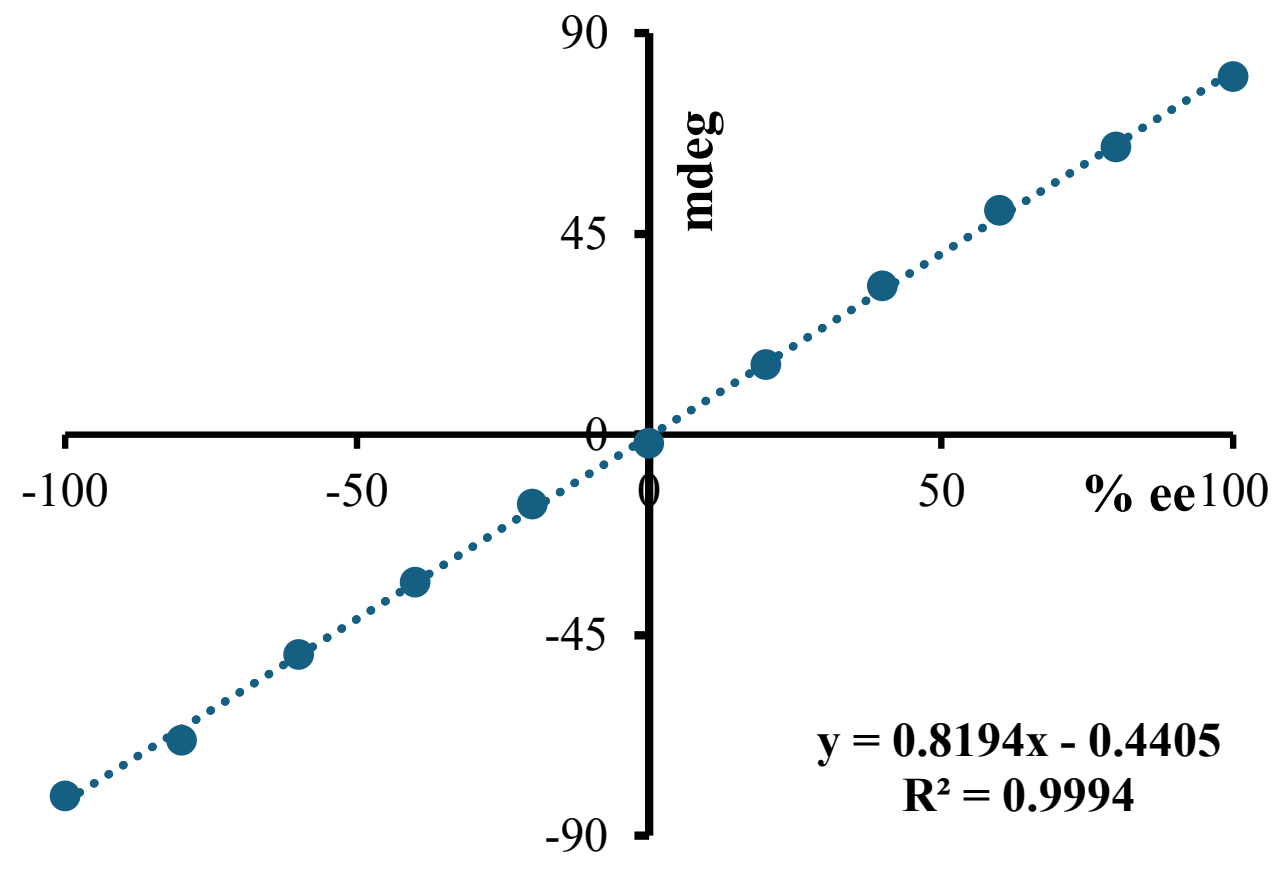

Enantiomeric ratio determination

Nine scalemic samples of acid **19,** each at a concentration of 10.0 mM and varying *er* were prepared in 1,2-dichloroethane. The samples were subjected to CD analysis as described above and the enantiomeric ratios were determined using the CD intensities measured at 465 nm and the calibration equation.

Table S1. Absolute configuration and enantiomeric ratio of samples of acid **19** analyzed with sensor **1**.

Sample	Sample composition		Sensing results	
	Abs. Config.	*er*	Abs. Config.	*er*
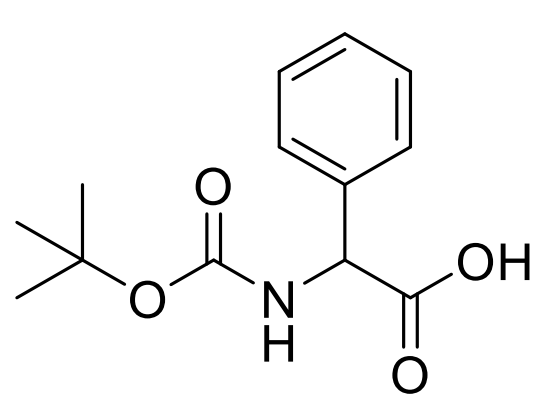	*S*	93.7:6.3	*S*	93.6:6.4
	S	80.0:20.0	*S*	82.2:17.8
	S	63.7:36.3	*S*	65.0:35.0
	S	57.5:42.5	*S*	57.8:42.2
	R	38.8:61.2	*R*	39.7:60.3
	R	32.5:67.5	*R*	32.0:68.0
	R	25.0:75.0	*R*	24.6:75.4
	R	16.2:83.8	*R*	15.5:84.5
	R	8.7:91.3	*R*	7.4:92.6

5. Optimization of sensing conditions for sensor 2

In a glass vial, 1.6 mL of a 6.25 mM sensor **2** stock solution in *N,N*-dimethylformamide (DMF) were mixed with either 0.20 mL or 0.40 mL of a 50.0 mM (*S*)-ibuprofen stock solution in DMF, and 2.2 equivalents of K_2CO_3. For the 0.20 mL (*S*)-ibuprofen sample, an additional 0.20 mL of DMF was added. The mixtures were stirred for 18 hours under nitrogen. No significant difference was observed in the CD spectra.

Scheme S7. Effect of acid equivalence on the ICD with sensor **2**.

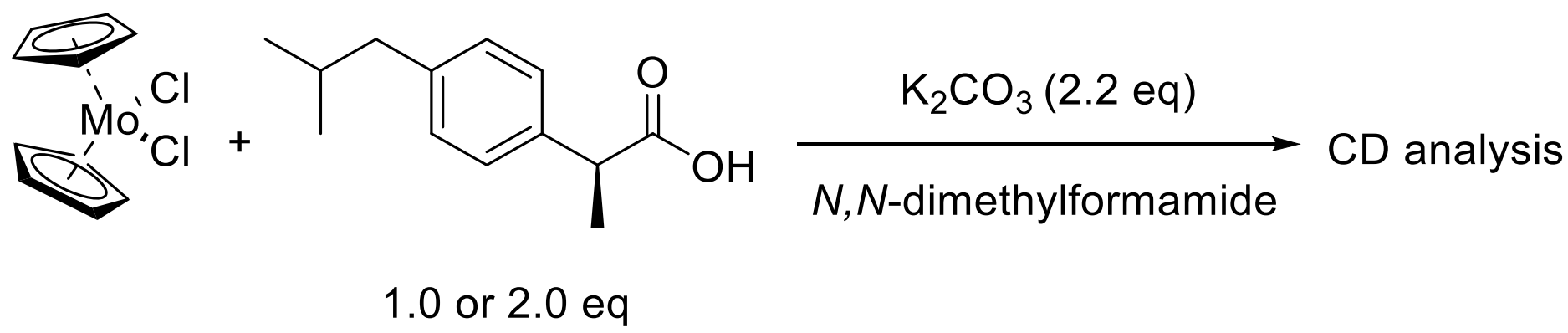

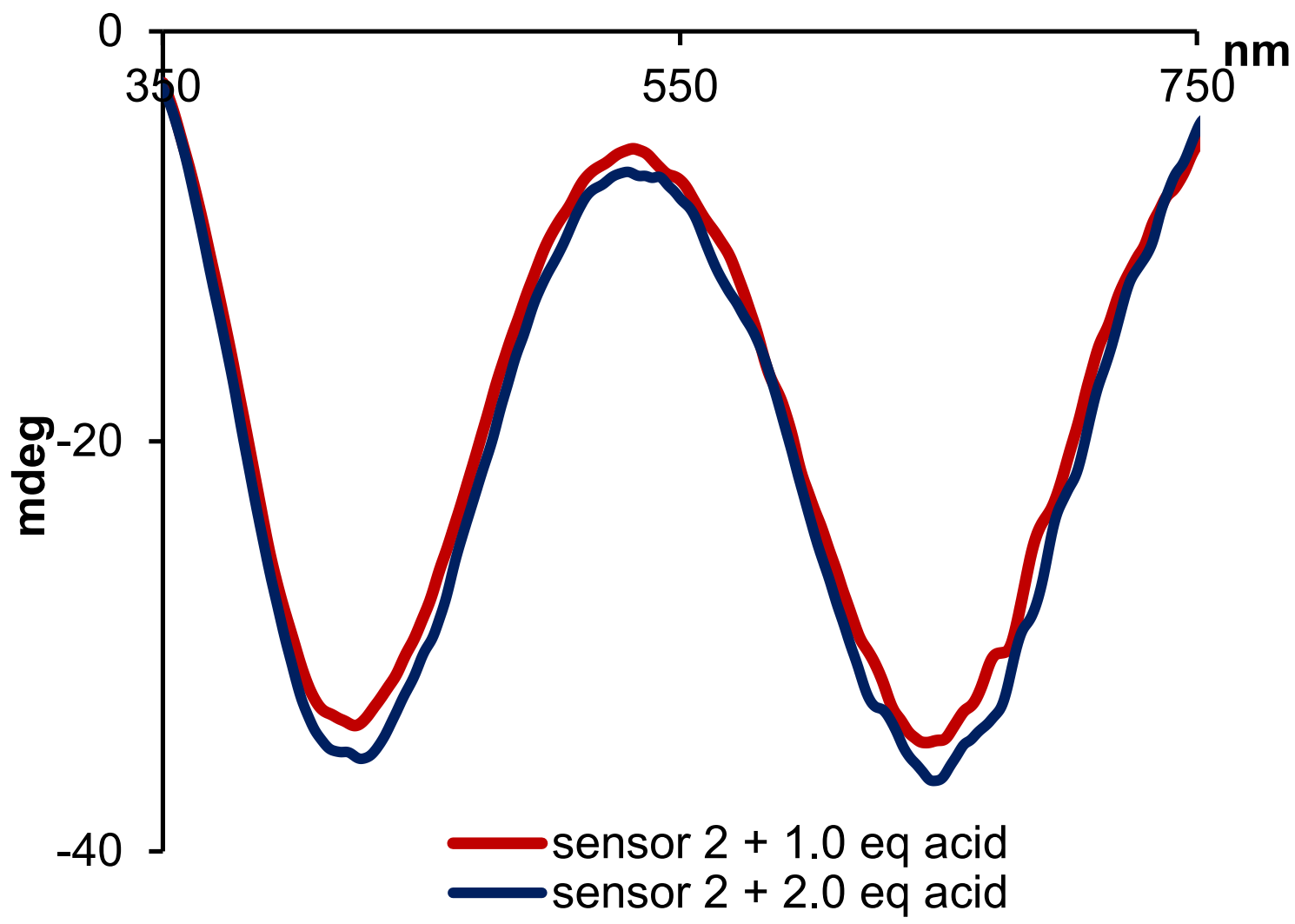

CD measurements were taken at 5.0 mM in *N,N*-dimethylformamide.

In a glass vial, 1.0 mL of a 10.0 mM sensor **2** stock solution in *N,N*-dimethylformamide (DMF) was mixed with 1.0 mL of a 20.0 mM (*S*)-ibuprofen stock solution in DMF. To this mixture, 40.0 µL of a 0.50 M stock solution in DMF of either (*i*-Pr)$_2$NEt or Et$_3$N were added. In parallel, DBU, K_2CO_3, or NaO*t*-Bu was added in amounts of 20.0 µmol each. The mixtures were stirred for 18 hours under nitrogen. The strongest ICD signal was observed when K_2CO_3 was used as the base.

Scheme S8. Effect of base on the ICD effects with sensor **2**.

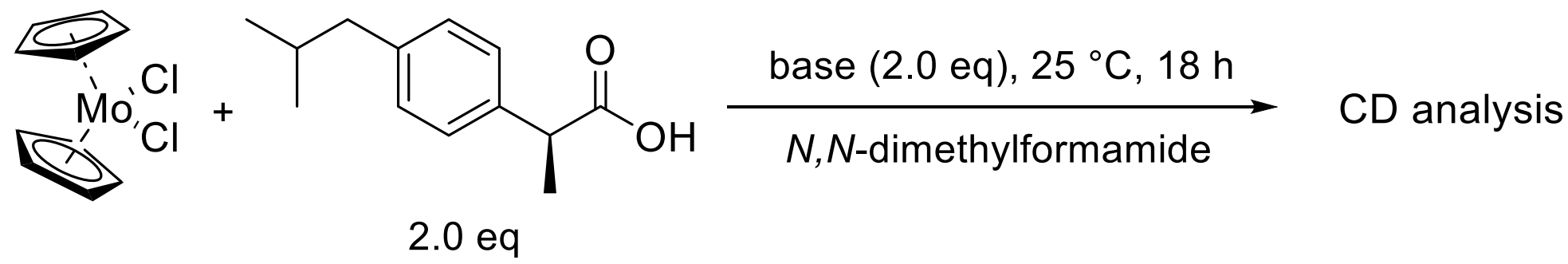

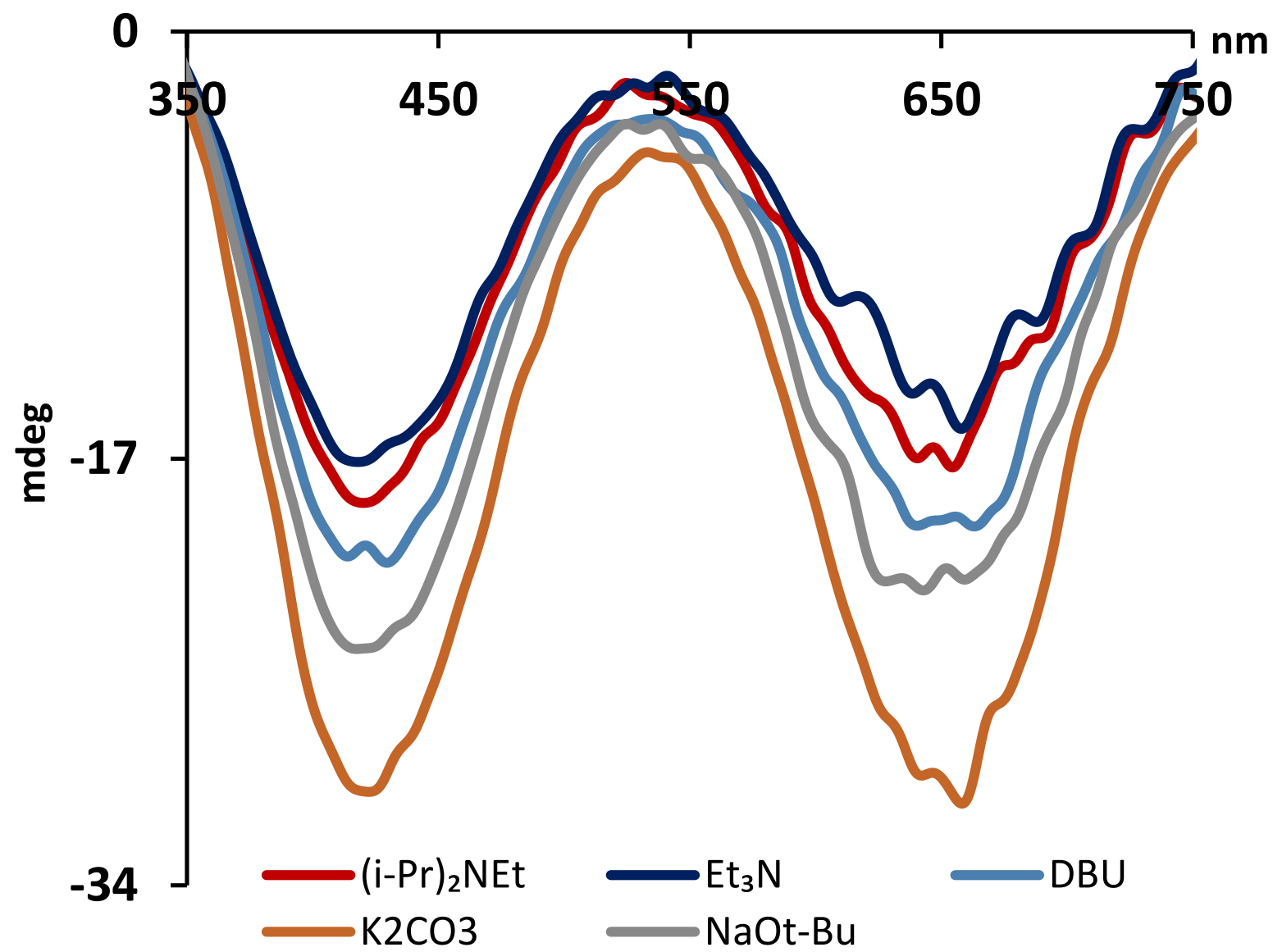

CD measurements were taken at 5.0 mM in *N,N*-dimethylformamide.

In a glass vial, 0.40 mL of a 25.0 mM (1*R*, 4*S*)-**11** stock solution in *N,N*-dimethylformamide (DMF), 1.1 equivalents of K_2CO_3, and 1.6mL of a 6.25 mM sensor **2** stock solution in DMF were sequentially combined under nitrogen. The resulting mixture was analyzed by CD after 1.5, 2.5 and 3.5 hours.

Scheme S9. CD spectra monitoring the progress of the reaction between sensor **2** and (1*R*,4*S*)-**11.**

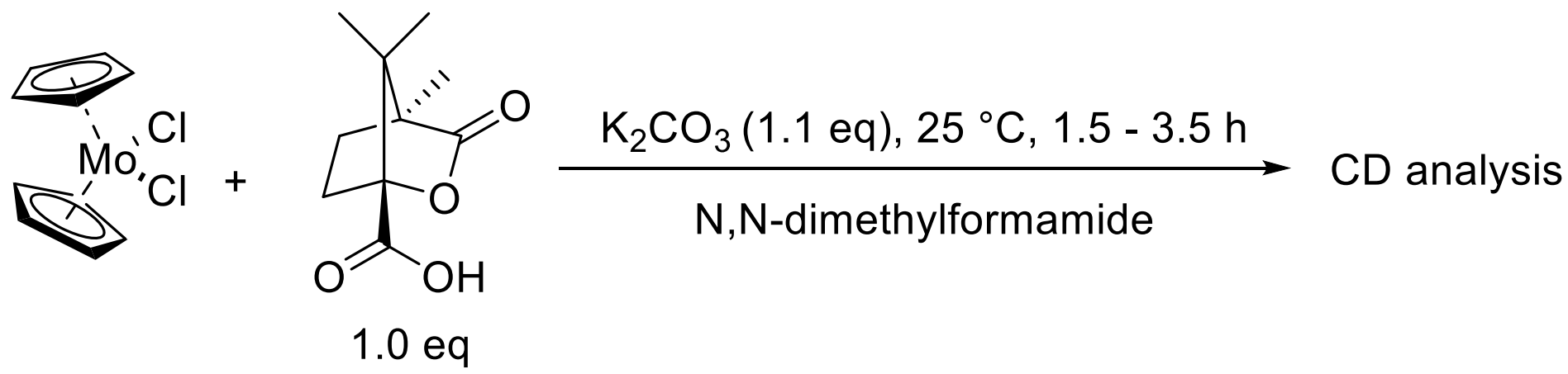

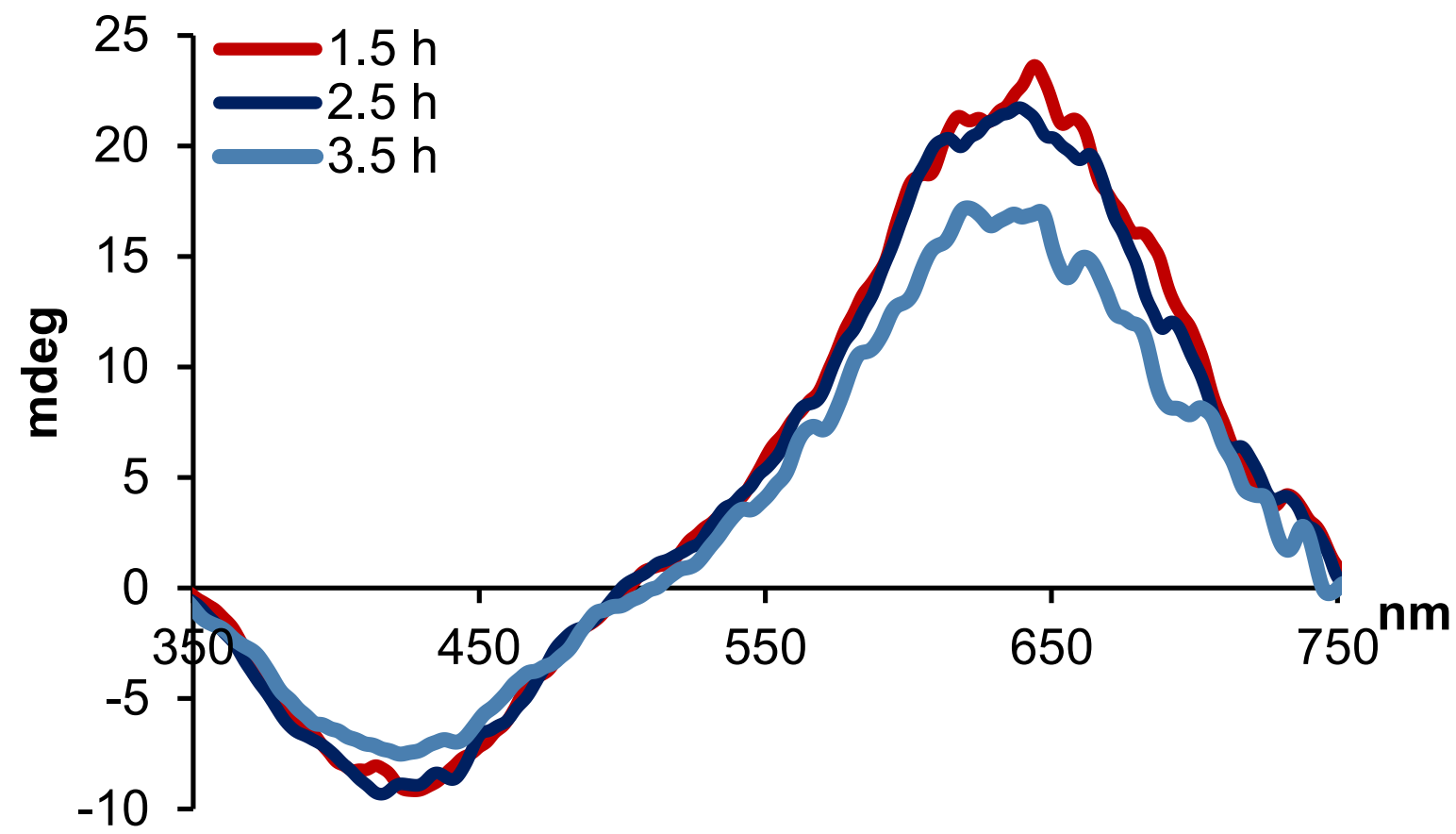

CD measurements were taken at 5.0 mM in *N,N*-dimethylformamide.

6. Carboxylic acid sensing scope of sensor 2

Into a glass vial, 0.40 mL of a 25.0 mM solution of the acid in *N,N*-dimethylformamide (DMF) was added, followed by 1.1 equivalents of K_2CO_3 as a solid. Then, 1.6 mL of a 6.25 mM sensor **2** stock solution in DMF was added to each vial. The mixtures were stirred for 1 hour at 25 °C, and CD analysis was performed without further dilution.

Scheme S10. General procedure for carboxylic acid screening with sensor **2**

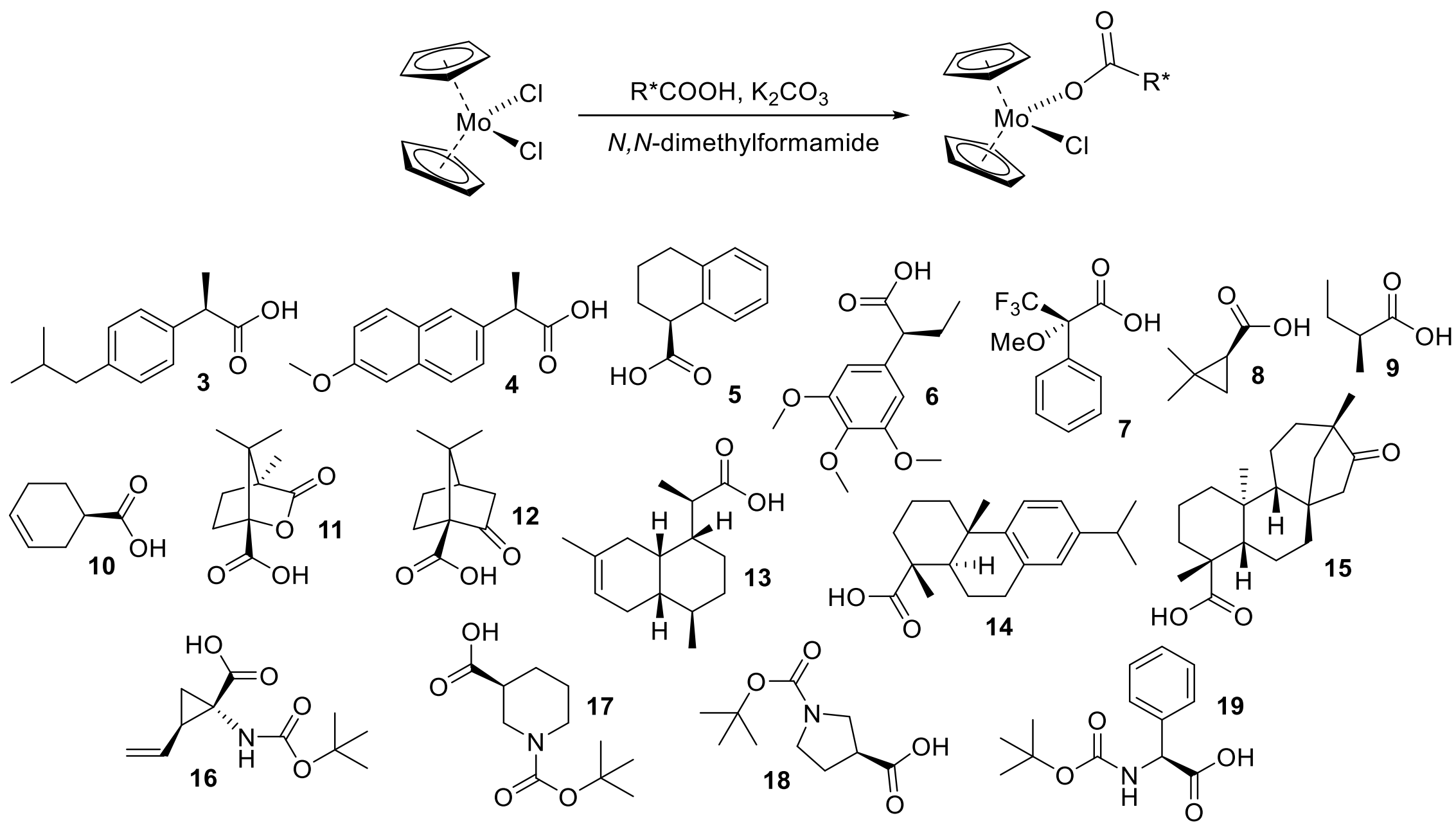

Figure S19. CD spectra obtained by applying sensor **2** to (*R*)-**3** (red) vs (*S*)-**3** (blue).

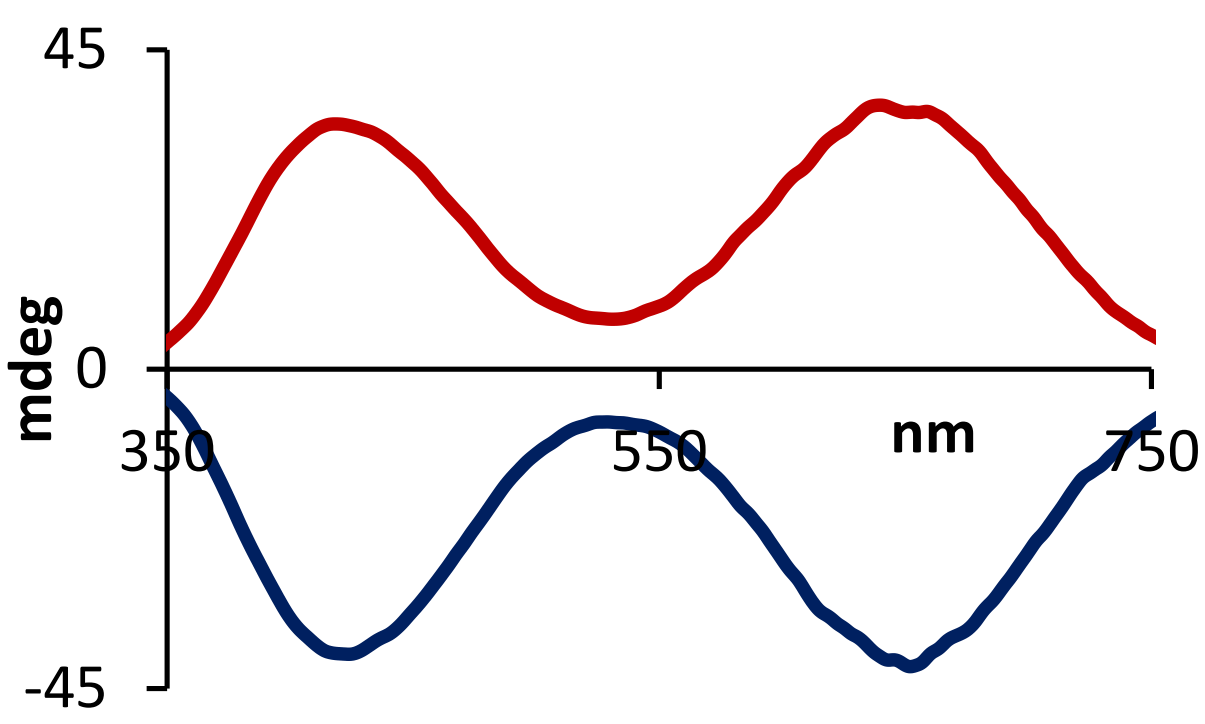

CD measurements were taken at 5.0 mM.

Figure S20. CD spectra obtained by applying sensor **2** to (*R*)-**4** (red) vs (*S*)-**4** (blue).

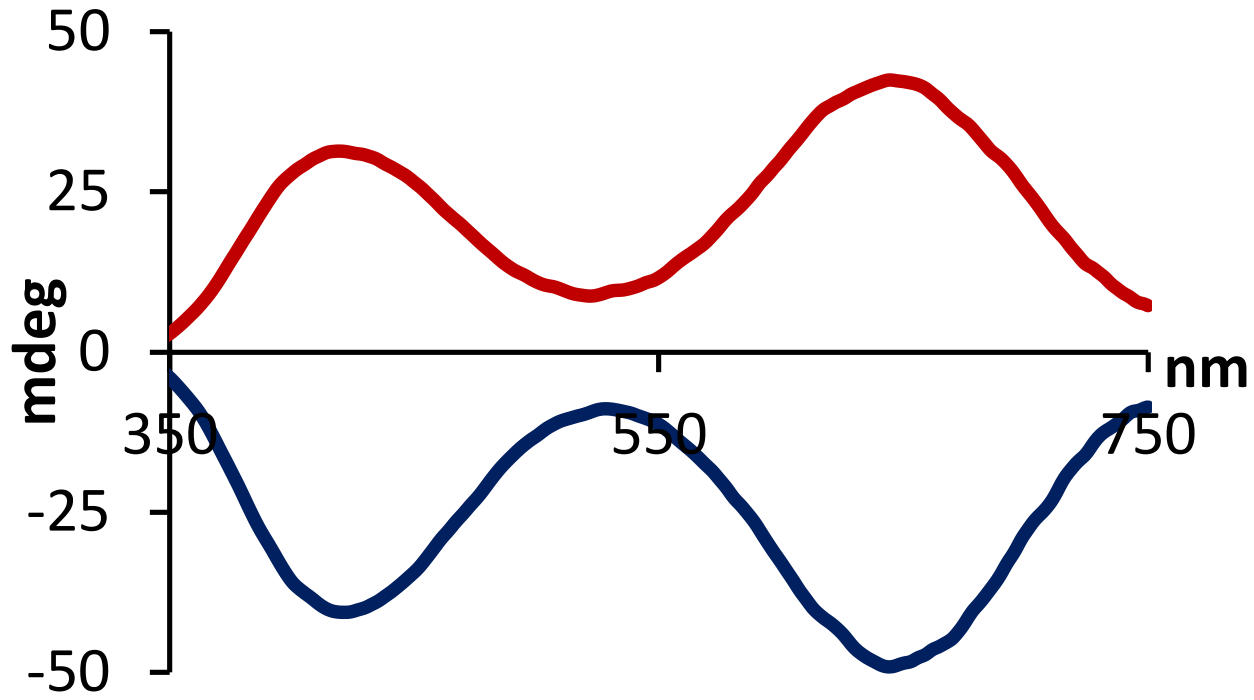

CD measurements were taken at 5.0 mM.

Figure S21. CD spectra obtained by applying sensor **2** to (*R*)-**5** (red) vs (*S*)-**5** (blue).

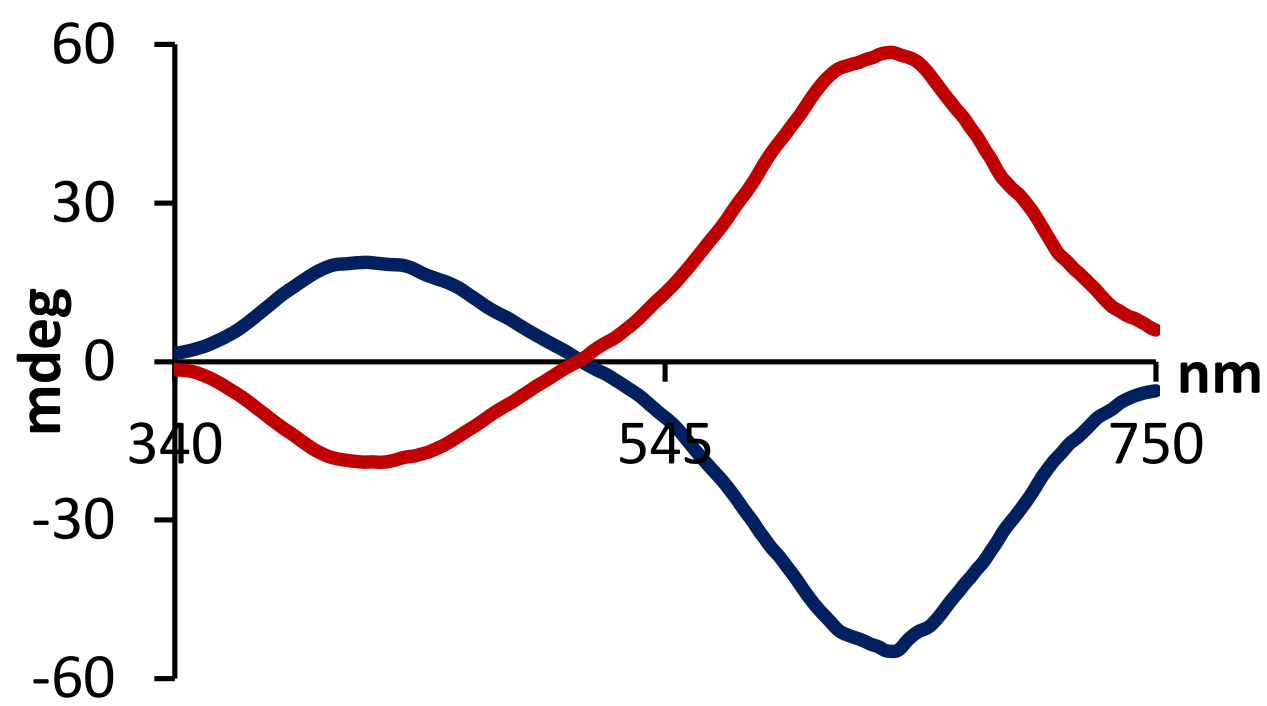

CD measurements were taken at 5.0 mM.

Figure S22. CD spectra obtained by applying sensor **2** to (*S*)-**6**.

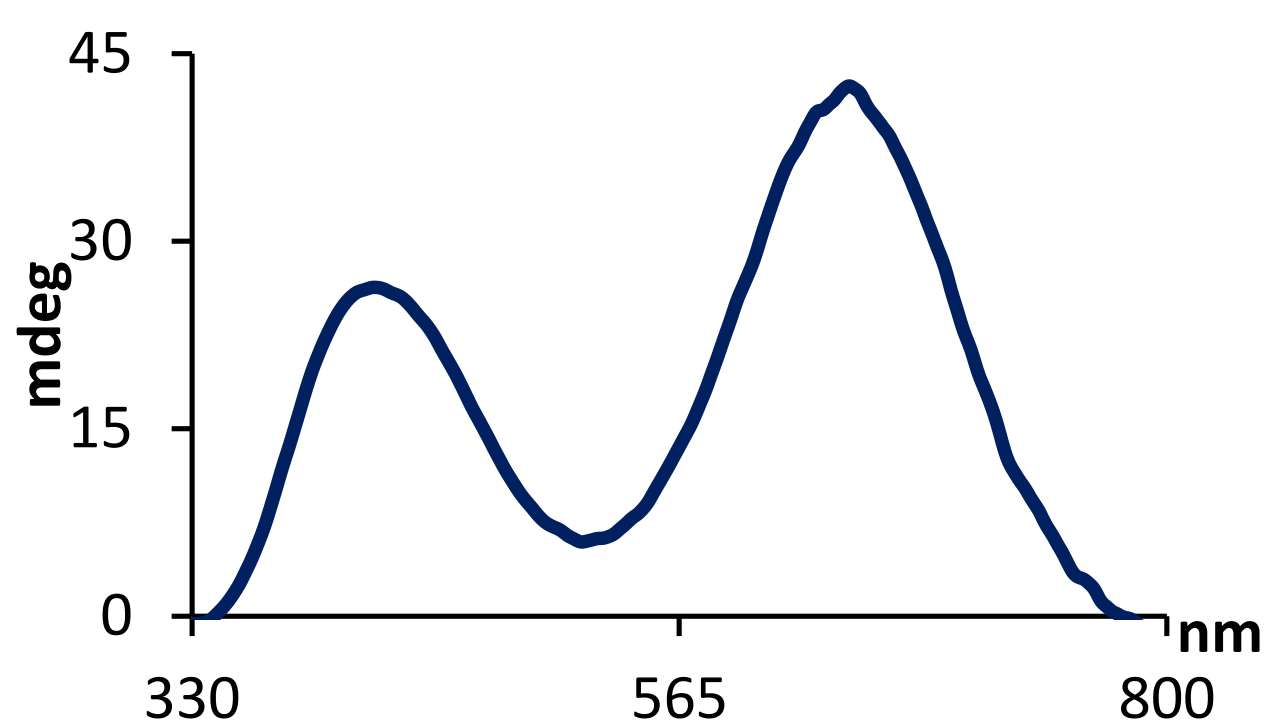

CD measurement was taken at 5.0 mM.

Figure S23. CD spectra obtained by applying sensor **2** to (*R*)-**7** (red) vs (*S*)-**7** (blue).

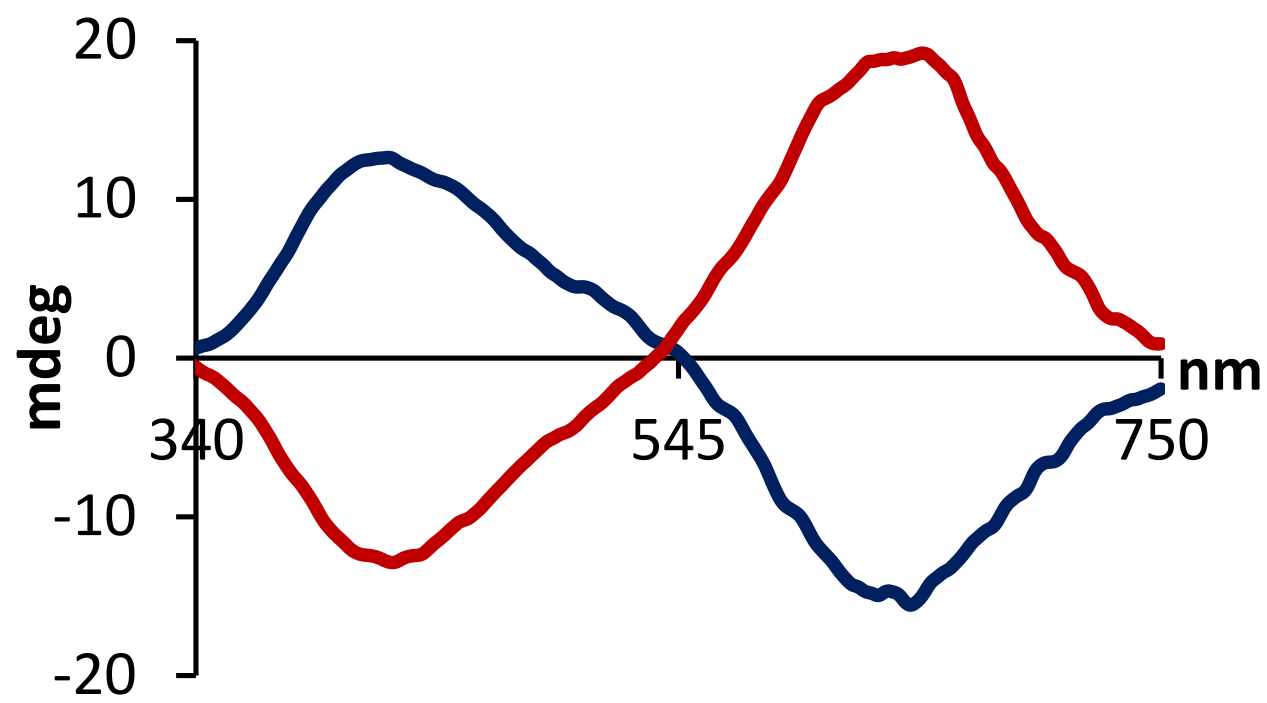

CD measurements were taken at 5.0 mM.

Figure S24. CD spectra obtained by applying sensor **2** to (*S*)-**8**.

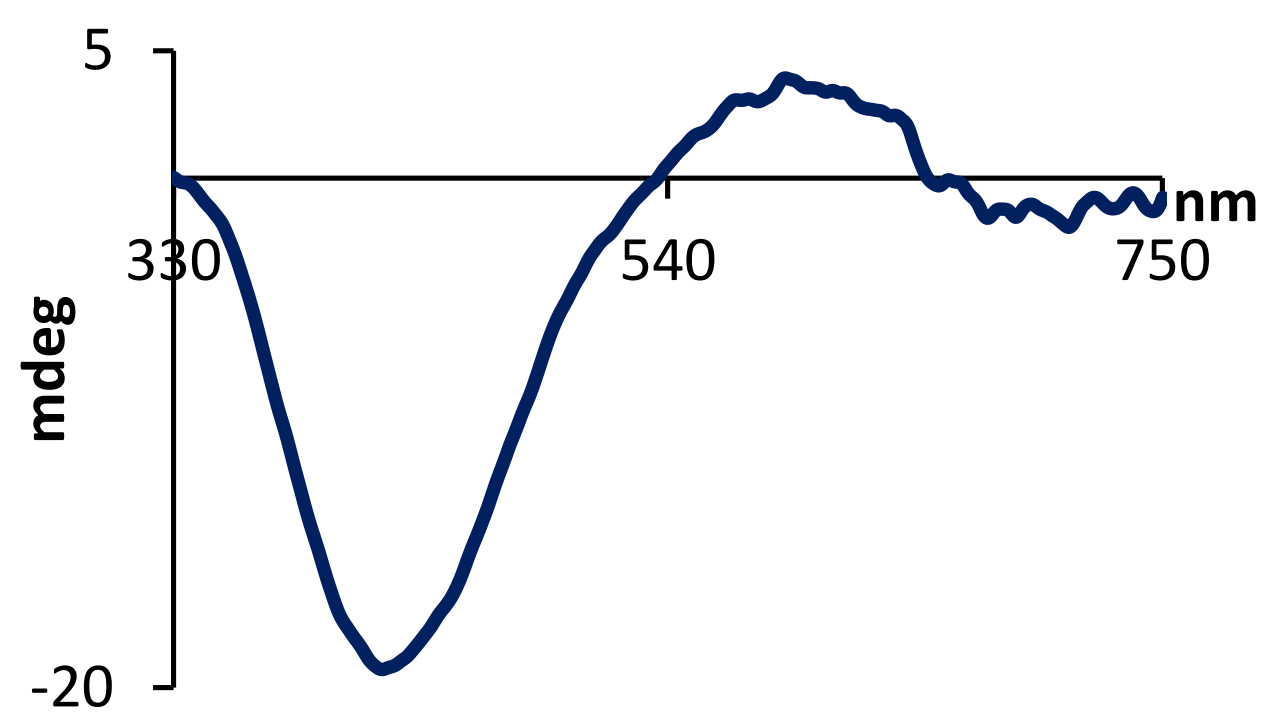

CD measurement was taken at 5.0 mM.

Figure S25. CD spectra obtained by applying sensor **2** to (*S*)-**9**.

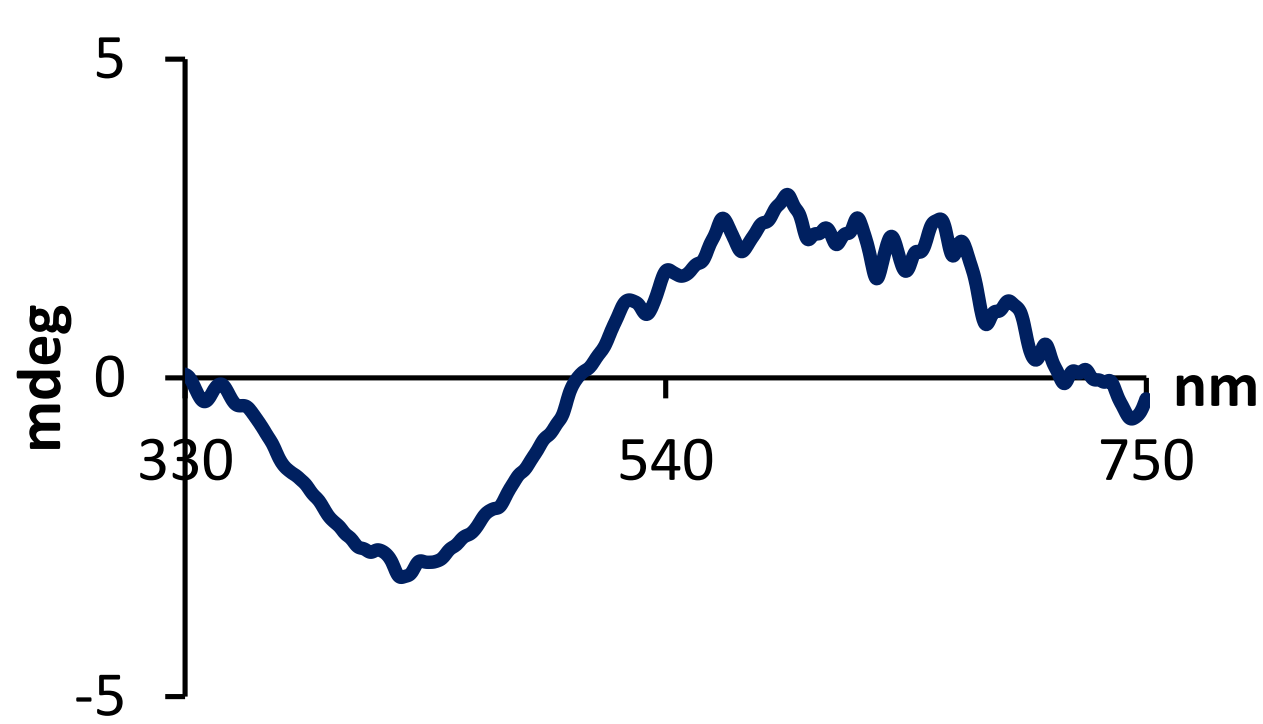

CD measurement was taken at 5.0 mM.

Figure S26. CD spectra obtained by applying sensor **2** to (*S*)-**10**.

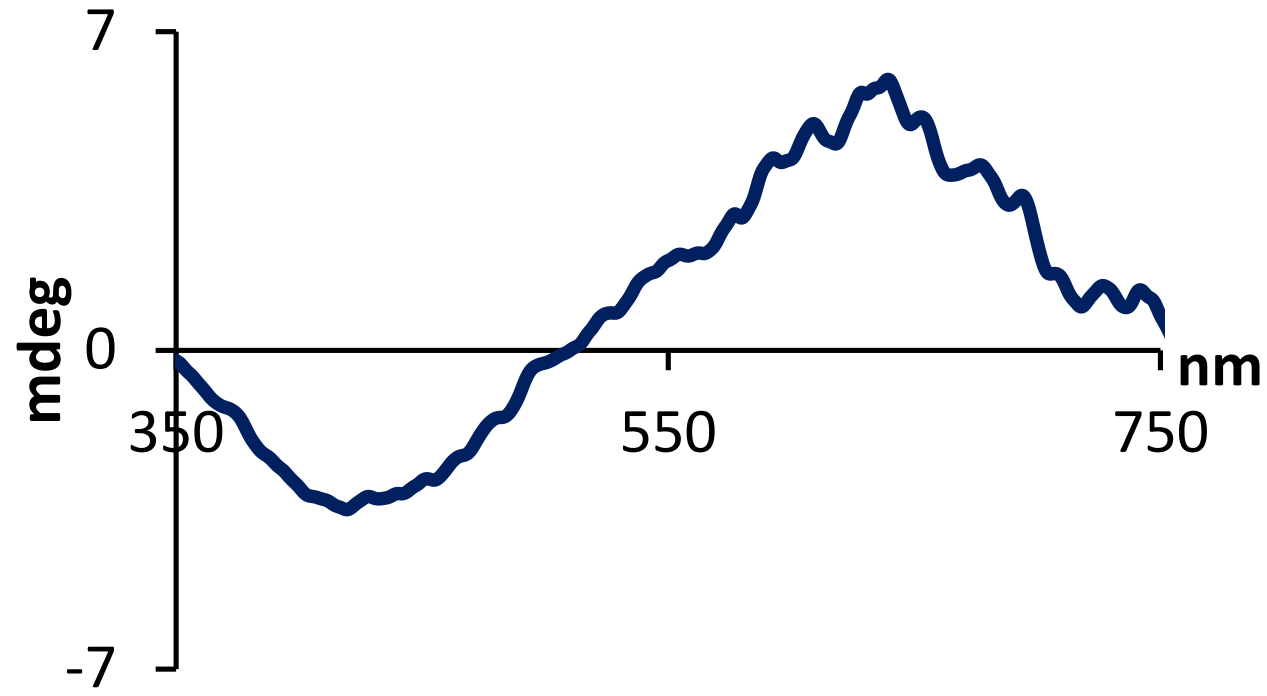

CD measurement was taken at 5.0 mM.

Figure S27. CD spectra obtained by applying sensor **2** to (*1R,4S*)-**11** (red) vs (*1S,4R*)-**11** (blue).

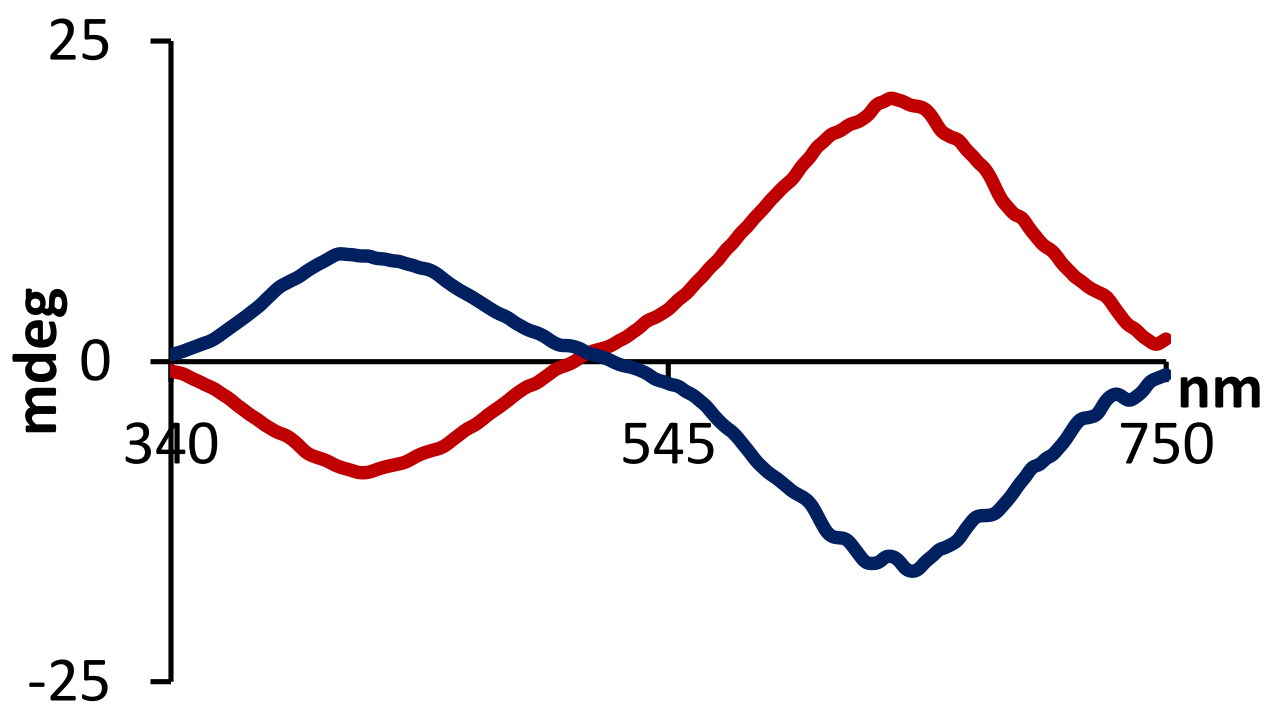

CD measurements were taken at 5.0 mM.

Figure S28. CD spectra obtained by applying sensor **2** to (*S*)-**12**.

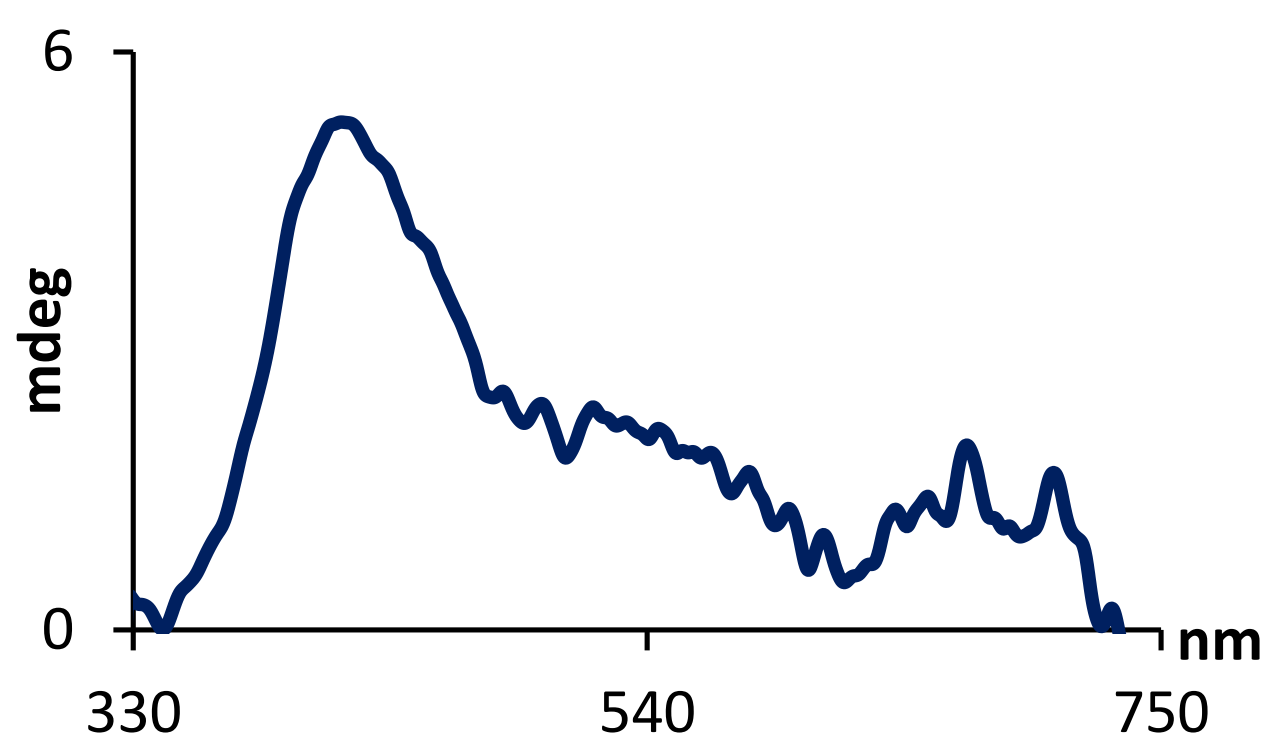

CD measurement was taken at 5.0 mM.

Figure S29. CD spectra obtained by applying sensor **2** to acid **13** (dihydroartemisinic acid).

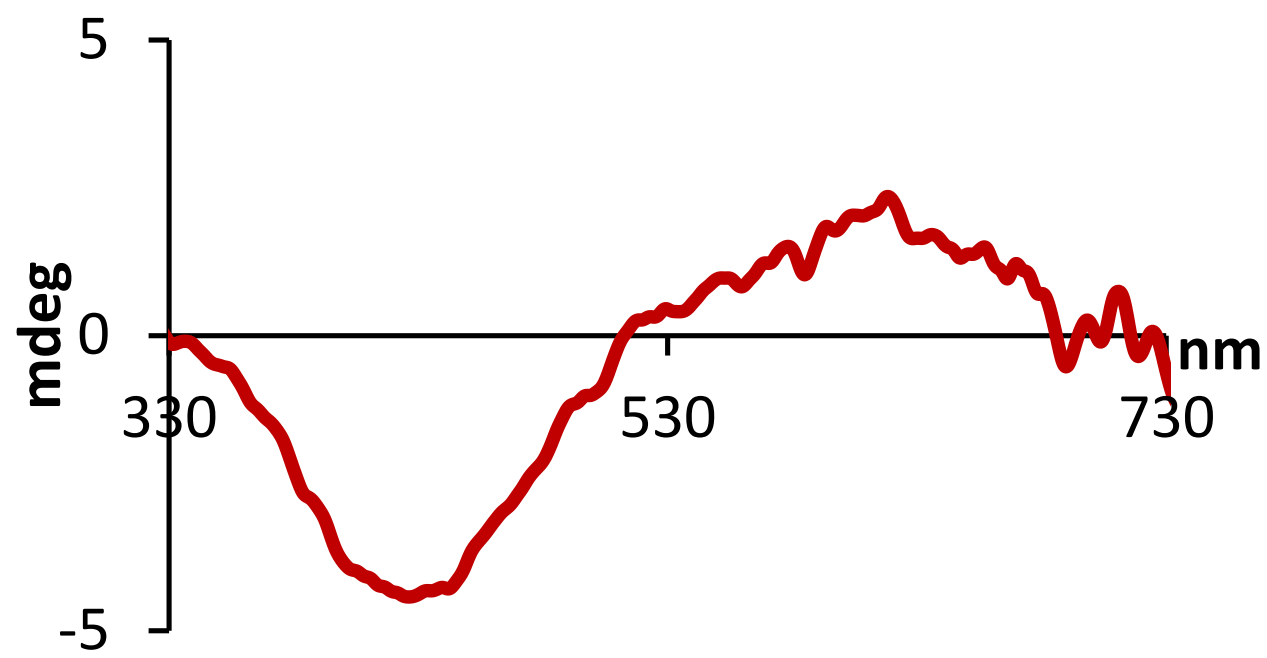

CD measurement was taken at 5.0 mM.

Figure S30. CD spectra obtained by applying sensor **2** to acid **14** (dehydroabietic acid).

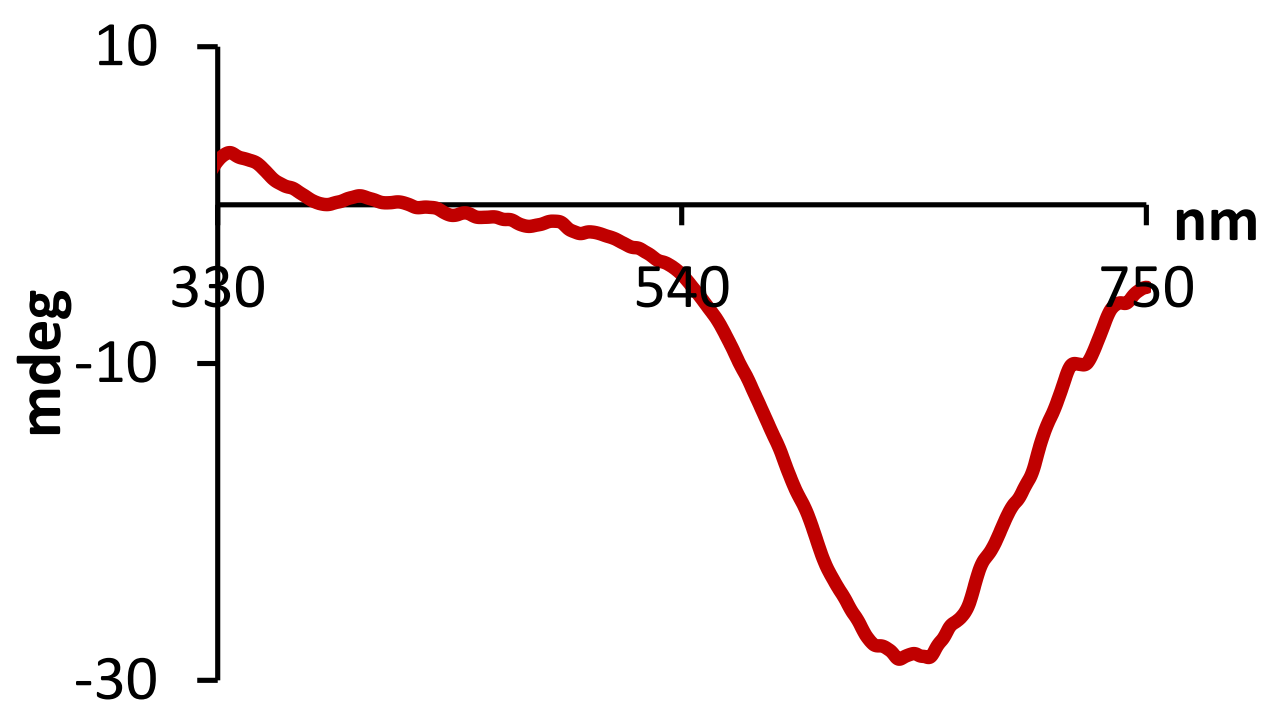

CD measurement was taken at 5.0 mM.

Figure S31. CD spectra obtained by applying sensor **2** to (1*R*,2*S*)-**15** (red) vs (1*S*,2*R*)-**15** (blue).

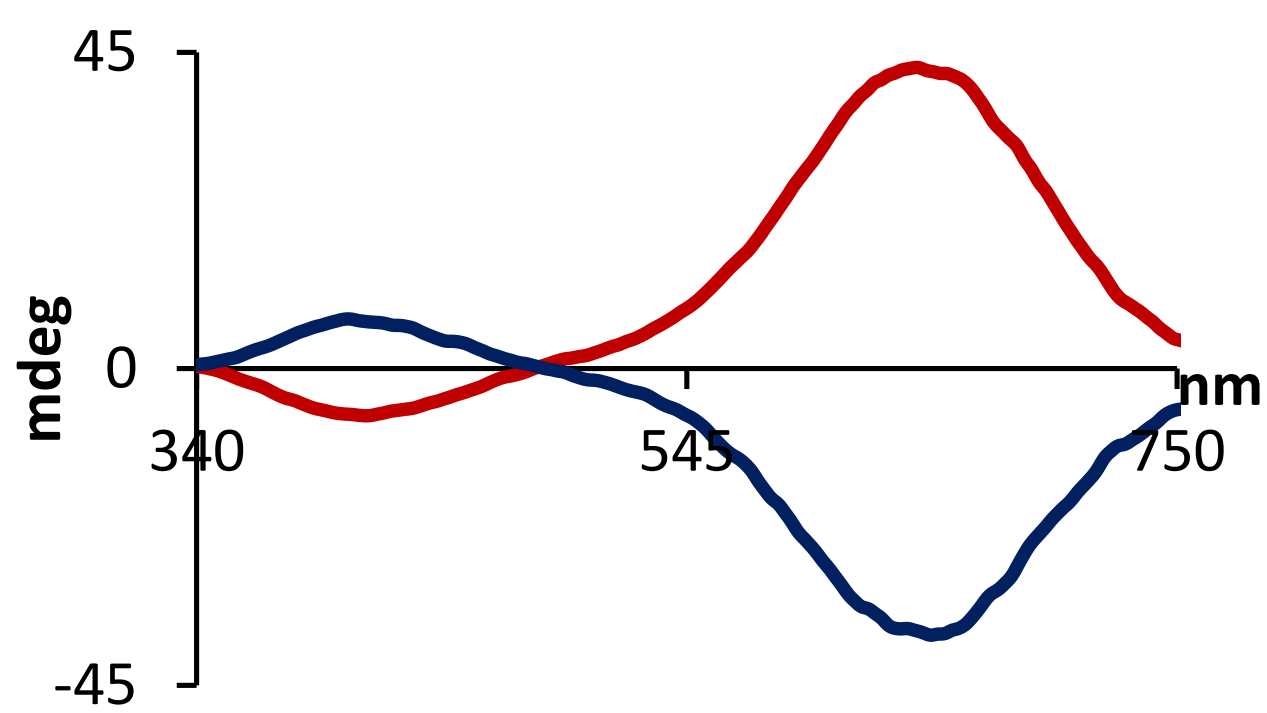

CD measurements were taken at 5.0 mM.

Figure S32. CD spectra obtained by applying sensor **2** to acid **16** (isosteviol).

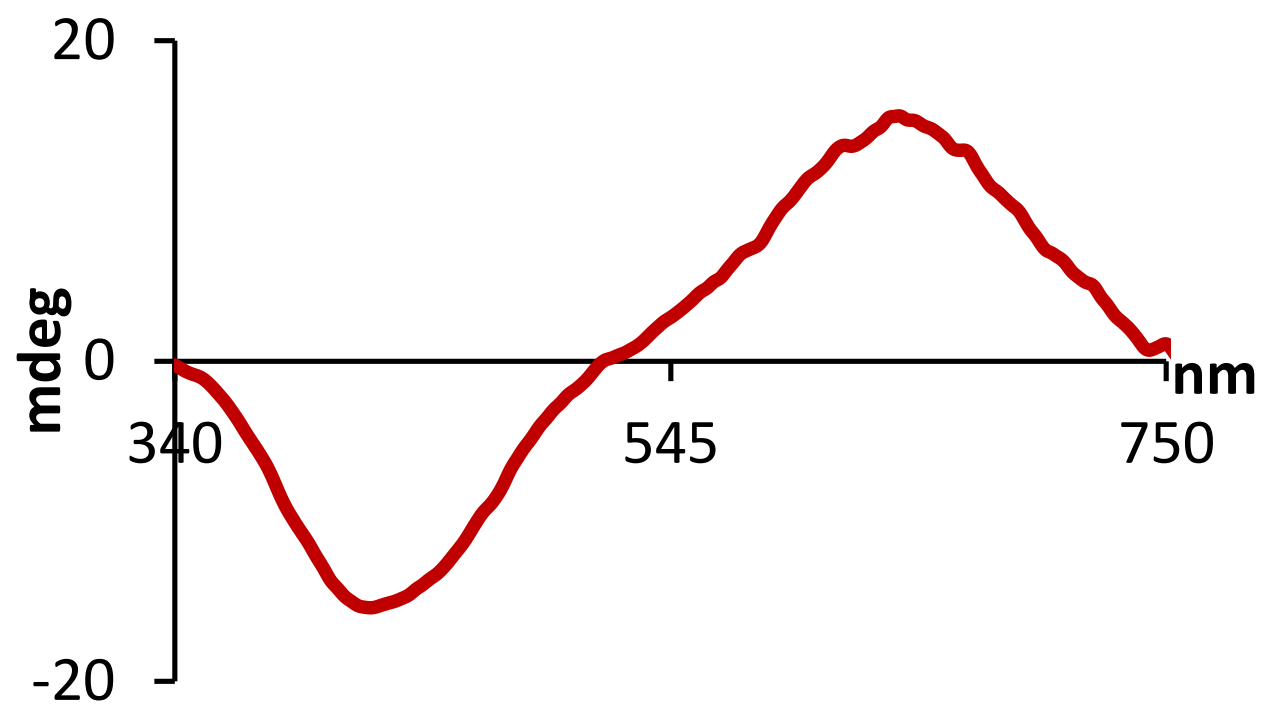

CD measurement was taken at 5.0 mM.

Figure S33. CD spectra obtained by applying sensor **2** to (*R*)-**17**.

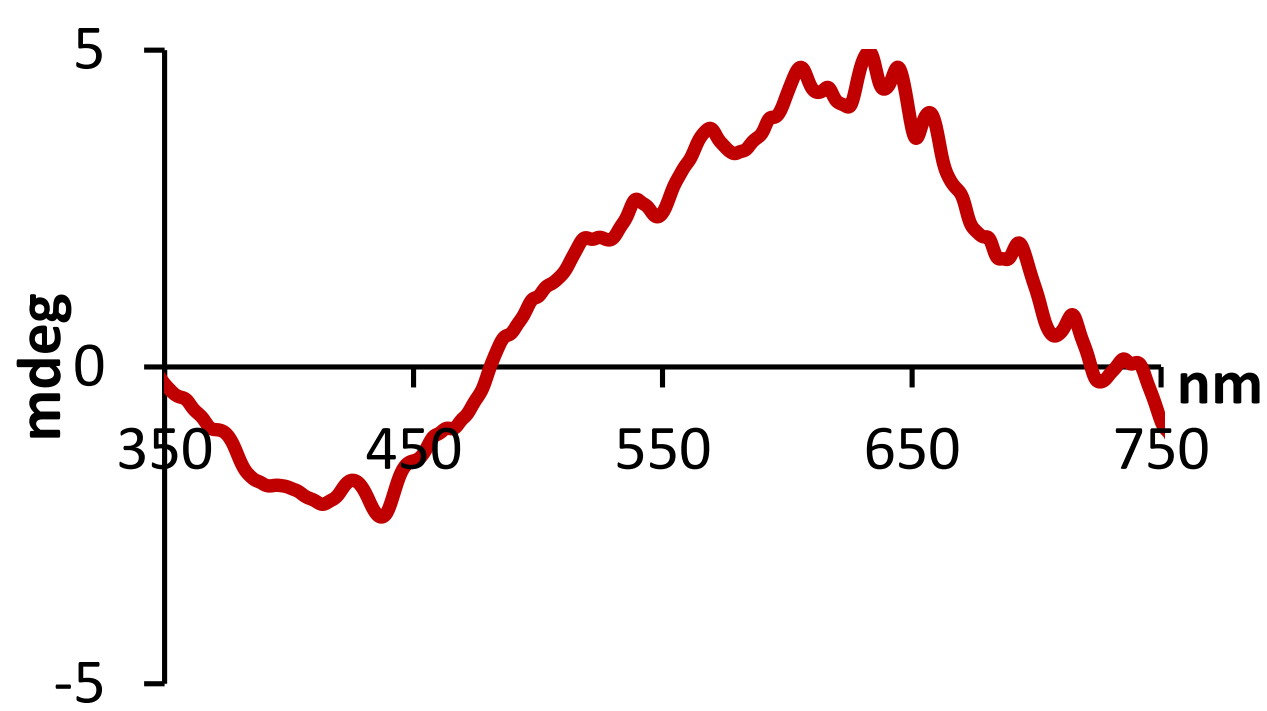

CD measurement was taken at 5.0 mM.

Figure S34. CD spectra obtained by applying sensor **2** to (*S*)-**18**.

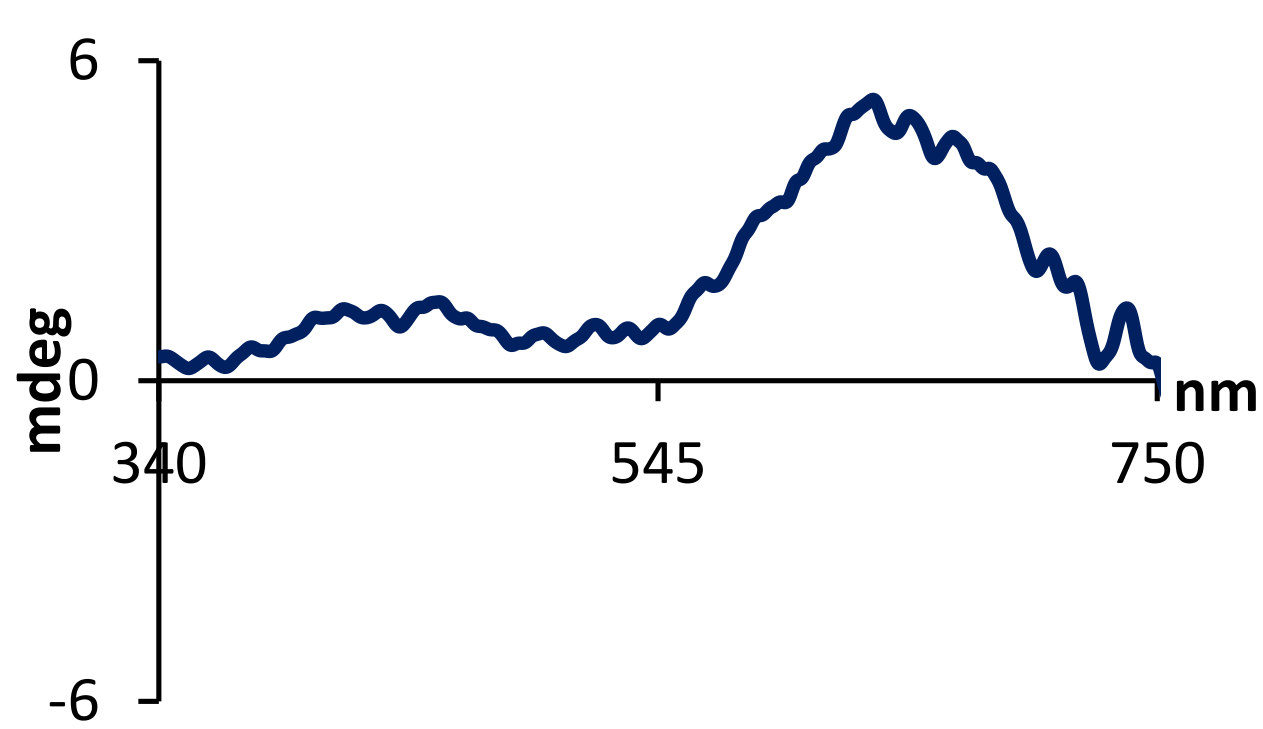

CD measurement was taken at 5.0 mM.

7. Carboxylic acid *er* sensing with sensor 2

Samples containing enantioenriched acid **5** were analyzed. To 1.6 mL of a 6.25 mM sensor **2** stock solution in *N,N*-dimethylformamide (DMF) were added 0.40 mL of a 25.0 mM solution of acid **5** in DMF with varying *ee*'s (−100, −80, −60, −40, −20, 0, +20, +40, +60, +80, and +100%), and 1.1 equivalents of K_2CO_3 as a solid. The mixtures were stirred for 1 hour under nitrogen. CD analysis was conducted directly with the reaction mixtures without further dilution. The CD amplitude at 420 and 645 nm were plotted against the enantiomeric excess of acid **5**, yielding linear correlations described by the equations: y = −0.1612x – 0.2002 (420 nm) and y = 0.4963x – 0.3288 (645 nm).

Figure S35. CD response of sensor **2** to scalemic samples of acid **5.**

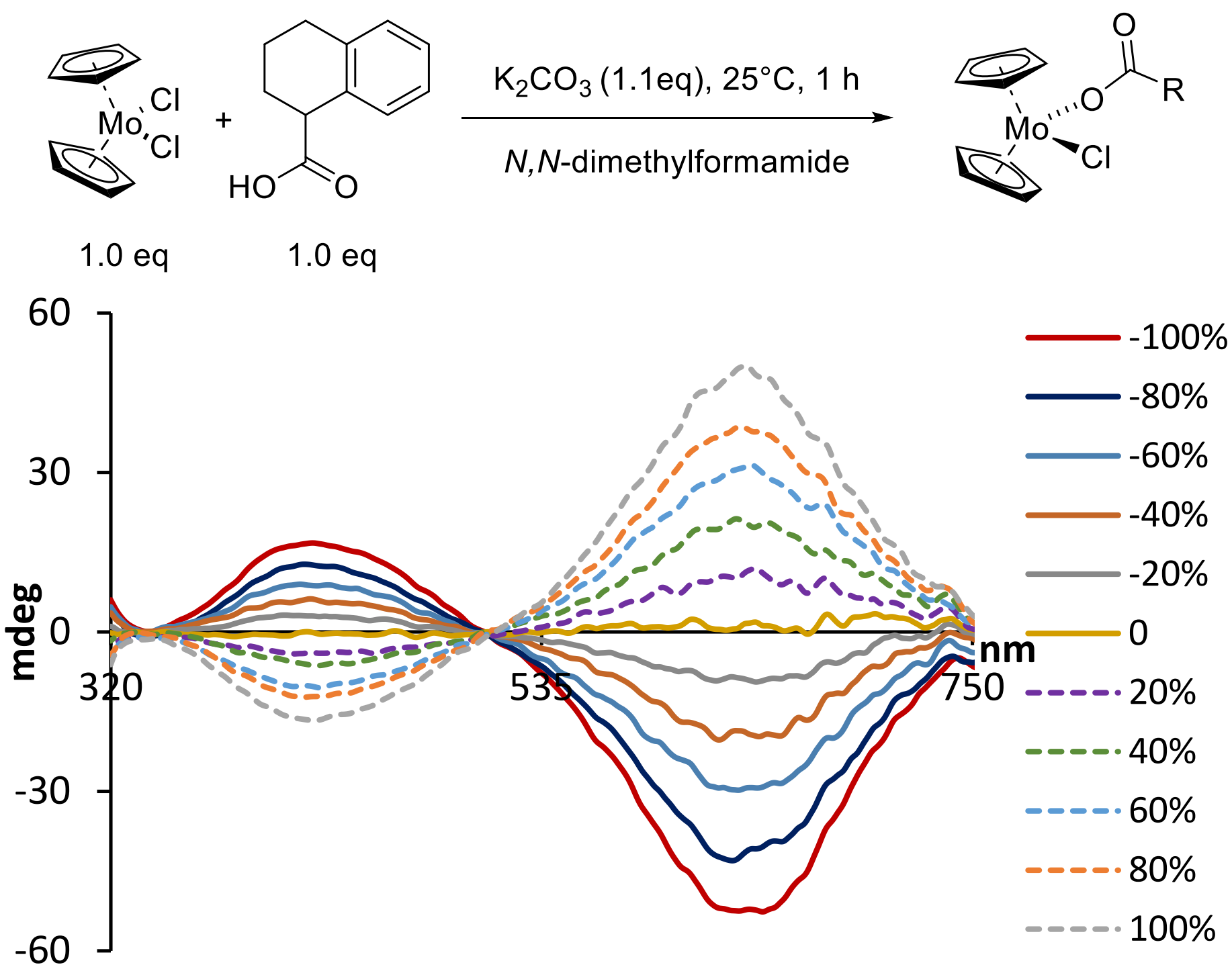

Figure S36. Induced CD amplitudes at 420 nm (left) and 645 nm (right) vs. sample *ee*. All CD measurements were taken at 5.0 mM.

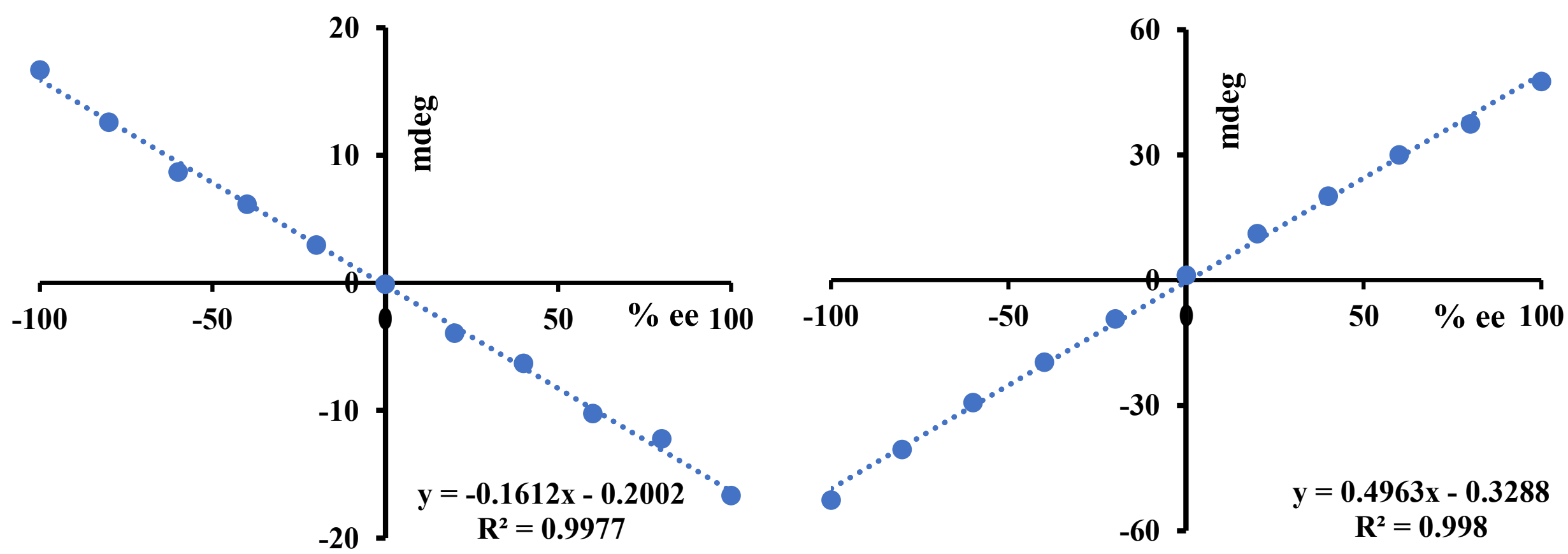

Enantiomeric ratio determination

Nine scalemic samples of acid **5,** each at a concentration of 10.0 mM and varying *er* were prepared in *N,N*-dimethylformamide. The samples were subjected to CD analysis as described above and the enantiomeric ratios were determined using the CD intensities measured at 645 nm and the corresponding calibration equation.

Table S2. Absolute configuration and enantiomeric ratio of samples of acid **5** analyzed with sensor **2**.

Sample	Sample composition		Sensing results	
	Abs. Config.	*er*	Abs. Config.	*er*
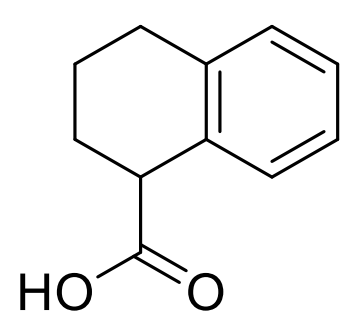	*R*	5.0:95.0	*R*	6.0:94.0
	R	12.5:87:5	*R*	12.8:87.2
	R	18.8:81.2	*R*	21.7:78.3
	R	25.0:75.0	*R*	22.2:77.8
	R	37.5:62.5	*R*	34.7:65.3
	R	45.0:55.0	*R*	43.8:56.2
	S	56.2:43.8	*S*	53.9:46.1
	S	62.5:37.5	*S*	60.4:39.7
	S	83.7:16.3	*S*	82.8:17.2

8. X-ray crystallography

To a solution of the carboxylic acid (*S*)-**5** (47.4 mg, 0.27 mmol) and K_2CO_3 (37.2 mg, 0.27 mmol) in DMF (4.0 mL) was added sensor **2** (40.0 mg, 0.13 mmol) under nitrogen. The reaction mixture was stirred at room temperature for 2 hours, filtered, and concentrated *in vacuo*. The residue was dissolved in CH_2Cl_2, and pentane was carefully layered onto the resulting solution (pentane/DCM, 1:1, v/v) to afford single crystals. Single crystal analysis was performed at 100 K using a Siemens platform diffractometer with a graphite monochromated Mo-Kα radiation (λ = 0.71073 Å). Data were integrated and corrected using the APEX 3 program. The structures were solved by direct methods and refined with full-matrix least-square analysis using SHELXL-2019/1 software. Non-hydrogen atoms were refined with anisotropic displacement parameter. Crystal data: $C_{43}H_{44}Cl_4Mo_2O_4$, M= 958.46, 0.287 x 0.266 x 0.164 mm^3, monoclinic, space group Cc, a = 12.0501(6) Å, b = 16.7464(8) Å, c = 19.5443(12) Å, Z =4. The crystal contains both carboxylic acid enantiomers. We were able to attribute this to racemization of the carboxylic acid (>98% *ee*) which occurs when the basic DMF solution is concentrated. Importantly, this does not affect the use of sensor **2** as described herein as racemization is not an issue in our optical assay. The CCDC number for the crystal is 2545012.

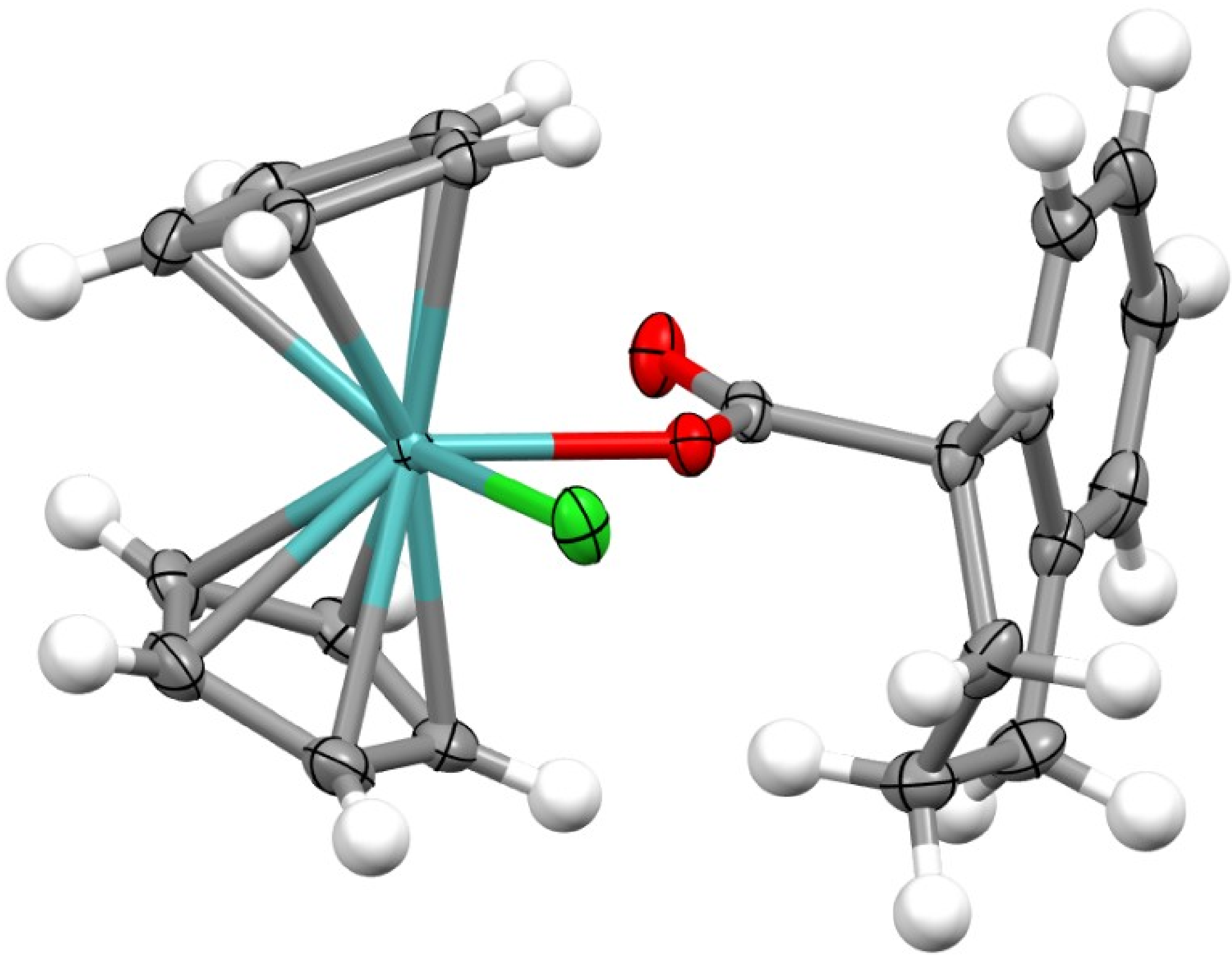

Figure S37. Thermal ellipsoids are displayed at the 50% probability level. For clarity, a co-crystallizing dichloromethane molecule is omitted.

To a mixture of the carboxylic acid (*S*)-**5** (56.4 mg, 0.32 mmol) and K_2CO_3 (44.2 mg, 0.32 mmol) in dichloromethane (4.0 mL) was added sensor **1** (40.0 mg, 0.16 mmol) under nitrogen. The reaction mixture was stirred at room temperature for 3 hours and filtered. The filtrate was transferred to an open 4 mL vial, which was subsequently placed inside a sealed 20 mL vial containing pentane (4.0 mL) to afford single crystals. Single crystal analysis was performed at 100 K using a Siemens platform diffractometer with a graphite monochromated Mo-Kα radiation (λ = 0.71073 Å). Data were integrated and corrected using the APEX 3 program. The structures were solved by direct methods and refined with full-matrix least-square analysis using SHELXL-2019/1 software. Non-hydrogen atoms were refined with anisotropic displacement parameter. Crystal data: $C_{32}H_{32}O_4Ti$, M= 528.47, 0.346 x 0.168 x 0.064 mm^3, monoclinic, space group $P2_1$, a = 14.2659(11) Å, b = 12.8063(10) Å, c = 14.7070(12) Å, Z =4. The CCDC number for this crystal is 2549273.

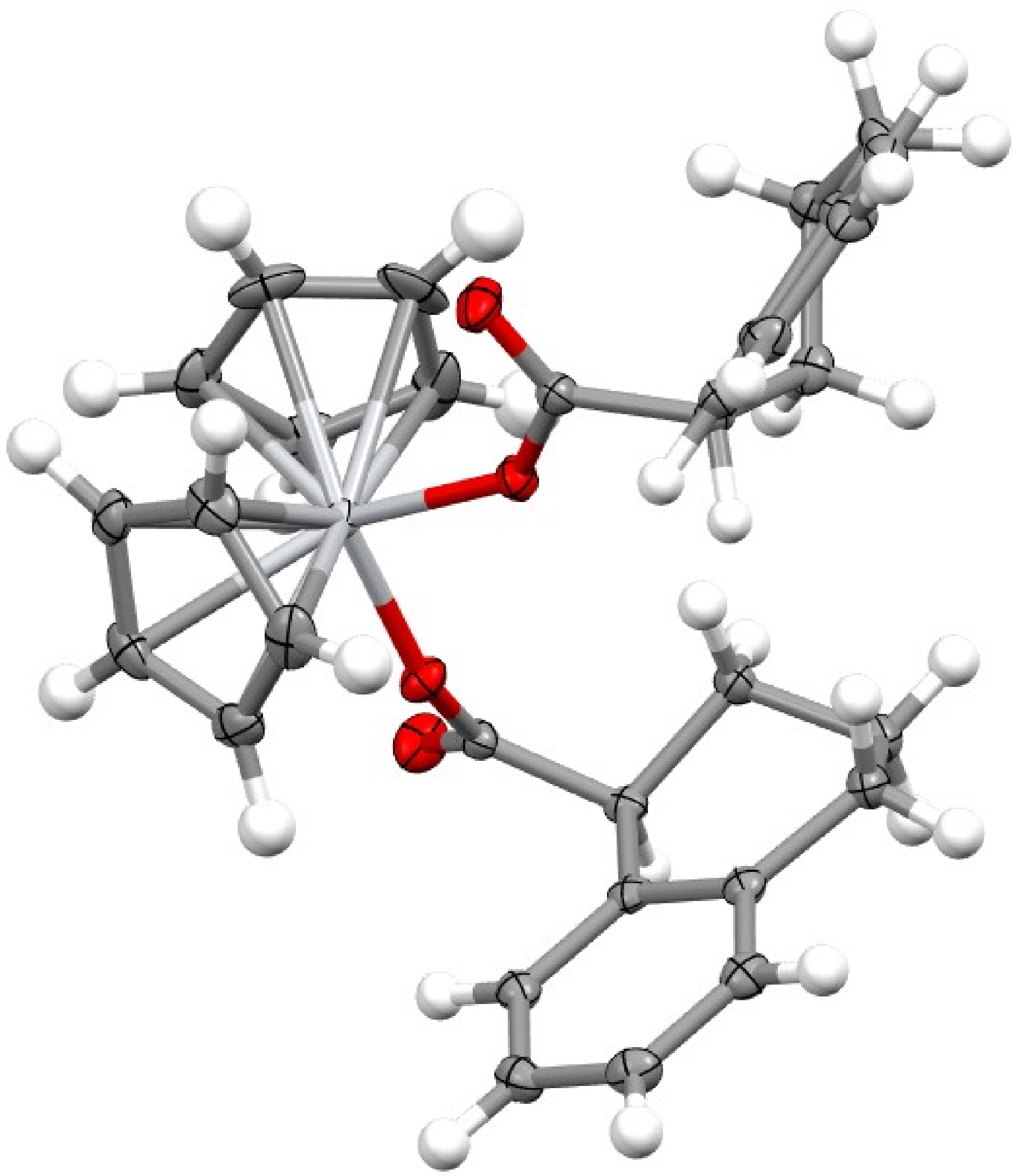

Figure S38. Thermal ellipsoids are displayed at the 50% probability level. For clarity, only one molecule is shown.

9. CD titration and stability experiments

To determine if the carboxylate binding is reversible and allows formation of heterochiral titanium complexes, we conducted several CD titration experiments. We prepared the homochiral complex Cp_2Ti[(*S*)-**20**]$_2$ following our general assay protocol. This gave the expected CD induction with a strong positive signal, Figure 6. One equivalent of the (*R*)-acid was then added and after stirring for several hours the CD amplitude decreased to the value predicted for a 33% *ee* mixture. The CD essentially disappeared upon addition of a second (*R*)-**20** equivalent as expected for a racemic mixture at equilibrium. This was reproduced with the opposite homochiral Cp_2Ti[(*R*)-**20**]$_2$ complex. These titration experiments clearly demonstrate that the system is dynamic and undergoes reversible ligand exchange. The CD changes are in agreement with the presence of a heterochiral dicarboxylate complex that is formed by stepwise replacement of one carboxylate at a time as simultaneous displacement of two (*S*)-carboxylates by two (*R*)-enantiomers is unlikely.

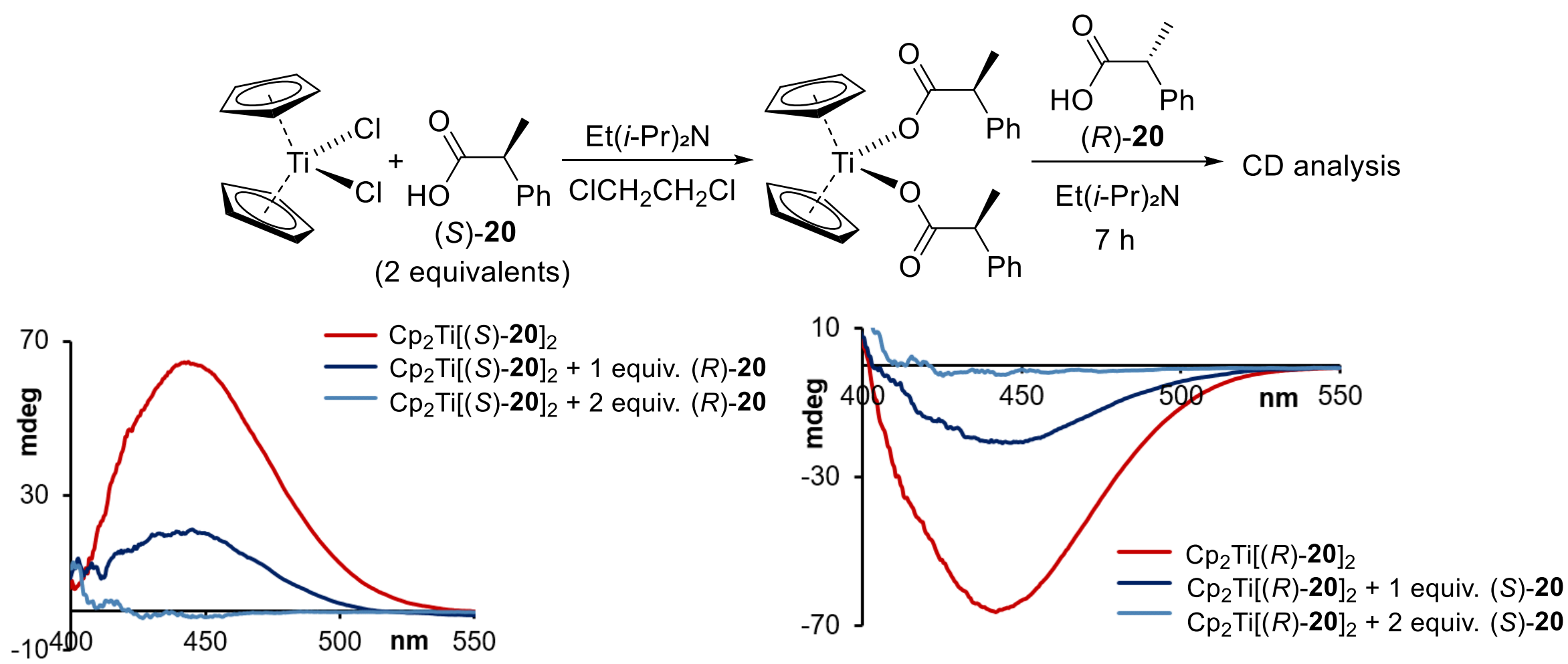

Figure S39. CD titration experiments with Cp_2Ti[(*S*)-**20**]$_2$ (left) and Cp_2Ti[(*R*)-**20**]$_2$ (right).

We conducted a CD time-study using either the acid (1*R*,4*S*)-**11** or (*S*)-**5**. The CD spectra were measured every hour, and the CD intensities remained unchanged. This shows that the reaction is completed within 1 hour and the signal remains stable for up to 5 hours.

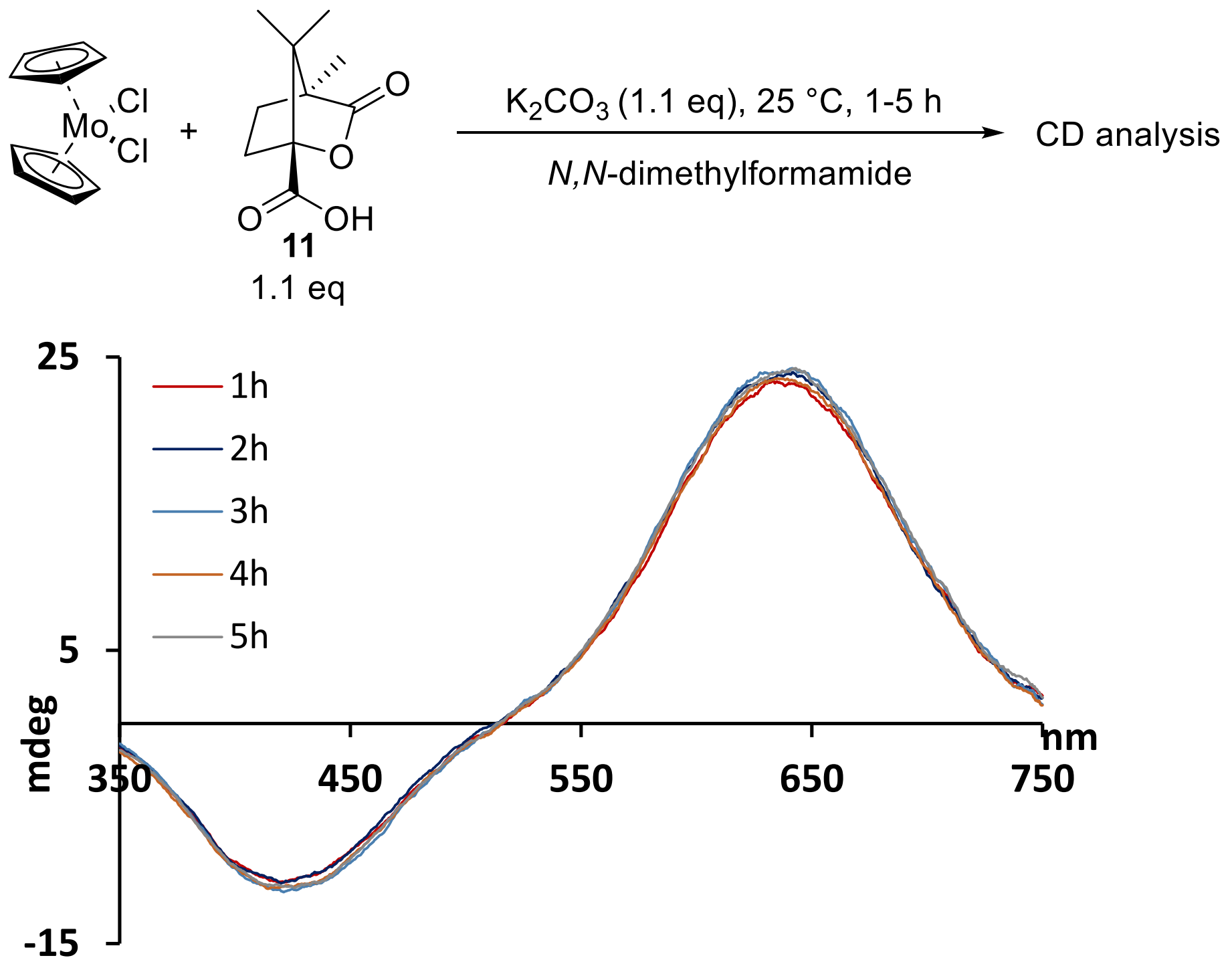

Figure S40. Stability of the CD signal obtained with (1*R*,4*S*)-**14.**

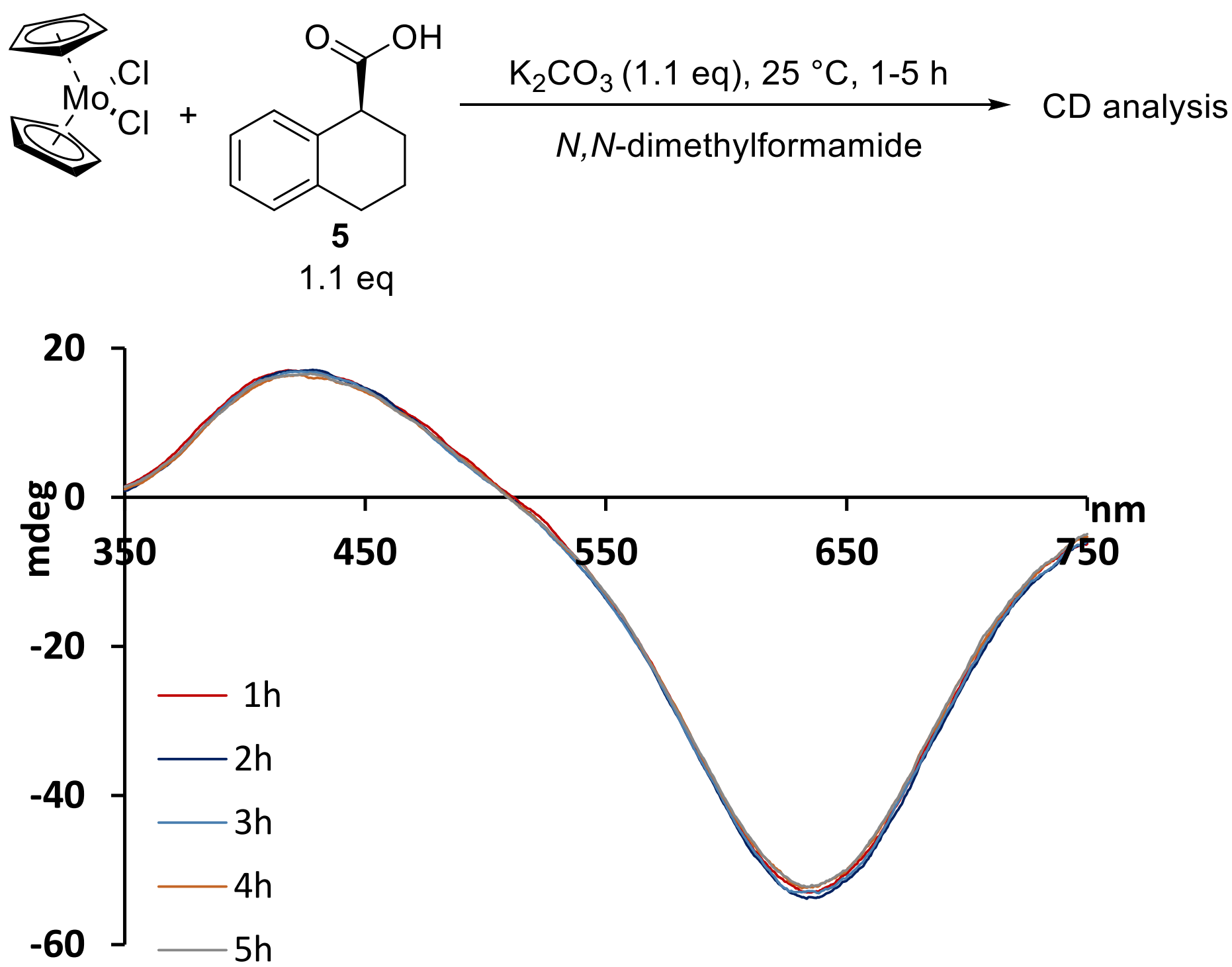

Figure S41. Stability of the CD signal obtained with (*S*)-**5.**

10. HR-MS results

The carboxylate complexes were formed in DMF or dichloromethane as described above. After filtration, the solvent was then removed *in vacuo*. The residues were then subjected to HR-MS analysis using electron spray ionization time-of-flight (ESI-TOF) spectrometry.

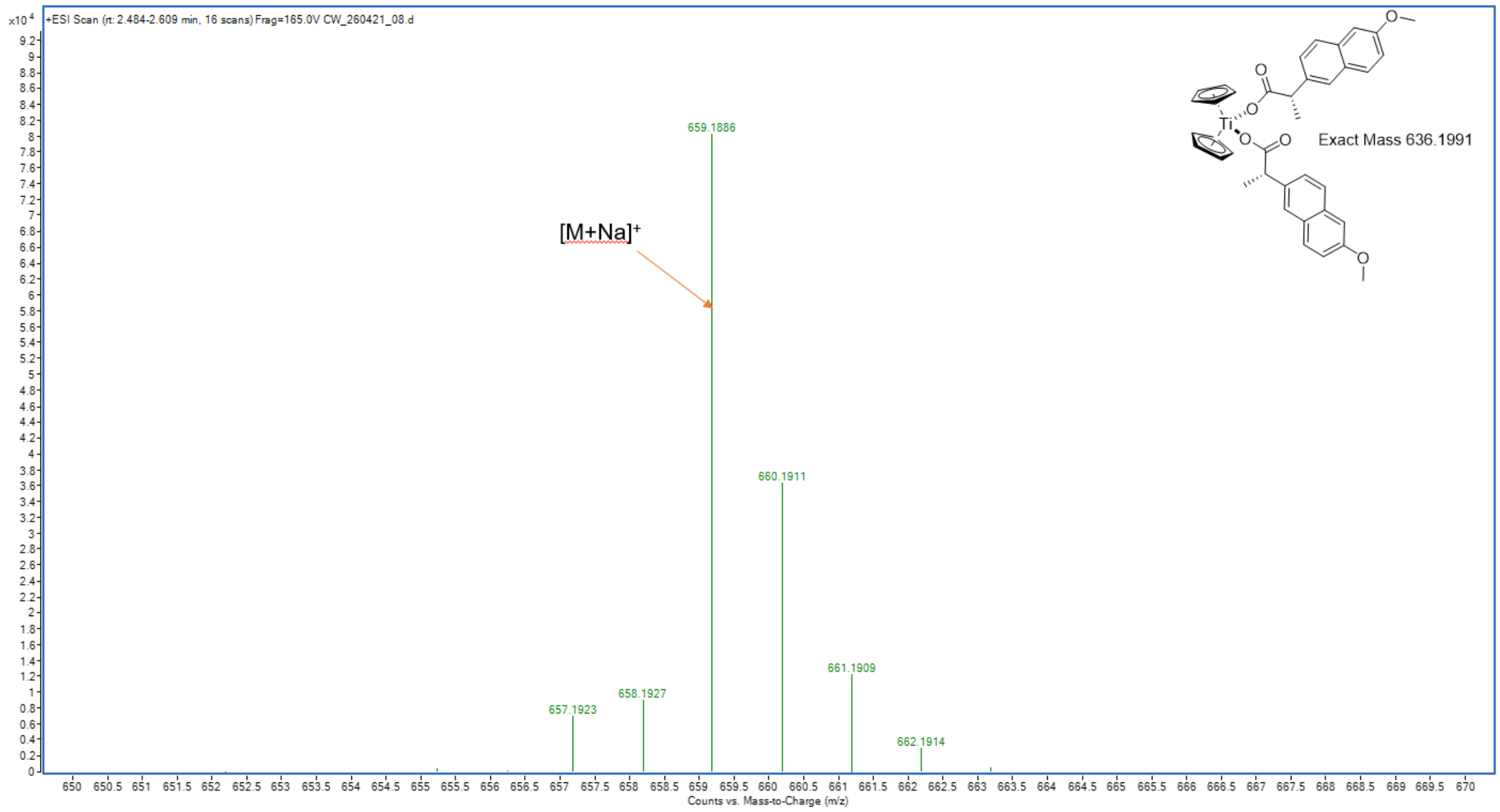

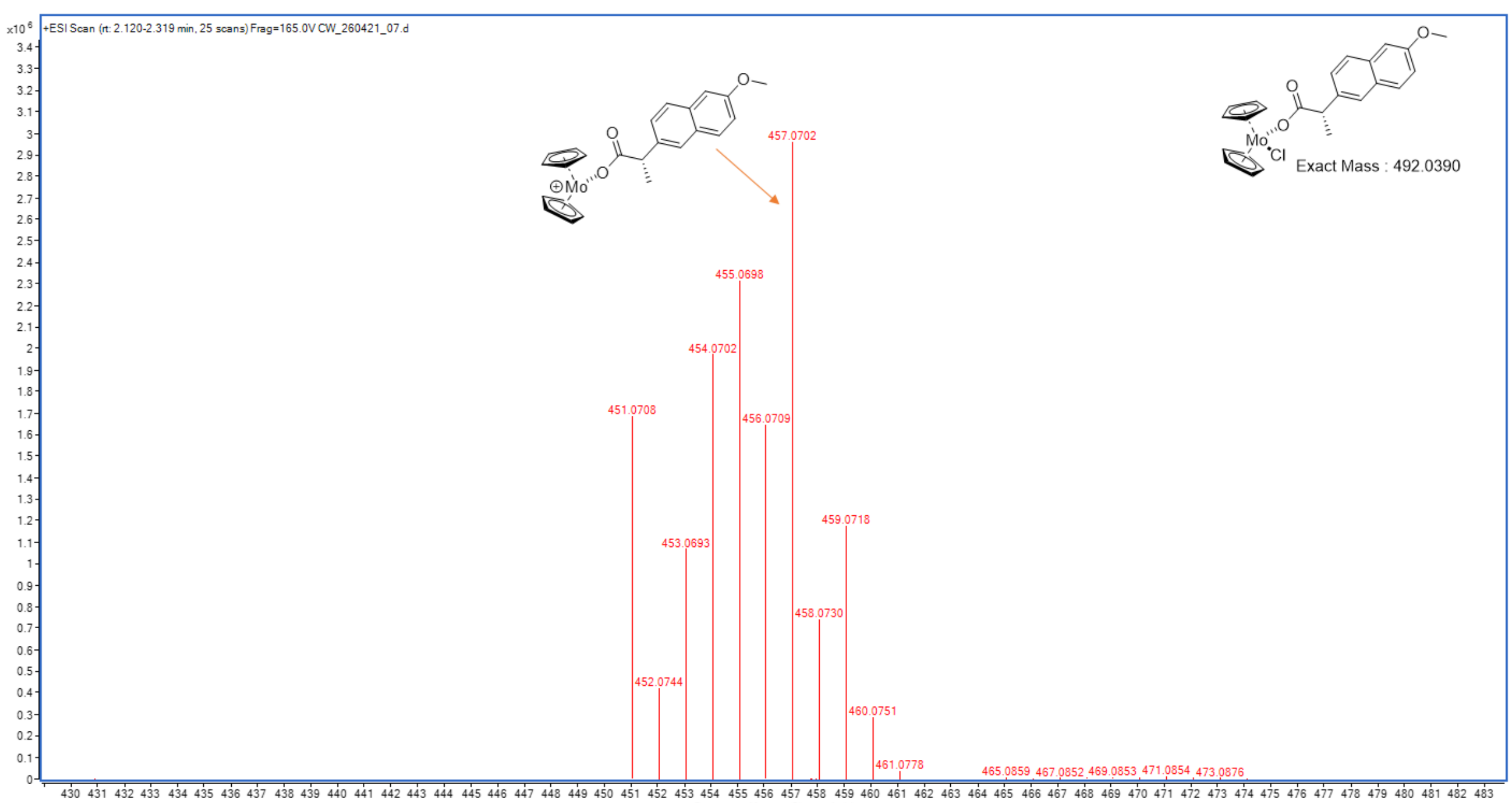

Supplement: Supplementary file 1 [file jo6c01097_si_001.pdf]
